# Alterations in Gut Microbiota Composition in Patients with COVID-19: A Pilot Study of Whole Hypervariable 16S rRNA Gene Sequencing

**DOI:** 10.3390/biomedicines11020367

**Published:** 2023-01-27

**Authors:** Dorota Mańkowska-Wierzbicka, Joanna Zuraszek, Adrianna Wierzbicka, Marcin Gabryel, Dagmara Mahadea, Alina Baturo, Oliwia Zakerska-Banaszak, Ryszard Slomski, Marzena Skrzypczak-Zielinska, Agnieszka Dobrowolska

**Affiliations:** 1 Department of Gastroenterology, Dietetics and Internal Diseases, Poznan University of Medical Sciences, 60-355 Poznan, Poland; 2 Institute of Human Genetics, Polish Academy of Sciences, 60-479 Poznan, Poland

**Keywords:** COVID-19, gut microbiota, gut–lung axis, dysbiosis, recovered COVID-19 patient, microbiota, gastrointestinal tract, gut microbiome

## Abstract

It is crucial to consider the importance of the microbiome and the gut–lung axis in the context of SARS-CoV-2 infection. This pilot study examined the fecal microbial composition of patients with COVID-19 following a 3-month recovery. Using for the first time metagenomic analysis based on all hypervariable regions (V1-V9) of the 16S rRNA gene, we have identified 561 microbial species; however, 17 were specific only for the COVID-19 group (*n* = 8). The patients’ cohorts revealed significantly greater alpha diversity of the gut microbiota compared to healthy controls (*n* = 14). This finding has been demonstrated by operational taxonomic units (OTUs) richness (*p* < 0.001) and Chao1 index (*p* < 0.01). The abundance of the phylum *Verrucomicrobia* was 30 times higher in COVID-19 patients compared to healthy subjects. Accordingly, this disproportion was also noted at other taxonomic levels: in the class *Verrucomicrobiae*, the family *Verrucomicrobiaceae,* and the genus *Akkermansia*. Elevated pathobionts such as *Escherichia coli*, *Bilophila wadsworthia,* and *Parabacteroides distasonis* were found in COVID-19 patients. Considering the gut microbiota’s ability to disturb the immune response, our findings suggest the importance of the enteric microbiota in the course of SARS-CoV-2 infection. This pilot study shows that the composition of the microbial community may not be fully restored in individuals with SARS-CoV-2 following a 3-month recovery.

## 1. Introduction

Coronavirus disease 2019 (COVID-19) is caused by the infection of novel, highly contagious severe acute respiratory syndrome coronavirus 2 (SARS-CoV-2). The virus quickly spread across the continents, and in March 2020, a pandemic was officially declared by the World Health Organization (WHO) [1]. In recent epidemiological reports, the burden of comorbidities, immunocompromised states, older age, and obesity have been strongly associated with the disease’s severity [2,3]. COVID-19 most commonly affects the pulmonary system, with the most frequent clinical presentation involving dyspnea, fever, cough, and sore throat [4]. Clinical manifestation is diverse and can range from the absence of symptoms to multi-organ failure and death [5]. In advanced cases, a patient’s clinical course can be complicated by pneumonia, acute respiratory distress syndrome, acute hypoxic respiratory failure, and death [6,7]. It is worth highlighting that SARS-CoV-2-induced intense cytokine and chemokine response (cytokine storm syndrome) plays an important role in the development of acute respiratory distress syndrome (ARDS) and multi-organ dysregulation [8]. Although respiratory symptoms are most predominantly reported amongst patients with COVID-19, extrapulmonary symptoms from gastrointestinal (GI), hepatobiliary, cardiovascular, renal, and neurological systems are also noted [5]. Moreover, following recovery, a subgroup of patients may develop autoinflammatory symptoms including multisystem inflammatory syndrome and Kawasaki-like disease in children [9,10].

Studies have provided direct evidence that the spike protein of SARS-CoV-2 serves as a crucial mode of entry into the host organism [11]. It binds to angiotensin-converting enzyme 2 (ACE2) and is cleaved by transmembrane cellular serine protease (TMPRSS2) [12]. This interaction increases the production and replication of infectious de novo virus molecules [13]. These receptors are extensively expressed in the lungs, heart, kidneys, and gut epithelial cells. This raises the possibility that ACE2 modulates intestinal immunity and the composition of gut microbiota by maintaining amino-acid transporters [14]. It is, therefore, crucial to consider the importance of the microbiome and the gut–lung axis in the context of SARS-CoV-2 infection. Emerging data revealed that COVID-19 infection cannot be detected merely in oral swabs but also in fecal specimens and anal/rectal swabs, suggesting crosstalk between the gut and the lungs [15,16,17]. Growing evidence showed that adequate interaction between the microbiota in the gut and the lungs plays a pivotal role in maintaining homeostasis. Accordingly, any alterations in the microbial community increase host vulnerability and disease outcomes and promote states of chronic low-grade inflammation [18]. Notably, our previous investigation has also confirmed the impact of dysbiosis on the inflammatory process accompanying the disease [19].

Considering the points raised, the aim of this study is to discuss the disturbances of gut microbiota based on a complete hypervariable sequence of the 16S rRNA gene in patients following three months of recovery from SARS-CoV-2 juxtaposed with healthy controls. Additionally, our goal is to verify obtained results by performing a meta-analysis with similar publicly available data.

## 2. Materials and Methods

### 2.1. Study Subject and Design

This study was conducted according to the Declaration of Helsinki and was approved by the local Ethics Committee of Poznan University of Medical Sciences (resolution no. 95/22, approved on 17 February 2022). In this prospective investigation, a total of 22 individuals—8 subjects with recovered COVID-19 infection and 14 healthy controls—were recruited. The diagnosis of SARS-CoV-2 was confirmed by a positive SARS-CoV-2 real-time reverse transcription-polymerase chain reaction (RT-PCR) throat swab. All participants provided written informed consent to participate in this research and agreed to the publication of the study results. Fecal sample collection was conducted between January 2021 and March 2021. Inclusion criteria for SARS-CoV-2 recovered subjects included age > 18 years and documented recovery history, whereas exclusion criteria encompassed asymptomatic patients. Both patients and healthy individuals have neither received probiotic or antibiotic treatment within six months nor had any severe additional diseases (including GI disorders, cancer, and diabetes).

### 2.2. Metaprofiling and Data Processing

#### 2.2.1. DNA Extraction

Microbial genomic DNA was extracted from the stool samples using the QIAamp PowerFecal Pro DNA Kit (QIAGEN, Hilden, Germany) according to the manufacturer’s protocol. Fecal samples were collected and frozen immediately at −80 °C until DNA extraction. The isolated bacterial material was quantified and characterized using a spectrophotometer NanoDrop™ 2000 (Thermo Fisher Scientific, Inc., Waltham, MA, USA).

#### 2.2.2. Next-Generation Sequencing (NGS)

Bacterial 16S ribosomal RNA (rRNA) gene and fungal internal transcribed spacer (ITS) libraries for metaprofiling analysis were prepared using QIAseq 16S/ITS Panels Reagent Kit Screening Panel (QIAGEN) according to the manufacturer’s protocol. The first step involved the amplification of microbial target DNA sequences in three multiplexed PCRs: (1) V1–V2, V4–V5, ITS; (2) V2–V3, V5–V7; and (3) V3–V4, V7–V9. The obtained amplification products were combined, mixed, and then cleaned using the magnetic QIAseq Beads (QIAGEN) and subjected to indexing with unique oligonucleotide sequences placed on a commercial plate QIAseq 16S/ITS 96-Index I (QIAGEN). Amplicon libraries were purified, and the following were checked using a High Sensitivity DNA Assay on an Agilent 2100 Bioanalyzer System (Agilent, Santa Clara, CA, USA) and pooled to one collective NGS library with a final concentration of 6 pM. Bacteriophage Φ-X174 (PhiX) control library was added at 3%. Sequencing was performed on an Illumina MiSeq platform using a MiSeq Reagent Kit v3 (600 cycles).

#### 2.2.3. Bioinformatic Analysis

The 16S rRNA gene and ITS sequences were obtained and processed using a CLC Genomic Workbench 11.0 and CLC Microbial Genomics Module 1.2. (Qiagen Bioinformatics, Aarhus, Denmark). Reads were demultiplexed and trimmed. Such data have been grouped under operational taxonomic units (OTUs), relative to the SILVA v119 97% 16S rRNA database. Bioinformatics processing made it possible to obtain and export Excel files with a qualitative–quantitative list of microorganisms on different taxonomic levels: phylum, class, order, family, genus, and species. Alpha diversity was calculated using the OTUs richness, the Chao1, Shannon entropy, and Simpson index. Beta diversity was specified based on a Bray–Curtis dissimilarity matrix and with unweighted and weighted Unifrac distance.

### 2.3. Statistical Analysis

The normality of demographic data and microbiota percentage abundance levels at different taxonomic levels were assessed using the Shapiro–Wilk test. For nominal data, the chi-square test and t-test for independent groups were used. When the data did not follow the normal distribution pattern, the non-parametric Mann–Whitney test for median values was applied for the comparison. Furthermore, a correction concerning the multiple hypothesis testing was performed. The adjusted *p*-values were calculated according to the Benjamini–Hochberg procedure. To investigate the relationship between the particular microbial phyla, principal component analysis (PCA) was performed. Calculations were carried out using PQStat 1.8.2 (PQStat Software, Poznan, Poland) and Graph Pad Prism 8 (GraphPad Software, Inc., San Diego, USA) software, and all tests were considered significant at *p* < 0.05.

### 2.4. Search and Selection of Studies for Integrative Research

We conducted a systematic search of studies in PubMed, Embase, The Cochrane Library, and the Web of Science database including the keywords (‘Intestinal’ [MeSH] OR ‘Gut’ [MeSH]) AND (‘Bacteria’ [MeSH] OR ‘Microbiota’ [MeSH] OR ‘Microflora’ [MeSH] OR ‘Microbiome’ [MeSH] OR ‘Dysbiosis’ [MeSH]) AND (‘COVID-19’ [MeSH] OR ‘COVID19’ [MeSH] OR ‘SARS-CoV-19’ [MeSH] OR ‘SARS-CoV-19’ [MeSH]) AND (‘16S rRNA’ [MeSH] OR ‘NGS’ [MeSH]) with the following filters: publication date from 1 January 2020–25 April 2022, Humans, Adult. We also performed manual searches. The last search update was conducted on 25 April 2022. Including criteria were case–control studies with adult patients diagnosed with COVID-19 with nose–pharyngeal swab-positive PCR for SARS-CoV-2. Healthy individuals without a history of disease were used as controls. Assessments of microbiota composition were based on stool samples using NGS technology covering V1-V9 regions of 16S rRNA gene. Studies with publicly available raw 16S data for each case and control samples were used (mostly downloaded from online repositories, as Sequence Read Archive, SRA).

## 3. Results

A total of 22 subjects, 8 patients infected with SARS-CoV-2 (five females and three males) and 14 healthy individuals (eleven males and three females), were included in the study. The clinical characteristics of patients are presented in Table 1. The differences in sex, age, and BMI were not statistically significant between the analyzed groups (*p* > 0.05). With regard to supplements, two COVID-19 patients and two healthy controls were taking magnesium supplements, whereas two COVID-19 and one healthy subject were consuming vitamin B complex. The origin of the study group is from one region of the country. There were no subjects on a special diet such as vegan, vegetarian, gluten-free, or high- or low-protein.

**Figure 1:**
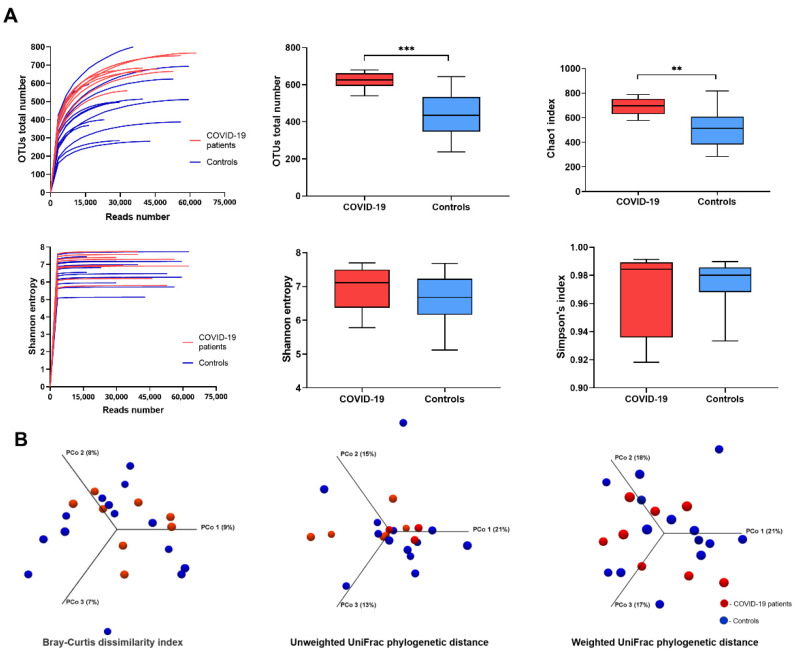
Alpha and beta diversity of the gut microbiota between COVID-19 patients and healthy controls. (**A**) Richness with operational taxonomic units (OTUs) number and within-sample diversity calculated with the Chao1, Shannon entropy, and Simpson index (median, quartile Q1–Q3, min–max). Asterisks denote statistically significant differences given by the Mann–Whitney test (** *p* < 0.01, *** *p* < 0.001) (**B**). Comparison between sample diversities by principal coordinates analysis (PCoA) with a Bray–Curtis dissimilarity matrix and with unweighted and weighted Unifrac distance. Red and blue represent COVID-19 patients and healthy control data, respectively.

In the NGS analysis, the obtained parameters indicate the high quality of the results. The flow cell cluster density was 911 ± 32. The Phred base-calling score GQX ≥ 30 of obtained sequencing data was an average of 78.14%, and the reads passing filter (PF) was 82.72%. Therefore, the mean PF reads were 43,870 ± 11,398 for patients and 33,603 ± 14,978 for controls (Figure 1A). The PF reads of all groups were efficiently classified as bacterial taxa in SILVA v119 97% 16S rRNA database.

In our study, fecal samples of COVID-19 patients demonstrated greater microbial taxonomic diversity compared to the samples collected from healthy individuals based on the deepest available analysis of all regions V1-V9 of the 16S rRNA gene. Our strategy, following the latest data from the literature [20], was aimed at obtaining model results because simplified analyses covering selected regions of this gene (e.g., V3-V4) may cause a quantitative and qualitative disturbance of the identified microbiota taxa. The total OTUs richness for the COVID-19 group (mean OTUs value 615) was statistically significantly higher (*p* < 0.001) compared to control subjects (mean OTUs value 420). Similarly, the Chao1 index was significantly greater (*p* < 0.01) for patients (Figure 1A). Moreover, Shannon entropy and Simpson’s index were calculated to present the alpha diversity of both groups (Figure 1A). Analysis of the beta diversity determined by the Bray–Curtis dissimilarity revealed that the bacterial microbiota of the COVID-19 patient group did not cluster apart from that of a control group. UniFrac distance calculation based on the gut microbiota composition and the phylogeny diversity (sequence distance) both for the absence (unweighted) and for the relative abundance (weighted) of microbiota indicated no significant clustering for both COVID-19 patients and healthy controls (Figure 1B).

**Figure 2:**
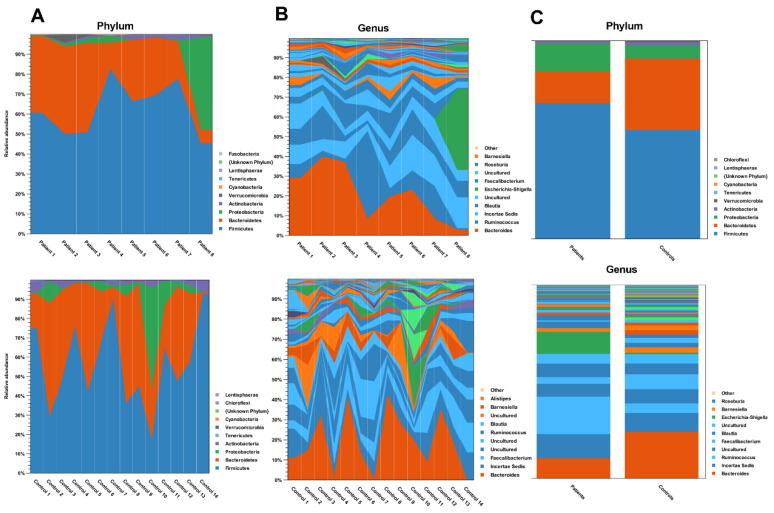
Individual (**A**,**B**) and group (**C**) profiles of gut microbial composition at the phylum and genus level in the COVID-19 patient and healthy control groups. Charts for group profiles show mean relative abundance.

We have analyzed the taxonomic composition of the gut microbiota in both the recovered COVID-19 patients and the control group at the phylum, class, order, family, genus, and species. A total of 17 phyla were detected (Table S1). It has been brought to our attention that the relative abundance of phylum *Verrucomicrobia, Proteobacteria, Firmicutes*, *Bacteroidetes*, and *Actinobacteria* were the dominant bacteria in both groups (Figure 2).

**Figure 3:**
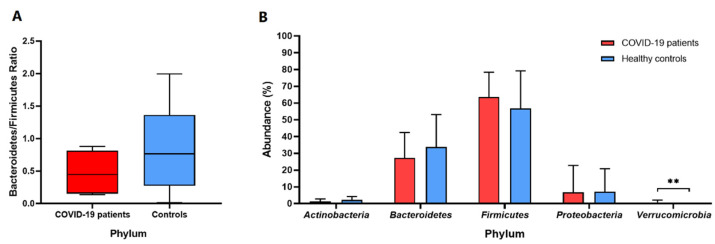
*Bacteroidetes/Firmicutes* ratio (**A**) and the main phylum-level abundance (**B**) in the gut microbiota among COVID-19 patients and healthy controls (median, quartile Q1–Q3, min–max, and mean values with SD, respectively). ** *p* < 0.01 calculated using the non-parametric Mann–Whitney test based on medians.

The mean abundance of the phylum *Verrucomicrobia* was statistically greater (*p* = 0.004) and 30 times higher in SARS-CoV-2 patients compared to healthy controls (0.7378% versus 0.0241%, respectively) (Figure 3B). Nonetheless, the phyla *Proteobacteria, Bacteroidetes,* and *Actinobacteria* were found to have decreased, whereas the *Firmicutes* level was higher in COVID-19 individuals than in healthy controls (Figure 3B). Moreover, the *Bacteroidetes/Firmicutes* ratio was reduced significantly in both COVID-19 patients and healthy controls (a value below 2) (Figure 3A).

**Figure 4:**
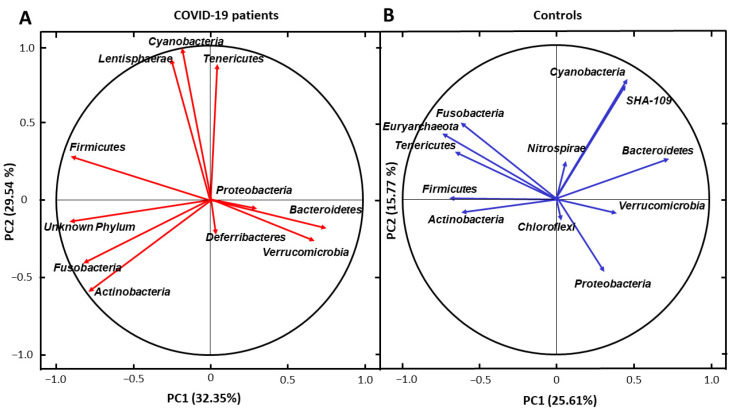
Projection of the microbial composition at phylum level on the two-dimensions biplot for COVID-19 patients (**A**) and healthy controls (**B**). PC1—principal component 1, PC2—principal component 2.

To investigate the correlations between the gut microbiota at the phylum level between COVID-19 patients and healthy controls, principal component analysis (PCA) in two dimensions (PC) was performed. Obtained results demonstrated that in the COVID-19 subjects, *Bacteroidetes* and *Verrucomicrobia* richness were strongly correlated with each other. Additionally, a similar association was observed for *Cyanobacteria* and *Lentisphaerae*. Interestingly, these relationships did not appear in the control group. Moreover, for the healthy subjects, the abundance of *Cyanobacteria* and *SHA-109* were in a strong correlation, which was not found in COVID-19 patients (Figure 4).

**Figure 5:**
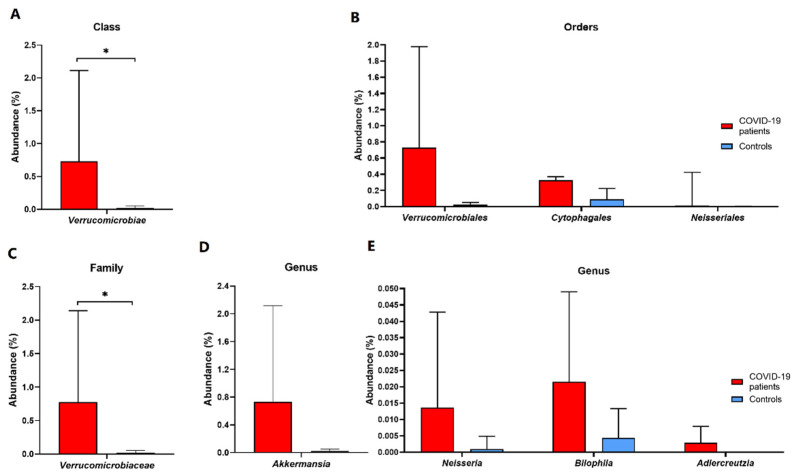
Differences in microbial composition between COVID-19 patients and healthy controls at class (**A**), order (**B**), family (**C**), and genus (**D**,**E**) level (mean values with SD). These data only concern initial statistically significant results calculated using the non-parametric Mann–Whitney test based on medians, and asterisks mark statistical significance obtained after multiple hypothesis testing corrections according to the Benjamini–Hochberg procedure * *p* < 0.05.

Furthermore, a total of 37 classes of microorganisms were detected, and the mean abundance of the *Verrucomicrobiae* class in patients was 30 times higher than that of healthy individuals (0.7316% versus 0.0238%, relatively; Figure 5A; Table S2). Metaprofiling analysis detected a total of 66 orders of microorganisms. An increased abundance of *Verrucomicrobiales, Neisseriales,* and *Cytophagales* was observed over the course of COVID-19 recovery compared to controls (Figure 5B; Table S3). Moreover, a comparative analysis of the microbial community in both groups revealed the presence of a total of 112 families of microorganisms. Seven families, including the *Verrucomicrobiaceae, Neisseriaceae*, *Leuconostocaceae*, *Phyllobacteriaceae, Lachnospiraceae, Clostridiaceae 1,* and *Uncultured bacterium-06,* were initially significantly enriched in COVID-19 patients compared to healthy controls (*p* < 0.05) (Table S4). After the false discovery rate (FDR) correction, statistical significance remained for the family *Verrucomicrobiaceae* (Figure 5C).

**Figure 6:**
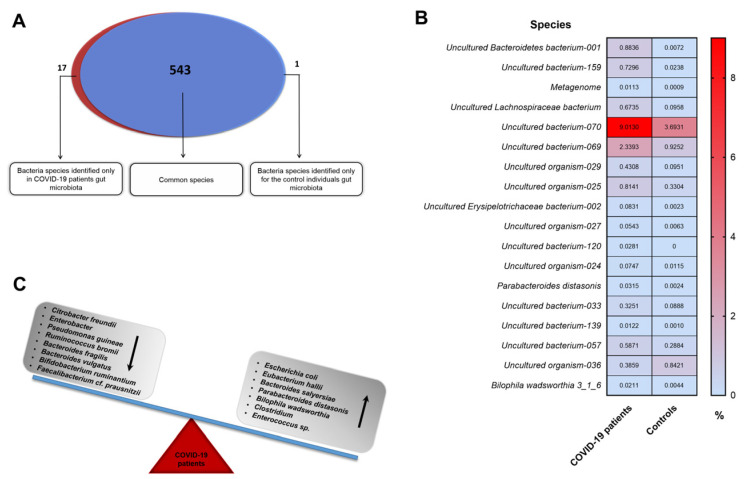
Species abundance distribution and difference in microbial composition between COVID-19 patients and healthy controls. (**A**) Venn diagram for identified in the study gut microbiota species. Red and blue represent COVID-19 patients and healthy controls data, respectively. The intersection shows the species (543) shared with both groups. (**B**) Heatmap of abundance differences in studied group at species level (mean values). These data only concern initial statistically significant results calculated using the non-parametric Mann–Whitney. (**C**) Observed multiple differences in the amount of bacteria abundance (↓—reduction, ↑—increase) between COVID-19 patients’ microbiota compared to healthy controls.

Additionally, using NGS metaprofiling, we have identified a total of 232 genera of microorganisms. The abundance of *Akkermansia, Bilophila, Adlercreutzia,* and *Neisseria* was enriched in COVID-19 patients (*p* < 0.05) (Table S5; Figure 5D,E). Following SARS-CoV-2 recovery, over 50% of bacteria in patients belonged to four genera: *Bacterioides, Incertae Sedis-04, Ruminococcus,* and *Blautia.* Comparatively, when healthy individuals were included, other four genera were dominant: *Bacterioides, Incertae Sedis-04, Faecalibacterium,* and *Ruminococcus*, which constituted 43.8% of the intestinal microbiota. Furthermore, a total of 561 species of microorganisms was detected (Table S6; Figure 6A).

Up to 95.5% of microorganisms that make up the intestinal microbiome of COVID-19 patients have not been cultured in vitro and/or are unknown. Importantly, almost all initial significant differences after the Mann–Whitney test in the quantitative microbiota composition of patients and controls (except for *Parabacteroides distasonis* and *Bilophila wadsworthia*) were observed among the previously uncharacterized and uncultured in vitro microorganisms (average abundance 0.0315% versus 0.0024%, *p* = 0.0289 and 0.0211% versus 0.0044%, *p* = 0.0404, Table S6; Figure 6B). It is worth noting that in patients, of the known species, *Escherichia coli* dominated, which on average accounts for 3.42% of the microbiota composition, whereas in the healthy controls, it encompassed only 0.23%. However, this difference is not statistically significant, similar to the other functionally important bacterial species (Table S6; Figure 6C).

**Figure 7:**
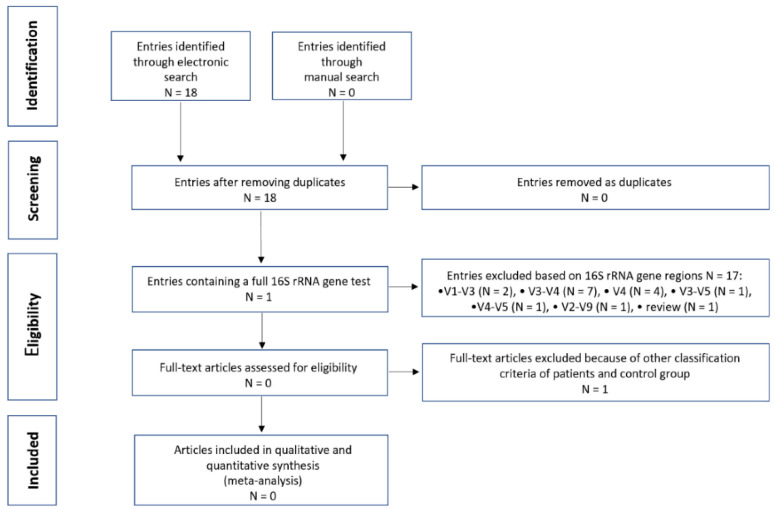
Flowchart of literature search. N—number of entries (research manuscripts). V1–V9—hypervariable regions of the 16S rRNA gene.

Our electronic search identified 18 potentially eligible entries (Figure 7). Among these results, only one was compliant with our methodology (NGS analysis of V1–V9 regions of the 16S rRNA gene) [21]. The rest of the research was excluded using the narrowed-down NGS methodology. Moreover, not all of them were concerned with stool samples but also rectal swabs [22], nasopharyngeal swabs [23], or saliva samples [24]. Finally, after a detailed text analysis of the included manuscript, the determinant of its rejection was the criterion for selecting the studied groups. The subject of this work was not the gut microbiota of COVID-19 patients but the impact of the psychological stress of frontline healthcare workers fighting against COVID-19 on intestinal dysbiosis [21].

## 4. Discussion

In the current study, we compared the gut microbiota compositions of subjects recovered from SARS-CoV-2 and healthy controls. Interestingly, our data have revealed that fecal samples of COVID-19 patients showed statistically greater microbial taxonomic diversity compared to the samples collected from healthy subjects.

The human microbiota consists of numerous microorganisms, including bacteria, viruses, fungi, and archaea, which play an imperative role in the preservation of intestinal microbiota [25]. The healthy human intestinal flora has a greater abundance of phyla *Actinobacteria, Bacteroidetes, Firmicutes,* and *Proteobacteria*; however, the colon often encompasses bacterial families of *Bacteroidaceae, Lachnospiraceae, Prevotellaceae, Riknellaceae,* and *Ruminococcaceae* [26]. Studies have shown that host diet, genetics, mode of birth, age, environmental exposure, and medication consumption contribute to the function and composition of the microbiota [27]. Therefore, its qualitative and quantitative arrangement is dynamic and changes under the influence of the aforementioned factors. Due to its essential role, it is plausible to assume that the microbial community plays a fundamental role in the maintenance of regulatory pathways and the coordination of innate and adaptive responses [28]. It is worth highlighting that about 70–80% of the body’s immune cells are located in the (GI) tract [29]. Notably, studies have reported the importance of crosstalk between the gut–lung axis and the intestinal microbiota–mucosal immune system [30].

Moreover, growing evidence suggests that lung immunity can be affected by alterations in the taxonomic composition and reduced diversity of the microbiota. In the literature, dysbiosis is defined as an imbalance in the gut microbiota composition or a change in the bacterial distribution or metabolic activities within the bowel [31]. These alterations have been proven to disrupt the intestinal mucosal barrier integrity, perpetuating systemic inflammation. It has been reported that increased gut permeability may lead to bacterial translocation from distant organs into the intestines via the lymphatic and circulatory systems [32]. This mechanism is believed to be associated with the pathogenesis of sepsis and acute respiratory distress syndrome [33]. An interesting study presented direct evidence that SARS-CoV-2 is translocated from the pulmonary system into the enterocytes, such that it influences the microbial community composition [34]. This disruption of the corresponding intestinal microbiome explains why over 60% of patients infected with SARS-CoV-2 present symptoms such as nausea, vomiting, and diarrhea [35].

Previous reports have highlighted the importance of the gut microbiota–lung axis and related respiratory infections [36]. Consistently, an increased abundance of opportunistic pathogens, including phylum *Actinomyces, Erysipelatoclostridium, Streptococcus, Rothia,* and *Veillonella,* in COVID-19 patients was reported [37]. In our study, the fecal metagenomic analysis revealed a statistically greater abundance of the genera *Akkermansia, Bilophila, Adlercreutzia,* and *Neisseria,* indicating that the dysbiosis persisted during SARS-CoV-2 infection and was not fully restored after 3 three months’ recovery. Moreover, the depletion of potentially beneficial bacterial families *Ruminococcaceae* and *Lachnospiraceae* was noted, which is in line with other studies [37]. Additionally, a previous report revealed an increased abundance of opportunistic bacteria *Neisseria* and *Rothia* in oral and intestinal microbiota during the first few days after COVID-19 symptom onset [38]. Our metaprofiling analysis has shown a statistically significant increase in *Neisseriaceae,* which appear vastly in the upper airways. These correlations may imply the bidirectional relationship between lungs and gut microbiota. It is worth noting that the fecal microbiota of subjects infected with SARS-CoV-2 was characterized by the reduction of beneficial bacteria and enriched by potentially harmful commensals (*Bacteroides nordii* and *Clostridium hathewayi)*. Our findings are consistent with these results; however, the increased abundance of *Bacteroidetes* and *Firmicutes* did not reach statistical significance. More importantly, the severity of COVID-19 infection has been vastly related to gut dysbiosis [39]. In our study, the class *Clostridia* in recovered patients was significantly more abundant than in the control group. In line with that, a pilot study reported that the baseline abundance of *Clostridium hathewayi, Clostridium ramosum,* and *Coprobacillus* positively correlates with disease severity, whereas the reduced abundance of *Faecalibacterium prausnitzii* inversely correlates with SARS-CoV-2 infectivity [37]. Notably, increased levels of blood markers and inflammatory cytokines such as C-reactive protein, gamma-glutamyltransferase, aspartate aminotransferase, lactate dehydrogenase, IL-10, and TNF-alfa have been reported. Interestingly, in a cohort study, stool samples of COVID-19 patients revealed a lower relative abundance of *Eubacterium rectale, Faecalibacterium prausnitzii,* and multiple *Bifidobacterium* species [40]. Our findings are consistent with a previous study conducted in SARS-CoV-2 patients, in which a greater abundance of *Akkermansia muciniphila* and *Bacteroides dorei* was noted [40]. Moreover, these microorganisms positively correlated with C-X-C motif ligand 8 (CXCL8), interleukin-6 (IL-6), and IL-1β [40]. In a recent cross-sectional study, authors reported significantly greater levels also of IL-6 [41]. Moreover, subjects with COVID-19 had an increased abundance of potentially harmful pathogens including *Actinomyces, Veillonella, Streptococcus,* and *Rothia.* It is worth emphasizing that studies have shown that the prevalence of *Bacteroidetes stercoris, Parabacteroides merdae, Alistipes onderdonkii,* and *Lachnospiraceae bacterium* correlates with decreased COVID-19 infectivity [42]. In light of reports, the depletion of *B. adolescentis, F. prausnitzii, E. rectale, R. (Blautia) obeum,* and *D. formicigenerans* markedly increases microbial-induced immune dysregulation [40]. As such, NF-κB suppression by *B. adolescentis* has been proven to reduce the expression of pro-inflammatory cytokines. Moreover, in patients with severe COVID-19, markedly elevated levels of inflammatory markers such as IL-1, IL-6, and TNF-alfa have been detected [43]. Furthermore, clinical observations point out that subjects infected with SARS-CoV-2 have a significantly reduced abundance of short-chain-fatty-acid-producing bacteria (short-chain fatty acids—SCFAs), including *Eubacterium hallii, Fusicatenibacter,* and *Faecalibacterium praustnitzii* [41,44]. These findings are in line with our study, in which fecal samples collected from the recovered COVID-19 subjects showed a decreased abundance of the butyrate-producing bacteria of the family *Lachnospiraceae* and *Ruminococcaceae.* Emerging data revealed that lactate/sugar-utilizing bacteria including *Anaerostipes* species along with *Eubacterium hallii* can convert acetate and lactate into butyrate [44]. Similarly, our COVID-19-recovered patients had reduced fecal abundance of *Anaerostipes* species compared to healthy controls. Furthermore, apart from the aforementioned microorganisms, phylum *Actinobacteria,* including *Bifidobacterium* species, yields SCFA lactate and acetate during carbohydrate fermentation [45]. Moreover, our metaprofiling analysis revealed an increased abundance of phylum *Verrucomicrobia* and the genus *Akkermansia*. Apart from acetate and propionate production, this microorganism stimulates mucus degradation [46]. Some studies have also reported the importance of *Akkermansia muciniphila* in intestinal wound healing, induction of the immune response, and production of cytokines, IL-10, and SCFAs [47,48]. These molecules have been reported to regulate the intestinal immune system and mucosal permeability by preventing circulation and translocation of enteric endotoxins and their metabolites. Studies have shown that butyrate, one of the SCFAs in the intestinal epithelial cells, increases the formation and release of IL-18, which is crucial in maintaining homeostasis during inflammatory processes [49,50]. Mounting evidence suggests that at the molecular level, butyrate enhances the intestinal barrier by favoring the redistribution of tight-junction proteins occludin and zonula occludent-1 (ZO-1) [51]. Moreover, this key molecule increases oxygen formation and, therefore, stabilizes the hypoxia-inducible transcription factor (HIF) engaged in antimicrobial peptide production [52]. Furthermore, data have shown that consuming a fiber-rich diet increases the relative abundance of families *Rikenellaceae, Porphyromonadaceae,* and *Lachnospiracea.* Additionally, recent data have revealed that in subjects infected with SARS-CoV-2, proline and arginine metabolism was upregulated, indicating intestinal barrier dysfunction. These amino acids play a fundamental role in numerous chemical processes, such as the urea cycle, protein synthesis, and nitric oxide production [53]. Interestingly, it was also reported that one of the mechanisms of the pathogenesis of gut dysbiosis is linked to angiotensin-converting enzyme 2 expression. Recent investigations in the murine colon have shown that *Bacteroides* species such as *Bacteroides massiliensis, Bacteroides thetaiotaomicron, Bacteroides dorei,* and *Bacteroides ovatus* downregulate ACE2 receptor expression, which inversely correlates with COVID-19 severity [37]. Moreover, receptor downregulation markedly reduces antimicrobial peptide secretion as well as enteric tryptophan (Try) absorption [54]. A recent report demonstrated that plasma levels of this essential amino acid have been significantly reduced in ACE2-depleted mice [55]. Tryptophan is actively absorbed from the intestines and serves as the main precursor for the synthesis of tryptamine and serotonin neurotransmitters [56]. Emerging evidence showed that its deficiency plays a key role in the development of anxiety, depression, fatigue, muscle weakness, sleep, and mood disturbances. Moreover, Ninomiya et al. revealed that low levels of tryptophan underlie the pathogenesis of muscle atrophy [57]. Furthermore, this propensity may imply that Try depletion causes weakening of the diaphragm muscle and, therefore, dyspnea.

Furthermore, our study has revealed an enrichment of pathobionts in the gut microbiota of COVID-19 subjects. These include *E. coli*, which is known for its pro-inflammatory properties, and *B. wadsworthia,* which utilizes the activity of the sulfated amino acid taurine in the production of hydrogen sulfide and the rapid catalase reaction, thereby contributing to the pathogenesis of various diseases. Additionally, an increased abundance of other bacteria with pathogenic potentials, such as *Parabacteroides distasonis* and *Enterococcus* sp. *CGLBL186*, were noted. Moreover, our metaprofiling analysis showed an increased level of *E. hallii*, which contributes to intestinal propionate formation and glucose utilization [58]. On the other hand, the depletion of toxin-producing pathogenic *Citrobacter freundii* was reported. Similarly, a reduced abundance of *B. fragilis* was noted, which may be responsible for the development of endocarditis, meningitis, septic arthritis, and osteomyelitis. Interestingly, it has been brought to our attention that the beneficial commensals, i.e., *Bifidobacterium ruminantium* and *Faecalibacterium prausnitzii,* were decreased in the COVID-19 group (Figure 6C). It is plausible to assume that a reduced *Firmicutes/Bacteroidetes* ratio in SARS-CoV-2 subjects may indicate ongoing inflammatory processes and may be used as an inflammatory marker [59] (Figure 3).

The research we have carried out has several important strengths. Our metaprofiling analysis, including all hypervariable regions (V1–V9) of the 16S rRNA marker gene, gives a complete, deep picture of the microbial community composition in samples collected from both studied groups. In contrast to the commonly used 16S rRNA taxonomic marker narrowed down to the V3–V4 regions, our research allowed us to identify all the possible families, genera, and species of bacteria with equal affinity. Earlier studies have shown that analyses based on all variable regions of the 16S rRNA gene can deliver much more data than the selected regions of this gene [20]. Moreover, it is worth emphasizing that our studied cohorts are homogeneous and chosen according to strict criteria (Table 1). The patient’s group included only subjects with a mild course of COVID-19 disease, without antibiotic and probiotic intake in the last six months. At the same time, the collected controls of healthy individuals were checked during internal control analysis to confirm group homogeneity. For this purpose, the control group we divided into two subgroups, and the metadata of microbiota composition at all taxonomic levels we compared. No significant differences were found.

It is acknowledged that this pilot study has certain limitations. Firstly, the small sample size of enrolled individuals, particularly in the SARS-CoV-2 recovered patients. Thus, to verify our findings, we conducted integrative research with other similar studies applying the same methodology pipeline in the comparative analysis of COVID-19 patients versus healthy controls microbiota. Surprisingly, we found only one manuscript (from two months ago) that included a full 16S rRNA gene analysis but, nevertheless, in a different clinical aspect [21] which confirms our approach and results are pioneering, and the meta-analysis was not able to be performed. Therefore, studies including a larger population are required for further validation.

Secondly, stool collection was taken at only a one-time point at 3 months following the COVID-19 symptom onset. Thus, future exploratory studies should ideally collect fecal material at SARS-CoV-2 onset and during the disease course, followed by post-infection recovery. Thirdly, presented microbiome alterations not only may be caused by COVID-19 but also may be associated with co-infections with different pathogens or other diseases.

## 5. Conclusions

In conclusion, our findings highlight the importance of microbial community in the course of SARS-CoV-2 infection. Moreover, the gut microbiota has the propensity to drive inflammatory responses by influencing the release of pro- and anti-inflammatory cytokines, including antiviral responses at distal mucosal sites, including the lungs. Additionally, current research provides strong evidence for the gut–lung crosstalk in the pathogenesis of COVID-19 and possible detection of the viral particles in the feces following recovery. It is plausible to assume that the extent of intestinal dysbiosis and increased gut permeability may contribute to COVID-19 infection severity. Considering the data published to date on SARS-CoV-2, we may conclude that adjunctive therapies focusing on a correction of intestinal dysbiosis may be a crucial therapeutic approach, as previously shown for other disorders, such as inflammatory bowel diseases [19,48]. This long-range protection conferred by the microbiota has been attributed to metabolites secreted by specific bacterial species. Hence, metabolomics may also be the direction of further research into the influence of the microbiota on COVID-19 infection.

Considering the gut microbiota’s ability to disturb the immune response, our findings suggest the importance of the enteric microbiota in the course of SARS-CoV-2 infection. Mounting evidence reveals that increased intestinal permeability and enhanced release of bacterial toxins and metabolites contribute to the severity of COVID-19 infection. Moreover, this pilot study shows that the composition of the microbial community may not be fully restored in individuals with SARS-CoV-2 following a 3-month recovery.

## Figures and Tables

**Table 1:** Demographic and clinical data of the study cohort.

Parameter	Total *n* = 22	COVID-19 Patients *n* = 8	Healthy Controls *n* = 14	*p*-Value
Gender				0.0541
Female, *n* (%)	8 (36.36)	5 (62.5)	3 (21.43)	
Male, *n* (%)	14 (63.64)	3 (37.5)	11 (78.57)	
Age, (years) mean ± SD (min.–max.)	36.23 ± 12.51 (20–59)	34.88 ± 11.21 (20–59)	37 ± 110.91 (22–57)	0.6680
BMI (kg/m^2^), mean ± SD (min.–max.)	25,64 ± 3.525 (20.90–32.87)	24.86 ± 3.784 (20.90–32.27)	26,09 ± 3.431 (20.94–32.87)	0.4468
Smoker, *n* (%)	0 (0.00)	0 (0.00)	0 (0.00)	NA
Probiotics taking, *n* (%)	0 (0.00)	0 (0.00)	0 (0.00)	NA
Supplements taking, *n* (%)	7 (31.82)	4 (50.00)	3 (21.43)	0.1663
Antibiotics, last 6 months, *n* (%)	0 (0.00)	0 (0.00)	0 (0.00)	NA
COVID-19 symptoms				
Cough, *n* (%)	3 (13.64)	3 (37.50)	-	NA
Fever, *n* (%)	5 (22.73)	5 (62.50)	-	NA
Dyspnea, *n* (%)	1 (4.55)	1 (12.50)	-	NA
Headache, *n* (%)	3 (13.64)	3 (37.50)	-	NA
Sinus pain	2 (9.09)	2 (25.00)	-	NA
Myalgia, *n* (%)	3 (13.64)	3 (37.50)	-	NA
Chest pain, *n* (%)	2 (9.09)	2 (25.00)	-	NA
Fatigue, *n* (%)	6 (27.27)	6 (75.00)	-	NA
Taste loss, *n* (%)	5 (22.73)	5 (62.50)	-	NA
Sense of smell loss, *n* (%)	4 (18.18)	4 (50.00)	-	NA
Symptom duration (days), mean ± SD (min.–max.)	-	7.125 ± 3.40 (4–14)	-	NA

SD—standard deviation; NA—not analyzed; *p*-values calculated by the non-parametric Mann–Whitney test (age, BMI) and Chi-square test (the remaining).

## Data Availability

The datasets generated for this study were deposited in the Short Read Archive database (SRA) (https://www.ncbi.nlm.nih.gov/bioproject/PRJNA817437, accessed on 11 January 2023).

## Supplementary Materials

The following are available online at https://www.mdpi.com/article/10.3390/biomedicines11020367/s1, Table S1. The percentage abundance of the intestinal microbiota in the study group of COVID-19 patients and healthy controls at the phylum level, Table S2. The percentage abundance of the intestinal microbiota in the study group of COVID-19 patients and healthy controls at the class level, Table S3. The percentage abundance of the intestinal microbiota in the study group of COVID-19 patients and healthy controls at the order level, Table S4. The percentage abundance of the intestinal microbiota in the study group of COVID-19 patients and healthy controls at the family level, Table S5. The percentage abundance of the intestinal microbiota in the study group of COVID-19 patients and healthy controls at the genus level, Table S6. The percentage abundance of the intestinal microbiota in the study group of COVID-19 patients and healthy controls at the species level.
