# Supplementary material for: Alterations in Gut Microbiota Composition in Patients with COVID-19: A Pilot Study of Whole Hypervariable 16S rRNA Gene Sequencing
Source: Biomedicines. 2023 Jan 27;11(2):367. doi: 10.3390/biomedicines11020367 (PMC9953267; doi:10.3390/biomedicines11020367)
Supplement: Supplementary file 1 [file biomedicines-11-00367-s001.zip › biomedicines-2108463-supplementary.pdf]

Supplementary Materials

Alterations in Gut Microbiota Composition in Patients with COVID-19: A Pilot Study of Whole Hypervariable 16S rRNA Gene Sequencing

Dorota Mańkowska-Wierzbicka, Joanna Zuraszek, Adrianna Wierzbicka, Marcin Gabryel, Dagmara Mahadea, Alina Baturo, Oliwia Zakerska-Banaszak, Ryszard Slomski, Marzena Skrzypczak-Zielinska and Agnieszka Dobrowolska

Table S1. The percentage abundance of the intestinal microbiota in the study group of COVID-19 patients and healthy controls at the phylum level.

Phylum	COVID-19 patients, *n* = 8					Controls, *n* = 14					*p*-value	*p*-adj.
	**Mean**	**Median**	**SD**	**Min.**	**Max.**	**Mean**	**Median**	**SD**	**Min.**	**Max.**		
Verrucomicrobia	0.7378	0.1971	10.3796	0.0496	40.1029	0.0241	0.0160	0.0290	0.0000	0.0923	**0.0004**	**0.0028**
{Unknown Phylum} Bacteria	0.0024	0.0000	0.0055	0.0000	0.0157	0.0000	0.0000	0.0000	0.0000	0.0000	0.0993	0.2754
Deferribacteres	0.0006	0.0000	0.0017	0.0000	0.0049	0.0000	0.0000	0.0000	0.0000	0.0000	0.1354	0.6189
Euryarchaeota	0.0000	0.0000	0.0000	0.0000	0.0000	0.0021	0.0000	0.0067	0.0000	0.0251	0.2461	0.6189
Proteobacteria	60.7832	0.8648	160.0545	0.0883	460.4070	70.0678	10.5173	130.7392	0.0154	520.4754	0.2573	0.6189
Bacteroidetes	270.3214	30.2859	150.1309	60.7571	440.7306	330.7927	30.8585	190.3539	0.8767	580.7455	0.2719	0.6189
Firmicutes	630.7174	630.6255	140.6559	450.4394	840.0068	560.7847	530.7607	220.4752	170.6835	920.7791	0.2944	0.6189
Actinobacteria	10.3297	0.9977	10.4225	0.0716	40.3535	20.2085	10.5965	20.0419	0.1847	60.3288	0.4469	0.6189
Chloroflexi	0.0000	0.0000	0.0000	0.0000	0.0000	0.0050	0.0000	0.0186	0.0000	0.0697	0.4469	0.6189
Nitrospirae	0.0000	0.0000	0.0000	0.0000	0.0000	0.0005	0.0000	0.0020	0.0000	0.0076	0.4469	0.6189
SHA-109	0.0000	0.0000	0.0000	0.0000	0.0000	0.0003	0.0000	0.0011	0.0000	0.0043	0.6253	0.6189
Cyanobacteria	0.0633	0.0000	0.1357	0.0000	0.3812	0.0142	0.0000	0.0458	0.0000	0.1711	0.6605	0.6189
Lentisphaerae	0.0121	0.0000	0.0328	0.0000	0.0931	0.0019	0.0000	0.0050	0.0000	0.0153	0.6849	0.6539
Fusobacteria	0.0007	0.0000	0.0013	0.0000	0.0032	0.0014	0.0000	0.0038	0.0000	0.0125	0.7523	0.7678
Tenericutes	0.0324	0.0000	0.0598	0.0000	0.1516	0.0968	0.0000	0.1891	0.0000	0.4700	0.8924	0.8742
Spirochaetae	0.0000	0.0000	0.0000	0.0000	0.0000	0.0000	0.0000	0.0000	0.0000	0.0000	NA	NA
Synergistetes	0.0000	0.0000	0.0000	0.0000	0.0000	0.0000	0.0000	0.0000	0.0000	0.0000	NA	NA

SD—standard deviation; NA—not available; *p*-value—calculated with the non-parametric Mann–Whitney test; *p*-adj.—*p* values adjusted with Benjamini–Hochberg false-discovery rate (FDR) for multiple comparisons; red font—statistically significant results ($p < 0.05$).

Table S2. The percentage abundance of the intestinal microbiota in the study group of COVID-19 patients and healthy controls at the class level.

Class	COVID-19 patients, $n = 8$					Controls, $n = 14$					*p*-value	*p*-adj.
	**Mean**	**Median**	**SD**	**Min.**	**Max.**	**Mean**	**Median**	**SD**	**Min.**	**Max.**		
Verrucomicrobiae	0.7316	0.1971	10.3831	0.0251	40.1029	0.0238	0.0160	0.0286	0.0000	0.0923	**0.0017**	**0.0126**
{Unknown Phylum} Bacteria	0.0014	0.0000	0.0029	0.0000	0.0078	0.0000	0.0000	0.0000	0.0000	0.0000	0.0648	0.3997
Cytophagia	0.1470	0.1841	0.0932	0.0143	0.2621	0.0888	0.0314	0.1356	0.0000	0.3998	0.1083	0.5409
Erysipelotrichia	10.1282	10.1296	0.3805	0.2911	10.4662	10.7849	0.5518	20.8593	0.0978	90.8891	0.1423	0.5409
VC20.1 Bac22	0.3792	0.2049	0.4976	0.0107	10.5312	0.1956	0.0473	0.2737	0.0000	0.8772	0.1820	0.5409
Deltaproteobacteria	0.0271	0.0211	0.0284	0.0000	0.0838	0.0194	0.0019	0.0384	0.0000	0.1412	0.1840	0.5409
Epsilonproteobacteria	0.0000	0.0000	0.0000	0.0000	0.0000	0.2294	0.0000	0.8275	0.0000	30.1036	0.1885	0.5409
Negativicutes	0.3318	0.3440	0.1640	0.0081	0.5639	0.2598	0.1076	0.3600	0.0000	0.9634	0.2066	0.5409
Deferribacteres	0.0006	0.0000	0.0017	0.0000	0.0049	0.0000	0.0000	0.0000	0.0000	0.0000	0.2193	0.5409
Flavobacteriia	0.0004	0.0000	0.0012	0.0000	0.0035	0.0000	0.0000	0.0000	0.0000	0.0000	0.2193	0.5409
{Unknown Class} Bacteroidetes	0.0014	0.0000	0.0039	0.0000	0.0111	0.0000	0.0000	0.0000	0.0000	0.0000	0.2193	0.5409
BD2-02	0.0000	0.0000	0.0000	0.0000	0.0000	0.0006	0.0000	0.0014	0.0000	0.0040	0.3050	0.6373
Bacilli	0.6528	0.1830	10.4033	0.0175	40.1103	0.3046	0.0662	0.6797	0.0077	20.5801	0.3223	0.6373
{Unknown Class} Firmicutes	0.0009	0.0000	0.0016	0.0000	0.0036	0.0003	0.0000	0.0011	0.0000	0.0043	0.3311	0.6373
Betaproteobacteria	0.2517	0.1369	0.3425	0.0072	10.0749	0.9576	0.3143	10.4682	0.0077	50.2236	0.3936	0.6373
Bacteroidia	260.7933	290.6565	140.9658	60.4034	440.6102	330.5067	30.5288	190.3503	0.6114	570.8684	0.4325	0.6373
Clostridia	610.6032	610.4062	150.2342	40.1500	820.3551	540.2842	520.3424	20.8636	160.8490	910.5987	0.4325	0.6373
Alphaproteobacteria	0.8228	0.2411	10.0968	0.0000	30.0142	10.1887	0.0134	20.1792	0.0000	60.3184	0.4483	0.6373
Anaerolineae	0.0000	0.0000	0.0000	0.0000	0.0000	0.0050	0.0000	0.0186	0.0000	0.0697	0.5083	0.6373
Methanobacteria	0.0000	0.0000	0.0000	0.0000	0.0000	0.0018	0.0000	0.0067	0.0000	0.0251	0.5083	0.6373
Nitrospira	0.0000	0.0000	0.0000	0.0000	0.0000	0.0005	0.0000	0.0020	0.0000	0.0076	0.5083	0.6373
Sphingobacteriia	0.0000	0.0000	0.0000	0.0000	0.0000	0.0011	0.0000	0.0041	0.0000	0.0152	0.5083	0.6373
Thermoplasmata	0.0000	0.0000	0.0000	0.0000	0.0000	0.0003	0.0000	0.0011	0.0000	0.0040	0.5083	0.6373
Uncultured bacterium	0.0000	0.0000	0.0000	0.0000	0.0000	0.0003	0.0000	0.0011	0.0000	0.0043	0.5083	0.6373
Melainabacteria	0.0633	0.0000	0.1357	0.0000	0.3812	0.0142	0.0000	0.0458	0.0000	0.1711	0.5097	0.6373
Actinobacteria	0.8440	0.2839	10.2237	0.0143	30.2455	10.4192	0.7035	10.5873	0.0038	40.8331	0.5167	0.6373
Coriobacteriia	0.4857	0.3626	0.4305	0.0573	10.1080	0.7893	0.3324	0.9334	0.0000	30.1898	0.5618	0.6671
Lentisphaeria	0.0121	0.0000	0.0328	0.0000	0.0931	0.0019	0.0000	0.0050	0.0000	0.0153	0.5770	0.6671
Opitutae	0.0063	0.0000	0.0177	0.0000	0.0501	0.0003	0.0000	0.0010	0.0000	0.0038	0.6816	0.7642
Fusobacteriia	0.0007	0.0000	0.0013	0.0000	0.0032	0.0014	0.0000	0.0038	0.0000	0.0125	0.7226	0.7864
Gammaproteobacteria	50.6816	0.0175	150.7006	0.0000	440.5328	40.6727	0.1321	130.6763	0.0000	50.6354	0.7574	0.8007
Mollicutes	0.0324	0.0000	0.0598	0.0000	0.1516	0.0968	0.0000	0.1891	0.0000	0.4700	0.8742	0.8985
OPB54	0.0006	0.0000	0.0017	0.0000	0.0049	0.1509	0.0000	0.5635	0.0000	20.1086	0.9544	0.9544
Chloroplast	0.0000	0.0000	0.0000	0.0000	0.0000	0.0000	0.0000	0.0000	0.0000	0.0000	NA	NA
Spirochaetes	0.0000	0.0000	0.0000	0.0000	0.0000	0.0000	0.0000	0.0000	0.0000	0.0000	NA	NA
Synergistia	0.0000	0.0000	0.0000	0.0000	0.0000	0.0000	0.0000	0.0000	0.0000	0.0000	NA	NA
VadinHA17	0.0000	0.0000	0.0000	0.0000	0.0000	0.0000	0.0000	0.0000	0.0000	0.0000	NA	NA

SD—standard deviation; NA—not available; *p*-value—calculated with the non-parametric Mann–Whitney test; *p*-adj.—*p* values adjusted with Benjamini–Hochberg FDR for multiple comparisons; red font—statistically significant results ($p < 0.05$).

Table S3. The percentage abundance of the intestinal microbiota in the study group of COVID-19 patients and healthy controls at the order level.

Order	COVID-19 patients, *n* = 8					Controls, *n* = 14					*p*-value	*p*- adj.
	**Mean**	**Median**	**SD**	**Min.**	**Max.**	**Mean**	**Median**	**SD**	**Min.**	**Max.**		
Verrucomicrobiales	0.7296	0.1971	10.3843	0.0092	40.1029	0.0238	0.0160	0.0286	0.0000	0.0923	**0.0027**	0.1356
Cytophagales	0.3251	0.1953	0.4565	0.0364	10.4389	0.0888	0.0314	0.1356	0.0000	0.3998	**0.0314**	0.5809
Neisseriales	0.0122	0.0018	0.0296	0.0000	0.0854	0.0010	0.0000	0.0039	0.0000	0.0144	**0.0365**	0.5809
Propionibacteriales	0.0311	0.0035	0.0445	0.0000	0.1109	0.0019	0.0000	0.0058	0.0000	0.0217	0.0504	0.5809
B38	0.0107	0.0000	0.0202	0.0000	0.0494	0.0000	0.0000	0.0000	0.0000	0.0000	0.0648	0.5809
Uncultured bacterium	0.4477	0.3193	0.4770	0.0652	10.5312	0.1956	0.0473	0.2737	0.0000	0.8772	0.0697	0.5809
Pasteurellales	0.0000	0.0000	0.0000	0.0000	0.0000	0.0028	0.0000	0.0052	0.0000	0.0158	0.1159	0.6450
Burkholderiales	0.2087	0.0886	0.3587	0.0072	10.0749	0.9555	0.3143	10.4697	0.0077	50.2236	0.1618	0.6450
Campylobacterales	0.0000	0.0000	0.0000	0.0000	0.0000	0.2294	0.0000	0.8275	0.0000	30.1036	0.1885	0.6450
Pseudomonadales	0.0000	0.0000	0.0000	0.0000	0.0000	0.8145	0.0000	30.0433	0.0000	110.3882	0.1885	0.6450
Rhizobiales	0.0225	0.0032	0.0347	0.0000	0.0976	0.0019	0.0000	0.0033	0.0000	0.0077	0.1914	0.6450
Actinomycetales	0.0029	0.0000	0.0083	0.0000	0.0235	0.0000	0.0000	0.0000	0.0000	0.0000	0.2193	0.6450
Corynebacteriales	0.0040	0.0000	0.0113	0.0000	0.0319	0.0000	0.0000	0.0000	0.0000	0.0000	0.2193	0.6450
Deferribacterales	0.0006	0.0000	0.0017	0.0000	0.0049	0.0000	0.0000	0.0000	0.0000	0.0000	0.2193	0.6450
Flavobacteriales	0.0004	0.0000	0.0012	0.0000	0.0035	0.0000	0.0000	0.0000	0.0000	0.0000	0.2193	0.6450
{Unknown Class} Bacteroidetes	0.0014	0.0000	0.0039	0.0000	0.0111	0.0000	0.0000	0.0000	0.0000	0.0000	0.2193	0.6450
{Unknown Phylum} Bacteria	0.0010	0.0000	0.0028	0.0000	0.0078	0.0000	0.0000	0.0000	0.0000	0.0000	0.2193	0.6450
Desulfovibrionales	0.0267	0.0211	0.0288	0.0000	0.0838	0.0194	0.0019	0.0384	0.0000	0.1412	0.2732	0.6517
Kordiimonadales	0.0330	0.0000	0.0918	0.0000	0.2602	0.0096	0.0000	0.0358	0.0000	0.1338	0.2774	0.6517
Thermoanaerobacterales	0.0149	0.0000	0.0411	0.0000	0.1165	0.0063	0.0000	0.0234	0.0000	0.0876	0.2774	0.6517
Uncultured sediment bacterium	0.0000	0.0000	0.0000	0.0000	0.0000	0.0006	0.0000	0.0014	0.0000	0.0040	0.3050	0.6517
Selenomonadales	0.2906	0.3095	0.1940	0.0081	0.5639	0.2598	0.1076	0.3600	0.0000	0.9634	0.3567	0.6517
Clostridiales	620.7777	610.4062	170.3990	40.1500	910.3483	540.2660	520.3099	20.8667	160.8490	910.5987	0.3568	0.6517
Erysipelotrichales	10.0187	10.1259	0.5006	0.2125	10.4662	10.7849	0.5518	20.8593	0.0978	90.8891	0.3568	0.6517
Bacteroidales	250.9044	290.6565	160.0774	50.8086	440.6102	330.5067	30.5288	190.3503	0.6114	570.8684	0.4325	0.6517
Lactobacillales	0.6005	0.0802	10.3631	0.0046	30.9579	0.1044	0.0490	0.1679	0.0038	0.6424	0.4325	0.6517
Rhodospirillales	0.3963	0.0892	0.5789	0.0000	10.4108	10.1767	0.0058	20.1791	0.0000	60.3184	0.4418	0.6517
Halanaerobiales	0.0737	0.0025	0.1943	0.0000	0.5542	0.0120	0.0000	0.0258	0.0000	0.0815	0.4519	0.6517
Bacillales	0.0191	0.0000	0.0539	0.0000	0.1525	0.2002	0.0000	0.6850	0.0000	20.5763	0.4857	0.6517
Micrococcales	0.0167	0.0040	0.0328	0.0000	0.0952	0.0283	0.0000	0.0751	0.0000	0.2780	0.5023	0.6517
Anaerolineales	0.0000	0.0000	0.0000	0.0000	0.0000	0.0050	0.0000	0.0186	0.0000	0.0697	0.5083	0.6517
Hydrogenophilales	0.0000	0.0000	0.0000	0.0000	0.0000	0.0011	0.0000	0.0041	0.0000	0.0153	0.5083	0.6517
Methanobacteriales	0.0000	0.0000	0.0000	0.0000	0.0000	0.0018	0.0000	0.0067	0.0000	0.0251	0.5083	0.6517
Nitrospirales	0.0000	0.0000	0.0000	0.0000	0.0000	0.0005	0.0000	0.0020	0.0000	0.0076	0.5083	0.6517
Puniceicoccales	0.0000	0.0000	0.0000	0.0000	0.0000	0.0003	0.0000	0.0010	0.0000	0.0038	0.5083	0.6517
Sphingobacteriales	0.0000	0.0000	0.0000	0.0000	0.0000	0.0011	0.0000	0.0041	0.0000	0.0152	0.5083	0.6517
Sphingomonadales	0.0000	0.0000	0.0000	0.0000	0.0000	0.0005	0.0000	0.0020	0.0000	0.0076	0.5083	0.6517
Thermoplasmatales	0.0000	0.0000	0.0000	0.0000	0.0000	0.0003	0.0000	0.0011	0.0000	0.0040	0.5083	0.6517
Uncultured bacterium 05	0.0000	0.0000	0.0000	0.0000	0.0000	0.0003	0.0000	0.0011	0.0000	0.0043	0.5083	0.6517
Coriobacteriales	0.5005	0.3626	0.4154	0.0736	10.1080	0.7893	0.3324	0.9334	0.0000	30.1898	0.6573	0.8216
RF9	0.0115	0.0000	0.0303	0.0000	0.0864	0.0901	0.0000	0.1791	0.0000	0.4700	0.6956	0.8402
Bifidobacteriales	0.8290	0.2393	10.1963	0.0212	30.1712	10.3891	0.6926	10.5926	0.0038	40.8331	0.7074	0.8402
Fusobacteriales	0.0007	0.0000	0.0013	0.0000	0.0032	0.0014	0.0000	0.0038	0.0000	0.0125	0.7226	0.8402
{Unknown Class} Firmicutes	0.0004	0.0000	0.0011	0.0000	0.0032	0.0003	0.0000	0.0011	0.0000	0.0043	0.7844	0.8914
Enterobacteriales	50.6620	0.0123	150.6936	0.0000	440.4962	30.8389	0.0546	130.4746	0.0000	50.6274	0.8623	0.9190
Victivallales	0.0004	0.0000	0.0012	0.0000	0.0035	0.0019	0.0000	0.0050	0.0000	0.0153	0.8638	0.9190
Xanthomonadales	0.0009	0.0000	0.0025	0.0000	0.0071	0.0165	0.0000	0.0559	0.0000	0.2097	0.8638	0.9190
Gastranaerophilales	0.0156	0.0000	0.0442	0.0000	0.1251	0.0142	0.0000	0.0458	0.0000	0.1711	0.9544	0.9544
NB10n	0.0082	0.0000	0.0231	0.0000	0.0652	0.0067	0.0000	0.0227	0.0000	0.0851	0.9544	0.9544
Uncultured bacterium 04	0.0006	0.0000	0.0017	0.0000	0.0049	0.1509	0.0000	0.5635	0.0000	20.1086	0.9544	0.9544
Anaeroplasmatales	0.0000	0.0000	0.0000	0.0000	0.0000	0.0000	0.0000	0.0000	0.0000	0.0000	NA	NA
Chloroplast transformation vector pL300PrrnG10L0CT1	0.0000	0.0000	0.0000	0.0000	0.0000	0.0000	0.0000	0.0000	0.0000	0.0000	NA	NA

Chorispora bungeana	0.0000	0.0000	0.0000	0.0000	0.0000	0.0000	0.0000	0.0000	0.0000	0.0000	NA	NA
Cocos nucifera (coconut palm)	0.0000	0.0000	0.0000	0.0000	0.0000	0.0000	0.0000	0.0000	0.0000	0.0000	NA	NA
Fraxinus excelsior (European ash)	0.0000	0.0000	0.0000	0.0000	0.0000	0.0000	0.0000	0.0000	0.0000	0.0000	NA	NA
Helianthus giganteus	0.0000	0.0000	0.0000	0.0000	0.0000	0.0000	0.0000	0.0000	0.0000	0.0000	NA	NA
Phoenix dactylifera (date palm)	0.0000	0.0000	0.0000	0.0000	0.0000	0.0000	0.0000	0.0000	0.0000	0.0000	NA	NA
Portulaca oleracea	0.0000	0.0000	0.0000	0.0000	0.0000	0.0000	0.0000	0.0000	0.0000	0.0000	NA	NA
Rickettsiales	0.0000	0.0000	0.0000	0.0000	0.0000	0.0000	0.0000	0.0000	0.0000	0.0000	NA	NA
Spirochaetales	0.0000	0.0000	0.0000	0.0000	0.0000	0.0000	0.0000	0.0000	0.0000	0.0000	NA	NA
Synergistales	0.0000	0.0000	0.0000	0.0000	0.0000	0.0000	0.0000	0.0000	0.0000	0.0000	NA	NA
Syngonanthus chrysanthus	0.0000	0.0000	0.0000	0.0000	0.0000	0.0000	0.0000	0.0000	0.0000	0.0000	NA	NA
Uncultured bacterium 01	0.0000	0.0000	0.0000	0.0000	0.0000	0.0000	0.0000	0.0000	0.0000	0.0000	NA	NA
Uncultured bacterium 03	0.0000	0.0000	0.0000	0.0000	0.0000	0.0000	0.0000	0.0000	0.0000	0.0000	NA	NA
Uncultured organism	0.0000	0.0000	0.0000	0.0000	0.0000	0.0000	0.0000	0.0000	0.0000	0.0000	NA	NA
VadinHA64	0.0000	0.0000	0.0000	0.0000	0.0000	0.0000	0.0000	0.0000	0.0000	0.0000	NA	NA

SD—standard deviation; NA—not available; *p*-value—calculated with the non-parametric Mann–Whitney test; *p*-adj.—p values adjusted with Benjamini–Hochberg FDR for multiple comparisons; red font—statistically significant results ($p < 0.05$).

Table S4. The percentage abundance of the intestinal microbiota in the study group of COVID-19 patients and healthy controls at the family level.

Family	COVID-19 patients, n = 8					Controls, n = 14					*p*- value	*p*- adj.
	Mean	Median	SD	Min.	Max.	Mean	Median	SD	Min.	Max.		
Verrucomicrobiaceae	0.7759	0.2940	10.3628	0.0496	40.1029	0.0238	0.0160	0.0286	0.0000	0.0923	**0.0004**	**0.0335**
Neisseriaceae	0.0179	0.0037	0.0312	0.0000	0.0854	0.0010	0.0000	0.0039	0.0000	0.0144	**0.0091**	0.3319
Leuconostocaceae	0.0058	0.0000	0.0104	0.0000	0.0285	0.0000	0.0000	0.0000	0.0000	0.0000	**0.0191**	0.3319
Phyllobacteriaceae	0.0077	0.0000	0.0133	0.0000	0.0366	0.0000	0.0000	0.0000	0.0000	0.0000	**0.0191**	0.3319
Uncultured bacterium-06	0.0130	0.0000	0.0198	0.0000	0.0494	0.0000	0.0000	0.0000	0.0000	0.0000	**0.0191**	0.3319
Lachnospiraceae	380.4910	370.9509	70.9057	240.1232	490.4323	290.3338	280.5685	110.9055	60.0064	570.6092	**0.0265**	0.3848
Clostridiaceae 1	0.6221	0.5478	0.4530	0.0784	10.4514	0.2904	0.1021	0.4307	0.0119	10.3609	**0.0316**	0.3922
Cytophagaceae	0.1704	0.1953	0.0772	0.0364	0.2621	0.0888	0.0314	0.1356	0.0000	0.3998	0.0515	0.5127
Nocardiaceae	0.0060	0.0000	0.0119	0.0000	0.0319	0.0000	0.0000	0.0000	0.0000	0.0000	0.0648	0.5127
PAUC34f	0.0009	0.0000	0.0018	0.0000	0.0049	0.0000	0.0000	0.0000	0.0000	0.0000	0.0648	0.5127
{Unknown Family} Bacteroidales	0.0016	0.0000	0.0037	0.0000	0.0106	0.0000	0.0000	0.0000	0.0000	0.0000	0.0648	0.5127
Uncultured bacterium-02	0.4219	0.2925	0.4757	0.0652	10.5312	0.1956	0.0473	0.2737	0.0000	0.8772	0.0809	0.5612
Peptostreptococcaceae	10.2765	0.1600	20.7242	0.0071	70.8880	10.4430	0.7267	20.3884	0.0038	80.9284	0.0945	0.5612
Kordiimonadaceae	0.0492	0.0000	0.0966	0.0000	0.2602	0.0096	0.0000	0.0358	0.0000	0.1338	0.1046	0.5612
Pasteurellaceae	0.0000	0.0000	0.0000	0.0000	0.0000	0.0028	0.0000	0.0052	0.0000	0.0158	0.1159	0.5612
Erysipelotrichaceae	10.1570	10.2447	0.3857	0.2911	10.4662	10.7849	0.5518	20.8593	0.0978	90.8891	0.1246	0.5612
Streptococcaceae	0.8009	0.1327	10.3640	0.0064	30.7607	0.0526	0.0367	0.0646	0.0000	0.2346	0.1246	0.5612
Prevotellaceae	0.7822	0.5013	0.9208	0.0000	20.8523	50.7599	20.0447	80.8387	0.0072	270.7800	0.1423	0.5612
Propionibacteriaceae	0.0173	0.0000	0.0315	0.0000	0.0841	0.0019	0.0000	0.0058	0.0000	0.0217	0.1778	0.5612
Veillonellaceae	0.2593	0.2688	0.1903	0.0041	0.5516	0.1755	0.0644	0.2683	0.0000	0.8421	0.1827	0.5612
Desulfovibrionaceae	0.0270	0.0211	0.0285	0.0000	0.0838	0.0194	0.0019	0.0384	0.0000	0.1412	0.1840	0.5612
Campylobacteraceae	0.0000	0.0000	0.0000	0.0000	0.0000	0.2294	0.0000	0.8275	0.0000	30.1036	0.1885	0.5612
Uncultured	0.0000	0.0000	0.0000	0.0000	0.0000	0.0068	0.0000	0.0175	0.0000	0.0642	0.1885	0.5612
Actinomycetaceae	0.0029	0.0000	0.0083	0.0000	0.0235	0.0000	0.0000	0.0000	0.0000	0.0000	0.2193	0.5612
Aerococcaceae	0.0006	0.0000	0.0017	0.0000	0.0049	0.0000	0.0000	0.0000	0.0000	0.0000	0.2193	0.5612
Flavobacteriaceae	0.0004	0.0000	0.0012	0.0000	0.0035	0.0000	0.0000	0.0000	0.0000	0.0000	0.2193	0.5612
ML635J-40 aquatic group	0.0009	0.0000	0.0025	0.0000	0.0071	0.0000	0.0000	0.0000	0.0000	0.0000	0.2193	0.5612
Rs-D42	0.0054	0.0000	0.0152	0.0000	0.0430	0.0000	0.0000	0.0000	0.0000	0.0000	0.2193	0.5612
Thermoanaerobacteraceae	0.0003	0.0000	0.0009	0.0000	0.0025	0.0000	0.0000	0.0000	0.0000	0.0000	0.2193	0.5612
Uncultured Bacillales bacterium	0.0062	0.0000	0.0174	0.0000	0.0494	0.0000	0.0000	0.0000	0.0000	0.0000	0.2193	0.5612
Uncultured Firmicutes bacterium	0.0018	0.0000	0.0050	0.0000	0.0141	0.0000	0.0000	0.0000	0.0000	0.0000	0.2193	0.5612
Unidentified	0.0004	0.0000	0.0012	0.0000	0.0035	0.0000	0.0000	0.0000	0.0000	0.0000	0.2193	0.5612
{Unknown Class} Bacteroidetes	0.0014	0.0000	0.0039	0.0000	0.0111	0.0000	0.0000	0.0000	0.0000	0.0000	0.2193	0.5612
{Unknown Phylum} Bacteria	0.0010	0.0000	0.0028	0.0000	0.0078	0.0000	0.0000	0.0000	0.0000	0.0000	0.2193	0.5612
Enterococcaceae	0.0316	0.0000	0.0628	0.0000	0.1687	0.0005	0.0000	0.0019	0.0000	0.0072	0.2299	0.5715
Bradyrhizobiaceae	0.0157	0.0000	0.0235	0.0000	0.0610	0.0014	0.0000	0.0029	0.0000	0.0077	0.2583	0.6242
Peptococcaceae	0.0013	0.0000	0.0026	0.0000	0.0071	0.0003	0.0000	0.0010	0.0000	0.0038	0.2774	0.6522
Bacillaceae	0.0000	0.0000	0.0000	0.0000	0.0000	0.0291	0.0000	0.0836	0.0000	0.3029	0.3050	0.6633
Moraxellaceae	0.0000	0.0000	0.0000	0.0000	0.0000	0.0011	0.0000	0.0028	0.0000	0.0080	0.3050	0.6633
Uncultured sediment bacterium	0.0000	0.0000	0.0000	0.0000	0.0000	0.0006	0.0000	0.0014	0.0000	0.0040	0.3050	0.6633
Rhodospirillaceae	0.4839	0.2362	0.5640	0.0000	10.4108	10.1767	0.0058	20.1791	0.0000	60.3184	0.3276	0.6951
Christensenellaceae	0.3832	0.1579	0.4826	0.0183	10.3839	0.7822	0.5379	10.0825	0.0000	40.2518	0.4324	0.7103
S24-7	0.2614	0.1705	0.2158	0.0366	0.6101	0.2418	0.0549	0.2731	0.0098	0.8638	0.4325	0.7103
Acidaminococcaceae	0.1189	0.0823	0.1313	0.0000	0.3084	0.0843	0.0140	0.1358	0.0000	0.3732	0.4665	0.7103
Defluviitaleaceae	0.0604	0.0437	0.0522	0.0107	0.1618	0.0817	0.0378	0.1412	0.0000	0.4489	0.4723	0.7103
Ruminococcaceae	160.9835	170.8042	70.4216	50.4500	30.1650	220.2637	220.4142	130.4185	20.1080	490.6346	0.4736	0.7103
VadinBB60	0.0283	0.0000	0.0800	0.0000	0.2263	0.0274	0.0000	0.0685	0.0000	0.2308	0.4857	0.7103

Anaerolineaceae	0.0000	0.0000	0.0000	0.0000	0.0000	0.0050	0.0000	0.0186	0.0000	0.0697	0.5083	0.7103
Carnobacteriaceae	0.0000	0.0000	0.0000	0.0000	0.0000	0.0003	0.0000	0.0010	0.0000	0.0038	0.5083	0.7103
Family XI-02	0.0000	0.0000	0.0000	0.0000	0.0000	0.0005	0.0000	0.0019	0.0000	0.0072	0.5083	0.7103
Hydrogenophilaceae	0.0000	0.0000	0.0000	0.0000	0.0000	0.0011	0.0000	0.0041	0.0000	0.0153	0.5083	0.7103
Methanobacteriaceae	0.0000	0.0000	0.0000	0.0000	0.0000	0.0018	0.0000	0.0067	0.0000	0.0251	0.5083	0.7103
Methylobacteriaceae	0.0000	0.0000	0.0000	0.0000	0.0000	0.0005	0.0000	0.0020	0.0000	0.0076	0.5083	0.7103
Nitrospiraceae	0.0000	0.0000	0.0000	0.0000	0.0000	0.0005	0.0000	0.0020	0.0000	0.0076	0.5083	0.7103
Paenibacillaceae	0.0000	0.0000	0.0000	0.0000	0.0000	0.0027	0.0000	0.0102	0.0000	0.0383	0.5083	0.7103
Pseudomonadaceae	0.0000	0.0000	0.0000	0.0000	0.0000	0.8134	0.0000	30.0436	0.0000	110.3882	0.5083	0.7103
Puniceicoccaceae	0.0000	0.0000	0.0000	0.0000	0.0000	0.0003	0.0000	0.0010	0.0000	0.0038	0.5083	0.7103
Sphingobacteriaceae	0.0000	0.0000	0.0000	0.0000	0.0000	0.0011	0.0000	0.0041	0.0000	0.0152	0.5083	0.7103
Sphingomonadaceae	0.0000	0.0000	0.0000	0.0000	0.0000	0.0005	0.0000	0.0020	0.0000	0.0076	0.5083	0.7103
Thermoplasmatales Incertae Sedis	0.0000	0.0000	0.0000	0.0000	0.0000	0.0003	0.0000	0.0011	0.0000	0.0040	0.5083	0.7103
Uncultured bacterium-07	0.0000	0.0000	0.0000	0.0000	0.0000	0.0003	0.0000	0.0011	0.0000	0.0043	0.5083	0.7103
Uncultured bacterium-08	0.0000	0.0000	0.0000	0.0000	0.0000	0.0061	0.0000	0.0227	0.0000	0.0851	0.5083	0.7103
Family XIII	0.0315	0.0267	0.0299	0.0041	0.0980	0.0400	0.0106	0.0607	0.0000	0.2051	0.5143	0.7103
Oxalobacteraceae	0.0015	0.0000	0.0030	0.0000	0.0081	0.1317	0.0000	0.4917	0.0000	10.8399	0.5770	0.7843
Alcaligenaceae	0.2372	0.1349	0.3502	0.0072	10.0749	0.8238	0.1918	10.4666	0.0000	50.2236	0.6087	0.8024
Porphyromonadaceae	40.0848	40.0886	20.0206	0.4653	60.7497	50.3344	40.7000	40.4557	0.0308	120.8743	0.6087	0.8024
Uncultured bacterium-05	0.0009	0.0000	0.0018	0.0000	0.0049	0.1509	0.0000	0.5635	0.0000	20.1086	0.6481	0.8240
Micrococcaceae	0.0138	0.0030	0.0329	0.0000	0.0952	0.0283	0.0000	0.0751	0.0000	0.2780	0.6547	0.8240
Coriobacteriaceae	0.5094	0.3626	0.4081	0.0736	10.1080	0.7893	0.3324	0.9334	0.0000	30.1898	0.6573	0.8240
Family III	0.0146	0.0000	0.0412	0.0000	0.1165	0.0063	0.0000	0.0234	0.0000	0.0876	0.6816	0.8240
Uncultured organism-02	0.0020	0.0000	0.0056	0.0000	0.0159	0.0006	0.0000	0.0022	0.0000	0.0080	0.6816	0.8240
Uncultured bacterium-09	0.0080	0.0000	0.0204	0.0000	0.0582	0.0807	0.0000	0.1680	0.0000	0.4700	0.6956	0.8240
Bacteroidaceae	210.0875	210.2925	140.0068	30.0512	40.1683	190.5739	160.7984	140.7254	0.2653	430.1085	0.7074	0.8240
Rikenellaceae	10.2424	10.0539	0.7147	0.1596	20.5915	20.5900	10.3893	30.4216	0.0152	120.0497	0.7074	0.8240
Lactobacillaceae	0.0186	0.0000	0.0352	0.0000	0.0956	0.0509	0.0000	0.1679	0.0000	0.6321	0.7103	0.8240
{Unknown Family} Clostridiales	0.0015	0.0000	0.0028	0.0000	0.0065	0.0030	0.0000	0.0092	0.0000	0.0342	0.7226	0.8272
Halanaerobiaceae	0.0719	0.0000	0.1950	0.0000	0.5542	0.0120	0.0000	0.0258	0.0000	0.0815	0.7727	0.8425
CFT112H7	0.0003	0.0000	0.0008	0.0000	0.0024	0.0009	0.0000	0.0034	0.0000	0.0125	0.7844	0.8425
Fusobacteriaceae	0.0004	0.0000	0.0011	0.0000	0.0032	0.0005	0.0000	0.0020	0.0000	0.0076	0.7844	0.8425
Uncultured organism-03	0.0013	0.0000	0.0037	0.0000	0.0106	0.0095	0.0000	0.0354	0.0000	0.1326	0.7844	0.8425
{Unknown Class} Firmicutes	0.0004	0.0000	0.0011	0.0000	0.0032	0.0003	0.0000	0.0011	0.0000	0.0043	0.7844	0.8425
Enterobacteriaceae	80.4430	0.0175	160.4994	0.0000	440.4962	30.8389	0.0546	130.4746	0.0000	50.6274	0.8082	0.8575
Victivallaceae	0.0004	0.0000	0.0012	0.0000	0.0035	0.0019	0.0000	0.0050	0.0000	0.0153	0.8638	0.8849
Xanthomonadaceae	0.0009	0.0000	0.0025	0.0000	0.0071	0.0165	0.0000	0.0559	0.0000	0.2097	0.8638	0.8849
Bifidobacteriaceae	0.9722	0.3390	10.1781	0.0212	30.1712	10.3891	0.6926	10.5926	0.0038	40.8331	0.8645	0.8849
Uncultured bacterium-04	0.0156	0.0000	0.0442	0.0000	0.1251	0.0142	0.0000	0.0458	0.0000	0.1711	0.9544	0.9653
Planococcaceae	0.0286	0.0000	0.0567	0.0000	0.1525	0.1683	0.0000	0.5953	0.0000	20.2351	0.9653	0.9653
Anaeroplasmataceae	0.0000	0.0000	0.0000	0.0000	0.0000	0.0000	0.0000	0.0000	0.0000	0.0000	NA	NA
BS11 gut group	0.0000	0.0000	0.0000	0.0000	0.0000	0.0000	0.0000	0.0000	0.0000	0.0000	NA	NA
Burkholderiaceae	0.0000	0.0000	0.0000	0.0000	0.0000	0.0000	0.0000	0.0000	0.0000	0.0000	NA	NA
Chloroplast transformation vector pL3-PrrnG10L-CT1	0.0000	0.0000	0.0000	0.0000	0.0000	0.0000	0.0000	0.0000	0.0000	0.0000	NA	NA
Chorispora bungeana	0.0000	0.0000	0.0000	0.0000	0.0000	0.0000	0.0000	0.0000	0.0000	0.0000	NA	NA
Cocos nucifera (coconut palm)	0.0000	0.0000	0.0000	0.0000	0.0000	0.0000	0.0000	0.0000	0.0000	0.0000	NA	NA
Cryomorphaceae	0.0000	0.0000	0.0000	0.0000	0.0000	0.0000	0.0000	0.0000	0.0000	0.0000	NA	NA
Eubacteriaceae	0.0000	0.0000	0.0000	0.0000	0.0000	0.0000	0.0000	0.0000	0.0000	0.0000	NA	NA
Family XI-01	0.0000	0.0000	0.0000	0.0000	0.0000	0.0000	0.0000	0.0000	0.0000	0.0000	NA	NA
Fraxinus excelsior (European ash)	0.0000	0.0000	0.0000	0.0000	0.0000	0.0000	0.0000	0.0000	0.0000	0.0000	NA	NA
Helianthus giganteus	0.0000	0.0000	0.0000	0.0000	0.0000	0.0000	0.0000	0.0000	0.0000	0.0000	NA	NA
Mitochondria	0.0000	0.0000	0.0000	0.0000	0.0000	0.0000	0.0000	0.0000	0.0000	0.0000	NA	NA

NS9 marine group	0.0000	0.0000	0.0000	0.0000	0.0000	0.0000	0.0000	0.0000	0.0000	0.0000	NA	NA
PeH15	0.0000	0.0000	0.0000	0.0000	0.0000	0.0000	0.0000	0.0000	0.0000	0.0000	NA	NA
Phoenix dactylifera (date palm)	0.0000	0.0000	0.0000	0.0000	0.0000	0.0000	0.0000	0.0000	0.0000	0.0000	NA	NA
Portulaca oleracea	0.0000	0.0000	0.0000	0.0000	0.0000	0.0000	0.0000	0.0000	0.0000	0.0000	NA	NA
Rhodobiaceae	0.0000	0.0000	0.0000	0.0000	0.0000	0.0000	0.0000	0.0000	0.0000	0.0000	NA	NA
Spirochaetaceae	0.0000	0.0000	0.0000	0.0000	0.0000	0.0000	0.0000	0.0000	0.0000	0.0000	NA	NA
Synergistaceae	0.0000	0.0000	0.0000	0.0000	0.0000	0.0000	0.0000	0.0000	0.0000	0.0000	NA	NA
Syngonanthus chrysanthus	0.0000	0.0000	0.0000	0.0000	0.0000	0.0000	0.0000	0.0000	0.0000	0.0000	NA	NA
Uncultured bacterium-01	0.0000	0.0000	0.0000	0.0000	0.0000	0.0000	0.0000	0.0000	0.0000	0.0000	NA	NA
Uncultured bacterium-03	0.0000	0.0000	0.0000	0.0000	0.0000	0.0000	0.0000	0.0000	0.0000	0.0000	NA	NA
Uncultured bacterium-10	0.0000	0.0000	0.0000	0.0000	0.0000	0.0000	0.0000	0.0000	0.0000	0.0000	NA	NA
Uncultured organism-01	0.0000	0.0000	0.0000	0.0000	0.0000	0.0000	0.0000	0.0000	0.0000	0.0000	NA	NA
Uncultured rumen bacterium	0.0000	0.0000	0.0000	0.0000	0.0000	0.0000	0.0000	0.0000	0.0000	0.0000	NA	NA

SD—standard deviation; NA—not available; *p*-value—calculated with the non-parametric Mann–Whitney test; *p*-adj.—*p* values adjusted with Benjamini–Hochberg FDR for multiple comparisons; red font—statistically significant results ($p < 0.05$).

Table S5. The percentage abundance of the intestinal microbiota in the study group of COVID-19 patients and healthy controls at the genus level.

Genus	COVID-19 patients, *n* = 8					Controls, *n* = 14					*p*- value	*p*- adj.
	**Mean**	**Median**	**SD**	**Min.**	**Max.**	**Mean**	**Median**	**SD**	**Min.**	**Max.**		
Akkermansia	0.7316	0.1971	10.3831	0.0251	40.1029	0.0238	0.0160	0.0286	0.0000	0.0923	**0.0017**	0.3093
Neisseria	0.0136	0.0037	0.0292	0.0000	0.0854	0.0010	0.0000	0.0039	0.0000	0.0144	**0.0117**	0.6507
Bilophila	0.0215	0.0125	0.0275	0.0000	0.0838	0.0044	0.0000	0.0089	0.0000	0.0251	**0.0178**	0.6507
Adlercreutzia	0.0029	0.0000	0.0050	0.0000	0.0143	0.0000	0.0000	0.0000	0.0000	0.0000	**0.0191**	0.6507
Blautia	80.5187	70.9408	50.2602	10.7165	160.9819	40.2086	30.4824	20.6461	0.2433	80.8204	0.0518	0.6507
Enterorhabdus	0.0187	0.0060	0.0389	0.0000	0.1139	0.0017	0.0000	0.0038	0.0000	0.0125	0.0574	0.6507
Epulopiscium	0.2171	0.2214	0.1755	0.0179	0.5076	0.0951	0.0484	0.1149	0.0000	0.3754	0.0602	0.6507
Anaerostipes	10.9278	20.0260	10.3330	0.1718	30.3236	0.9981	0.6294	0.9267	0.0543	20.9183	0.0605	0.6507
Gordonibacter	0.0008	0.0000	0.0016	0.0000	0.0041	0.0000	0.0000	0.0000	0.0000	0.0000	0.0648	0.6507
Leuconostoc	0.0035	0.0000	0.0085	0.0000	0.0244	0.0000	0.0000	0.0000	0.0000	0.0000	0.0648	0.6507
Marvinbryantia	0.0009	0.0000	0.0017	0.0000	0.0041	0.0000	0.0000	0.0000	0.0000	0.0000	0.0648	0.6507
Phyllobacterium	0.0054	0.0000	0.0128	0.0000	0.0366	0.0000	0.0000	0.0000	0.0000	0.0000	0.0648	0.6507
Uncultured Bacillales bacterium	0.0097	0.0000	0.0189	0.0000	0.0494	0.0000	0.0000	0.0000	0.0000	0.0000	0.0648	0.6507
Uncultured bacterium-17	0.0107	0.0000	0.0202	0.0000	0.0494	0.0000	0.0000	0.0000	0.0000	0.0000	0.0648	0.6507
{Unknown Family} Bacteroidales	0.0016	0.0000	0.0037	0.0000	0.0106	0.0000	0.0000	0.0000	0.0000	0.0000	0.0648	0.6507
{Unknown Phylum} Bacteria	0.0014	0.0000	0.0029	0.0000	0.0078	0.0000	0.0000	0.0000	0.0000	0.0000	0.0648	0.6507
Incertae Sedis-05	0.3064	0.0301	0.5335	0.0035	10.2988	0.6742	0.3148	10.0278	0.0038	30.7574	0.0705	0.6507
Kordiimonas	0.0952	0.0000	0.1863	0.0000	0.4976	0.0096	0.0000	0.0358	0.0000	0.1338	0.0847	0.6507
Turicibacter	0.1523	0.1137	0.1641	0.0000	0.4580	0.2521	0.0081	0.8683	0.0000	30.2669	0.0893	0.6507
Uncultured-01	0.0645	0.0326	0.0845	0.0000	0.2466	0.0150	0.0000	0.0232	0.0000	0.0748	0.0921	0.6507
Hymenobacter	0.1470	0.1841	0.0932	0.0143	0.2621	0.0888	0.0314	0.1356	0.0000	0.3998	0.1083	0.6507
Megasphaera	0.0000	0.0000	0.0000	0.0000	0.0000	0.0195	0.0000	0.0490	0.0000	0.1676	0.1159	0.6507
Incertae Sedis-06	0.5949	0.4226	0.7282	0.0785	20.3636	0.3076	0.2367	0.2626	0.0197	0.9136	0.1246	0.6507
Streptococcus	0.5465	0.1254	10.1714	0.0064	30.4273	0.0512	0.0312	0.0653	0.0000	0.2346	0.1246	0.6507
Megamonas	0.0051	0.0000	0.0092	0.0000	0.0251	0.0136	0.0000	0.0511	0.0000	0.1910	0.1282	0.6507
Quinella	0.0281	0.0000	0.0747	0.0000	0.2127	0.0160	0.0000	0.0599	0.0000	0.2243	0.1282	0.6507
Phascolarctobacterium	0.0973	0.0140	0.1325	0.0000	0.3084	0.0482	0.0000	0.1086	0.0000	0.3486	0.1286	0.6507
Uncultured-03	0.0232	0.0000	0.0632	0.0000	0.1795	30.4522	0.0154	70.7409	0.0000	220.2001	0.1354	0.6507
Propionibacterium	0.0173	0.0000	0.0315	0.0000	0.0841	0.0019	0.0000	0.0058	0.0000	0.0217	0.1778	0.6507
Flavonifractor	0.0096	0.0028	0.0161	0.0000	0.0468	0.0042	0.0000	0.0097	0.0000	0.0344	0.1802	0.6507
Uncultured bacterium-05	0.3792	0.2049	0.4976	0.0107	10.5312	0.1956	0.0473	0.2737	0.0000	0.8772	0.1820	0.6507
Clostridium sensu stricto 1	0.4751	0.3941	0.4250	0.0143	10.3289	0.2884	0.1021	0.4319	0.0119	10.3609	0.1832	0.6507
Acidaminococcus	0.0000	0.0000	0.0000	0.0000	0.0000	0.0361	0.0000	0.1015	0.0000	0.3732	0.1885	0.6507
Butyrivibrio	0.0000	0.0000	0.0000	0.0000	0.0000	0.0938	0.0000	0.3057	0.0000	10.1468	0.1885	0.6507
Campylobacter	0.0000	0.0000	0.0000	0.0000	0.0000	0.2294	0.0000	0.8275	0.0000	30.1036	0.1885	0.6507
Intestinimonas	0.0000	0.0000	0.0000	0.0000	0.0000	0.0025	0.0000	0.0053	0.0000	0.0166	0.1885	0.6507
Paraprevotella	0.4089	0.0216	0.9655	0.0000	20.7796	0.1011	0.0000	0.3611	0.0000	10.3552	0.1914	0.6507
Roseburia	30.1997	30.3612	10.3374	10.5369	40.8095	20.3465	20.0347	10.8365	0.0000	60.7251	0.2067	0.6507
Uncultured-02	10.0196	10.0823	0.5698	0.0797	10.6723	0.7509	0.4706	0.8545	0.0000	20.6009	0.2067	0.6507
Uncultured-14	0.6336	0.6336	0.4609	0.0924	10.4292	0.3819	0.4188	0.2743	0.0000	0.7725	0.2067	0.6507
Actinomyces	0.0015	0.0000	0.0042	0.0000	0.0118	0.0000	0.0000	0.0000	0.0000	0.0000	0.2193	0.6507
Arcanobacterium	0.0010	0.0000	0.0028	0.0000	0.0078	0.0000	0.0000	0.0000	0.0000	0.0000	0.2193	0.6507
Cetobacterium	0.0004	0.0000	0.0011	0.0000	0.0032	0.0000	0.0000	0.0000	0.0000	0.0000	0.2193	0.6507
Fructobacillus	0.0005	0.0000	0.0014	0.0000	0.0041	0.0000	0.0000	0.0000	0.0000	0.0000	0.2193	0.6507
Gelria	0.0003	0.0000	0.0009	0.0000	0.0025	0.0000	0.0000	0.0000	0.0000	0.0000	0.2193	0.6507
Ignavigranum	0.0006	0.0000	0.0017	0.0000	0.0049	0.0000	0.0000	0.0000	0.0000	0.0000	0.2193	0.6507
Paludibacter	0.0003	0.0000	0.0008	0.0000	0.0024	0.0000	0.0000	0.0000	0.0000	0.0000	0.2193	0.6507
Rhodococcus	0.0040	0.0000	0.0113	0.0000	0.0319	0.0000	0.0000	0.0000	0.0000	0.0000	0.2193	0.6507
Selenomonas	0.0004	0.0000	0.0011	0.0000	0.0032	0.0000	0.0000	0.0000	0.0000	0.0000	0.2193	0.6507
Trueperella	0.0005	0.0000	0.0014	0.0000	0.0039	0.0000	0.0000	0.0000	0.0000	0.0000	0.2193	0.6507
Uncultured Bacteroidetes bacterium-01	0.0009	0.0000	0.0025	0.0000	0.0071	0.0000	0.0000	0.0000	0.0000	0.0000	0.2193	0.6507
Uncultured Firmicutes bacterium	0.0018	0.0000	0.0050	0.0000	0.0141	0.0000	0.0000	0.0000	0.0000	0.0000	0.2193	0.6507

Uncultured bacterium-08	0.0006	0.0000	0.0017	0.0000	0.0049	0.0000	0.0000	0.0000	0.0000	0.0000	0.2193	0.6507
Uncultured bacterium-09	0.0054	0.0000	0.0152	0.0000	0.0430	0.0000	0.0000	0.0000	0.0000	0.0000	0.2193	0.6507
Uncultured bacterium-21	0.0031	0.0000	0.0089	0.0000	0.0251	0.0000	0.0000	0.0000	0.0000	0.0000	0.2193	0.6507
Uncultured rumen bacterium-01	0.0190	0.0000	0.0538	0.0000	0.1521	0.0000	0.0000	0.0000	0.0000	0.0000	0.2193	0.6507
Uncultured-04	0.0004	0.0000	0.0012	0.0000	0.0035	0.0000	0.0000	0.0000	0.0000	0.0000	0.2193	0.6507
Uncultured-11	0.0009	0.0000	0.0025	0.0000	0.0071	0.0000	0.0000	0.0000	0.0000	0.0000	0.2193	0.6507
Unidentified	0.0004	0.0000	0.0012	0.0000	0.0035	0.0000	0.0000	0.0000	0.0000	0.0000	0.2193	0.6507
{Unknown Class} Bacteroidetes	0.0014	0.0000	0.0039	0.0000	0.0111	0.0000	0.0000	0.0000	0.0000	0.0000	0.2193	0.6507
{Unknown Genus} Puniceicoccaceae	0.0031	0.0000	0.0089	0.0000	0.0251	0.0000	0.0000	0.0000	0.0000	0.0000	0.2193	0.6507
Eggerthella	0.0032	0.0000	0.0066	0.0000	0.0183	0.0003	0.0000	0.0010	0.0000	0.0038	0.2299	0.6570
Uncultured organism-05	0.0058	0.0000	0.0114	0.0000	0.0304	0.0006	0.0000	0.0022	0.0000	0.0080	0.2299	0.6570
Faecalibacterium	50.8263	30.2673	50.0413	0.4675	130.2204	80.0863	60.6540	50.8374	0.0103	190.9471	0.2323	0.6570
Parasutterella	0.0450	0.0097	0.0817	0.0000	0.2416	0.4396	0.0000	10.3889	0.0000	50.2236	0.2480	0.6900
Collinsella	0.3948	0.2274	0.3823	0.0537	10.0049	0.7230	0.3194	0.8757	0.0000	30.1648	0.2601	0.6900
Allisonella	0.0186	0.0000	0.0352	0.0000	0.0943	0.0089	0.0000	0.0304	0.0000	0.1142	0.2851	0.6900
Sarcina	0.0166	0.0000	0.0429	0.0000	0.1226	0.0020	0.0000	0.0051	0.0000	0.0150	0.2851	0.6900
Lachnospira	0.1477	0.0779	0.1980	0.0073	0.6050	0.2000	0.0115	0.3851	0.0000	10.1634	0.2887	0.6900
Prevotella	0.3772	0.2911	0.3969	0.0000	10.2214	10.5949	10.2171	20.0188	0.0000	70.3474	0.2896	0.6900
Uncultured-12	0.9595	0.1061	20.2829	0.0035	60.5892	0.7679	0.2340	10.3821	0.0000	50.1710	0.2900	0.6900
Thalassospira	0.7103	0.2362	0.9180	0.0000	20.5166	10.1767	0.0058	20.1791	0.0000	60.3184	0.2942	0.6900
Acetitomaculum	0.0000	0.0000	0.0000	0.0000	0.0000	0.0017	0.0000	0.0053	0.0000	0.0198	0.3050	0.6900
Acinetobacter	0.0000	0.0000	0.0000	0.0000	0.0000	0.0011	0.0000	0.0028	0.0000	0.0080	0.3050	0.6900
Bacillus	0.0000	0.0000	0.0000	0.0000	0.0000	0.0134	0.0000	0.0343	0.0000	0.1047	0.3050	0.6900
Citrobacter	0.0000	0.0000	0.0000	0.0000	0.0000	10.4287	0.0000	50.3446	0.0000	190.9980	0.3050	0.6900
Sporosarcina	0.0000	0.0000	0.0000	0.0000	0.0000	0.1605	0.0000	0.5971	0.0000	20.2351	0.3050	0.6900
Uncultured bacterium-13	0.0000	0.0000	0.0000	0.0000	0.0000	0.0007	0.0000	0.0017	0.0000	0.0052	0.3050	0.6900
Uncultured organism-02	0.0000	0.0000	0.0000	0.0000	0.0000	0.0022	0.0000	0.0058	0.0000	0.0192	0.3050	0.6900
Uncultured sediment bacterium	0.0000	0.0000	0.0000	0.0000	0.0000	0.0006	0.0000	0.0014	0.0000	0.0040	0.3050	0.6900
Dialister	0.1817	0.0947	0.2124	0.0000	0.5516	0.1116	0.0141	0.2241	0.0000	0.8310	0.3106	0.6940
Incertae Sedis-07	0.3284	0.0770	0.4724	0.0000	10.2774	0.1643	0.0078	0.3693	0.0000	10.3621	0.3146	0.6943
Dorea	0.5130	0.4244	0.4152	0.0251	10.3553	0.4172	0.1297	0.5733	0.0241	10.8990	0.3223	0.6968
Alloprevotella	0.0007	0.0000	0.0020	0.0000	0.0056	0.6116	0.0000	10.6893	0.0000	60.3646	0.3291	0.6968
Pantoea	0.0079	0.0000	0.0154	0.0000	0.0407	0.9089	0.0000	30.4008	0.0000	120.7247	0.3311	0.6968
{Unknown Class} Firmicutes	0.0009	0.0000	0.0016	0.0000	0.0036	0.0003	0.0000	0.0011	0.0000	0.0043	0.3311	0.6968
Desulfovibrio	0.0056	0.0000	0.0158	0.0000	0.0448	0.0150	0.0000	0.0377	0.0000	0.1412	0.3388	0.7048
Butyricimonas	0.0128	0.0107	0.0095	0.0000	0.0251	0.0644	0.0239	0.1042	0.0000	0.3957	0.3541	0.7097
Incertae Sedis-04	110.3662	10.5632	50.3767	30.6263	170.9951	10.0430	80.0470	60.9700	30.7396	30.4944	0.3936	0.7097
Moryella	0.0062	0.0000	0.0126	0.0000	0.0366	0.0468	0.0049	0.1465	0.0000	0.5547	0.4123	0.7097
Alistipes	10.0468	10.0355	0.7772	0.1596	20.5915	20.5779	10.3893	30.4270	0.0152	120.0497	0.4325	0.7097
Uncultured bacterium-02	0.2501	0.1299	0.2220	0.0366	0.6101	0.2418	0.0549	0.2731	0.0098	0.8638	0.4325	0.7097
Anaerotruncus	0.1773	0.1132	0.2061	0.0000	0.5711	0.2332	0.0038	0.4597	0.0000	10.4581	0.4579	0.7097
Bifidobacterium	0.7704	0.1967	10.1728	0.0000	30.0244	10.2798	0.6692	10.4487	0.0038	40.1488	0.4736	0.7097
Uncultured-09	0.0267	0.0184	0.0320	0.0000	0.0980	0.0312	0.0094	0.0553	0.0000	0.2051	0.4906	0.7097
Halocella	0.0724	0.0018	0.1948	0.0000	0.5542	0.0120	0.0000	0.0258	0.0000	0.0815	0.5009	0.7097
Anaerosporobacter	0.0000	0.0000	0.0000	0.0000	0.0000	0.0003	0.0000	0.0010	0.0000	0.0038	0.5083	0.7097
Candidatus Methanomethylophilus	0.0000	0.0000	0.0000	0.0000	0.0000	0.0003	0.0000	0.0011	0.0000	0.0040	0.5083	0.7097
Carnobacterium	0.0000	0.0000	0.0000	0.0000	0.0000	0.0003	0.0000	0.0010	0.0000	0.0038	0.5083	0.7097
Cerasibacillus	0.0000	0.0000	0.0000	0.0000	0.0000	0.0005	0.0000	0.0020	0.0000	0.0077	0.5083	0.7097
Dialister sp. oral taxon 502	0.0000	0.0000	0.0000	0.0000	0.0000	0.0008	0.0000	0.0030	0.0000	0.0112	0.5083	0.7097
Ferritrophicum	0.0000	0.0000	0.0000	0.0000	0.0000	0.0011	0.0000	0.0041	0.0000	0.0153	0.5083	0.7097
Fusobacterium	0.0000	0.0000	0.0000	0.0000	0.0000	0.0005	0.0000	0.0020	0.0000	0.0076	0.5083	0.7097
Holdemania	0.0000	0.0000	0.0000	0.0000	0.0000	0.0004	0.0000	0.0014	0.0000	0.0052	0.5083	0.7097
Howardella	0.0000	0.0000	0.0000	0.0000	0.0000	0.0003	0.0000	0.0010	0.0000	0.0038	0.5083	0.7097
Incertae Sedis-01	0.0000	0.0000	0.0000	0.0000	0.0000	0.0003	0.0000	0.0011	0.0000	0.0040	0.5083	0.7097
Marine group	0.0000	0.0000	0.0000	0.0000	0.0000	0.0003	0.0000	0.0010	0.0000	0.0038	0.5083	0.7097
Methanobrevibacter	0.0000	0.0000	0.0000	0.0000	0.0000	0.0009	0.0000	0.0034	0.0000	0.0125	0.5083	0.7097

Methanosphaera	0.0000	0.0000	0.0000	0.0000	0.0000	0.0009	0.0000	0.0034	0.0000	0.0125	0.5083	0.7097
Methylobacterium	0.0000	0.0000	0.0000	0.0000	0.0000	0.0005	0.0000	0.0020	0.0000	0.0076	0.5083	0.7097
Mitsuokella	0.0000	0.0000	0.0000	0.0000	0.0000	0.0003	0.0000	0.0011	0.0000	0.0040	0.5083	0.7097
Mucilaginibacter	0.0000	0.0000	0.0000	0.0000	0.0000	0.0005	0.0000	0.0020	0.0000	0.0076	0.5083	0.7097
Nitrospira	0.0000	0.0000	0.0000	0.0000	0.0000	0.0005	0.0000	0.0020	0.0000	0.0076	0.5083	0.7097
Oceanobacillus	0.0000	0.0000	0.0000	0.0000	0.0000	0.0016	0.0000	0.0061	0.0000	0.0230	0.5083	0.7097
Oxalobacter	0.0000	0.0000	0.0000	0.0000	0.0000	0.0003	0.0000	0.0011	0.0000	0.0040	0.5083	0.7097
Paenibacillus	0.0000	0.0000	0.0000	0.0000	0.0000	0.0027	0.0000	0.0102	0.0000	0.0383	0.5083	0.7097
Parvimonas	0.0000	0.0000	0.0000	0.0000	0.0000	0.0005	0.0000	0.0019	0.0000	0.0072	0.5083	0.7097
Pediococcus	0.0000	0.0000	0.0000	0.0000	0.0000	0.0011	0.0000	0.0041	0.0000	0.0155	0.5083	0.7097
Pedobacter	0.0000	0.0000	0.0000	0.0000	0.0000	0.0005	0.0000	0.0020	0.0000	0.0076	0.5083	0.7097
Pseudomonas	0.0000	0.0000	0.0000	0.0000	0.0000	0.8134	0.0000	30.0436	0.0000	110.3882	0.5083	0.7097
Solobacterium	0.0000	0.0000	0.0000	0.0000	0.0000	0.0233	0.0000	0.0871	0.0000	0.3259	0.5083	0.7097
Sphingomonas	0.0000	0.0000	0.0000	0.0000	0.0000	0.0005	0.0000	0.0020	0.0000	0.0076	0.5083	0.7097
Uncultured bacterium-03	0.0000	0.0000	0.0000	0.0000	0.0000	0.0046	0.0000	0.0171	0.0000	0.0642	0.5083	0.7097
Uncultured bacterium-18	0.0000	0.0000	0.0000	0.0000	0.0000	0.0003	0.0000	0.0011	0.0000	0.0043	0.5083	0.7097
Uncultured bacterium-19	0.0000	0.0000	0.0000	0.0000	0.0000	0.0061	0.0000	0.0227	0.0000	0.0851	0.5083	0.7097
Uncultured-05	0.0000	0.0000	0.0000	0.0000	0.0000	0.0050	0.0000	0.0186	0.0000	0.0697	0.5083	0.7097
Uncultured-06	0.0000	0.0000	0.0000	0.0000	0.0000	0.0136	0.0000	0.0507	0.0000	0.1898	0.5083	0.7097
Uncultured-15	0.0000	0.0000	0.0000	0.0000	0.0000	0.0028	0.0000	0.0106	0.0000	0.0396	0.5083	0.7097
{Unknown Genus} Peptostreptococcaceae	0.0000	0.0000	0.0000	0.0000	0.0000	0.0009	0.0000	0.0034	0.0000	0.0125	0.5083	0.7097
Uncultured bacterium-07	0.0443	0.0000	0.0866	0.0000	0.2291	0.0142	0.0000	0.0458	0.0000	0.1711	0.5097	0.7097
Coprobacter	0.0195	0.0028	0.0335	0.0000	0.0874	0.0295	0.0143	0.0436	0.0000	0.1464	0.5424	0.7317
Edwardsiella	0.0319	0.0000	0.0876	0.0000	0.2486	0.0097	0.0000	0.0309	0.0000	0.1161	0.5427	0.7317
RC9 gut group	0.0020	0.0000	0.0058	0.0000	0.0163	0.0121	0.0000	0.0252	0.0000	0.0704	0.5428	0.7317
Slackia	0.0008	0.0000	0.0023	0.0000	0.0064	0.0493	0.0000	0.1771	0.0000	0.6645	0.5428	0.7317
Haemophilus	0.0087	0.0000	0.0247	0.0000	0.0698	0.0028	0.0000	0.0052	0.0000	0.0158	0.5458	0.7317
Uncultured-08	0.0346	0.0249	0.0253	0.0056	0.0758	0.0249	0.0269	0.0247	0.0000	0.0774	0.5607	0.7462
Incertae Sedis-03	0.0015	0.0000	0.0029	0.0000	0.0075	0.0029	0.0000	0.0096	0.0000	0.0361	0.5770	0.7567
Victivallis	0.0121	0.0000	0.0328	0.0000	0.0931	0.0019	0.0000	0.0050	0.0000	0.0153	0.5770	0.7567
Catenibacterium	0.0140	0.0000	0.0373	0.0000	0.1060	0.9629	0.0000	20.6796	0.0000	90.5507	0.5916	0.7704
{Unknown Genus} Lachnospiraceae	0.0164	0.0000	0.0427	0.0000	0.1220	0.0531	0.0000	0.1732	0.0000	0.6498	0.6018	0.7759
Parabacteroides	0.3905	0.1903	0.4134	0.0337	10.1862	0.3202	0.2364	0.3182	0.0000	0.9683	0.6086	0.7759
Subdoligranulum	10.9020	10.3792	10.7781	0.5367	60.0910	10.9282	10.8287	10.2334	0.0645	30.9354	0.6087	0.7759
Barnesiella	20.0559	10.7843	10.7403	0.0112	40.3347	30.5931	20.4177	30.7917	0.0000	10.3173	0.6572	0.8262
Pseudobutyrivibrio	20.0877	10.5076	20.1817	0.3348	70.0758	20.8313	10.1982	40.4030	0.0457	150.7375	0.6573	0.8262
Uncultured-16	0.0119	0.0000	0.0232	0.0000	0.0610	0.0014	0.0000	0.0029	0.0000	0.0077	0.6758	0.8279
Enterococcus	0.0211	0.0000	0.0597	0.0000	0.1687	0.0005	0.0000	0.0019	0.0000	0.0072	0.6816	0.8279
Morganella	0.0005	0.0000	0.0014	0.0000	0.0041	0.0003	0.0000	0.0010	0.0000	0.0038	0.6816	0.8279
Thermoanaerobacterium	0.0146	0.0000	0.0412	0.0000	0.1165	0.0063	0.0000	0.0234	0.0000	0.0876	0.6816	0.8279
Uncultured Thermoanaerobacterales bacterium	0.0275	0.0000	0.0777	0.0000	0.2199	0.0005	0.0000	0.0019	0.0000	0.0072	0.6816	0.8279
Ruminococcus	10.3568	50.5903	140.4687	0.7684	440.4808	60.0222	50.6359	50.7028	0.1213	180.7126	0.7074	0.8514
Enterobacter	0.3922	0.0000	10.0958	0.0000	30.1041	0.2165	0.0000	0.5272	0.0000	10.7386	0.7103	0.8514
{Unknown Family} Clostridiales	0.0015	0.0000	0.0028	0.0000	0.0065	0.0030	0.0000	0.0092	0.0000	0.0342	0.7226	0.8605
Planomicrobium	0.0191	0.0000	0.0539	0.0000	0.1525	0.0075	0.0000	0.0188	0.0000	0.0684	0.7609	0.8874
Christensenella	0.0004	0.0000	0.0011	0.0000	0.0032	0.0006	0.0000	0.0022	0.0000	0.0080	0.7844	0.8874
Klebsiella	0.0006	0.0000	0.0017	0.0000	0.0049	0.0010	0.0000	0.0039	0.0000	0.0144	0.7844	0.8874
Mogibacterium	0.0012	0.0000	0.0035	0.0000	0.0098	0.0059	0.0000	0.0220	0.0000	0.0824	0.7844	0.8874
Peptococcus	0.0004	0.0000	0.0011	0.0000	0.0032	0.0003	0.0000	0.0010	0.0000	0.0038	0.7844	0.8874
Uncultured bacterium-16	0.0003	0.0000	0.0008	0.0000	0.0024	0.0009	0.0000	0.0034	0.0000	0.0125	0.7844	0.8874
Uncultured organism-06	0.0013	0.0000	0.0037	0.0000	0.0106	0.0095	0.0000	0.0354	0.0000	0.1326	0.7844	0.8874
Uncultured-20	0.0005	0.0000	0.0014	0.0000	0.0041	0.4491	0.0000	10.6805	0.0000	60.2880	0.7844	0.8874
Bacteroides	20.7352	210.2925	140.3156	30.0512	40.1683	190.5739	160.7984	140.725 4	0.2653	430.1085	0.8112	0.9063
Uncultured-13	40.1415	40.0977	20.0309	10.5227	80.2819	50.6699	30.4822	60.2562	0.3825	190.2359	0.8112	0.9063
Dokdonella	0.0009	0.0000	0.0025	0.0000	0.0071	0.0165	0.0000	0.0559	0.0000	0.2097	0.8638	0.9483
Veillonella	0.0004	0.0000	0.0013	0.0000	0.0036	0.0020	0.0000	0.0062	0.0000	0.0231	0.8638	0.9483
Uncultured-10	70.5201	60.0657	30.7579	30.4010	130.7908	70.4795	70.6785	30.1779	10.7354	110.7428	0.8645	0.9483

Escherichia-Shigella	50.1935	0.0000	140.4975	0.0000	410.0709	0.8008	0.0019	20.7258	0.0000	10.2614	0.8815	0.9612
Lactobacillus	0.0127	0.0000	0.0336	0.0000	0.0956	0.0498	0.0000	0.1638	0.0000	0.6166	0.8962	0.9656
Lactococcus	0.0435	0.0000	0.1172	0.0000	0.3334	0.0015	0.0000	0.0025	0.0000	0.0076	0.8962	0.9656
Arthrobacter	0.0135	0.0020	0.0331	0.0000	0.0952	0.0283	0.0000	0.0751	0.0000	0.2780	0.9086	0.9722
Uncultured-07	0.6471	0.3633	0.7618	0.0183	20.1479	0.7817	0.5339	10.0826	0.0000	40.2518	0.9184	0.9722
Coprococcus	0.4353	0.4432	0.2693	0.0756	0.8025	0.5187	0.4313	0.5918	0.0000	20.0952	0.9185	0.9722
Massilia	0.0010	0.0000	0.0029	0.0000	0.0081	0.1314	0.0000	0.4906	0.0000	10.8359	0.9544	0.9728
Salmonella	0.0341	0.0000	0.0963	0.0000	0.2724	0.0238	0.0000	0.0694	0.0000	0.2534	0.9544	0.9728
Uncultured bacterium-15	0.0006	0.0000	0.0017	0.0000	0.0049	0.1509	0.0000	0.5635	0.0000	20.1086	0.9544	0.9728
{Unknown Genus} Ruminococcaceae	0.0125	0.0000	0.0353	0.0000	0.0997	0.0088	0.0000	0.0318	0.0000	0.1191	0.9544	0.9728
Uncultured bacterium-14	0.0209	0.0000	0.0567	0.0000	0.1611	0.0269	0.0000	0.0687	0.0000	0.2308	0.9629	0.9728
Uncultured bacterium-20	0.0133	0.0000	0.0234	0.0000	0.0582	0.0807	0.0000	0.1680	0.0000	0.4700	0.9671	0.9728
Gardnerella	0.0359	0.0177	0.0508	0.0000	0.1468	0.1093	0.0157	0.2293	0.0000	0.8420	0.9718	0.9728
Incertae Sedis-02	0.0181	0.0074	0.0331	0.0000	0.0981	0.0568	0.0081	0.1323	0.0000	0.4322	0.9718	0.9728
Sutterella	0.1921	0.0590	0.3616	0.0000	10.0637	0.3841	0.0120	0.7652	0.0000	20.5265	0.9726	0.9728
Odoribacter	0.4480	0.0528	0.9793	0.0123	20.8551	0.5762	0.1461	0.9484	0.0000	30.2922	0.9728	0.9728
Anaerofilum	0.0000	0.0000	0.0000	0.0000	0.0000	0.0000	0.0000	0.0000	0.0000	0.0000	NA	NA
Anaeroplasma	0.0000	0.0000	0.0000	0.0000	0.0000	0.0000	0.0000	0.0000	0.0000	0.0000	NA	NA
Anderseniella	0.0000	0.0000	0.0000	0.0000	0.0000	0.0000	0.0000	0.0000	0.0000	0.0000	NA	NA
Asteroleplasma	0.0000	0.0000	0.0000	0.0000	0.0000	0.0000	0.0000	0.0000	0.0000	0.0000	NA	NA
Atopobium	0.0000	0.0000	0.0000	0.0000	0.0000	0.0000	0.0000	0.0000	0.0000	0.0000	NA	NA
Burkholderia	0.0000	0.0000	0.0000	0.0000	0.0000	0.0000	0.0000	0.0000	0.0000	0.0000	NA	NA
Butyricicoccus	0.0000	0.0000	0.0000	0.0000	0.0000	0.0000	0.0000	0.0000	0.0000	0.0000	NA	NA
Capnocytophaga	0.0000	0.0000	0.0000	0.0000	0.0000	0.0000	0.0000	0.0000	0.0000	0.0000	NA	NA
Chloroplast transformation vector pL3-PrrnG10L-CT1	0.0000	0.0000	0.0000	0.0000	0.0000	0.0000	0.0000	0.0000	0.0000	0.0000	NA	NA
Chorispora bungeana	0.0000	0.0000	0.0000	0.0000	0.0000	0.0000	0.0000	0.0000	0.0000	0.0000	NA	NA
Cloacibacillus	0.0000	0.0000	0.0000	0.0000	0.0000	0.0000	0.0000	0.0000	0.0000	0.0000	NA	NA
Cocos nucifera (coconut palm)	0.0000	0.0000	0.0000	0.0000	0.0000	0.0000	0.0000	0.0000	0.0000	0.0000	NA	NA
Coprobacillus	0.0000	0.0000	0.0000	0.0000	0.0000	0.0000	0.0000	0.0000	0.0000	0.0000	NA	NA
Eubacterium	0.0000	0.0000	0.0000	0.0000	0.0000	0.0000	0.0000	0.0000	0.0000	0.0000	NA	NA
Fraxinus excelsior (European ash)	0.0000	0.0000	0.0000	0.0000	0.0000	0.0000	0.0000	0.0000	0.0000	0.0000	NA	NA
Gemella	0.0000	0.0000	0.0000	0.0000	0.0000	0.0000	0.0000	0.0000	0.0000	0.0000	NA	NA
Helianthus giganteus	0.0000	0.0000	0.0000	0.0000	0.0000	0.0000	0.0000	0.0000	0.0000	0.0000	NA	NA
Hydrogenoanaerobacterium	0.0000	0.0000	0.0000	0.0000	0.0000	0.0000	0.0000	0.0000	0.0000	0.0000	NA	NA
Olsenella	0.0000	0.0000	0.0000	0.0000	0.0000	0.0000	0.0000	0.0000	0.0000	0.0000	NA	NA
Oscillibacter	0.0000	0.0000	0.0000	0.0000	0.0000	0.0000	0.0000	0.0000	0.0000	0.0000	NA	NA
Oscillospira	0.0000	0.0000	0.0000	0.0000	0.0000	0.0000	0.0000	0.0000	0.0000	0.0000	NA	NA
Owenweeksia	0.0000	0.0000	0.0000	0.0000	0.0000	0.0000	0.0000	0.0000	0.0000	0.0000	NA	NA
Peptostreptococcus	0.0000	0.0000	0.0000	0.0000	0.0000	0.0000	0.0000	0.0000	0.0000	0.0000	NA	NA
Phoenix dactylifera (date palm)	0.0000	0.0000	0.0000	0.0000	0.0000	0.0000	0.0000	0.0000	0.0000	0.0000	NA	NA
Portulaca oleracea	0.0000	0.0000	0.0000	0.0000	0.0000	0.0000	0.0000	0.0000	0.0000	0.0000	NA	NA
Pyramidobacter	0.0000	0.0000	0.0000	0.0000	0.0000	0.0000	0.0000	0.0000	0.0000	0.0000	NA	NA
Rikenella	0.0000	0.0000	0.0000	0.0000	0.0000	0.0000	0.0000	0.0000	0.0000	0.0000	NA	NA
Rothia	0.0000	0.0000	0.0000	0.0000	0.0000	0.0000	0.0000	0.0000	0.0000	0.0000	NA	NA
Saccharofermentans	0.0000	0.0000	0.0000	0.0000	0.0000	0.0000	0.0000	0.0000	0.0000	0.0000	NA	NA
Shuttleworthia	0.0000	0.0000	0.0000	0.0000	0.0000	0.0000	0.0000	0.0000	0.0000	0.0000	NA	NA
Solanum tuberosum (potato)	0.0000	0.0000	0.0000	0.0000	0.0000	0.0000	0.0000	0.0000	0.0000	0.0000	NA	NA
Succiniclasticum	0.0000	0.0000	0.0000	0.0000	0.0000	0.0000	0.0000	0.0000	0.0000	0.0000	NA	NA
Synergistes	0.0000	0.0000	0.0000	0.0000	0.0000	0.0000	0.0000	0.0000	0.0000	0.0000	NA	NA
Syngonanthus chrysanthus	0.0000	0.0000	0.0000	0.0000	0.0000	0.0000	0.0000	0.0000	0.0000	0.0000	NA	NA
Treponema	0.0000	0.0000	0.0000	0.0000	0.0000	0.0000	0.0000	0.0000	0.0000	0.0000	NA	NA
Uncultured Alkaliflexus sp	0.0000	0.0000	0.0000	0.0000	0.0000	0.0000	0.0000	0.0000	0.0000	0.0000	NA	NA

Uncultured Bacteroidetes bacterium-02	0.0000	0.0000	0.0000	0.0000	0.0000	0.0000	0.0000	0.0000	0.0000	0.0000	NA	NA
Uncultured bacterium-01	0.0000	0.0000	0.0000	0.0000	0.0000	0.0000	0.0000	0.0000	0.0000	0.0000	NA	NA
Uncultured bacterium-04	0.0000	0.0000	0.0000	0.0000	0.0000	0.0000	0.0000	0.0000	0.0000	0.0000	NA	NA
Uncultured bacterium-06	0.0000	0.0000	0.0000	0.0000	0.0000	0.0000	0.0000	0.0000	0.0000	0.0000	NA	NA
Uncultured bacterium-10	0.0000	0.0000	0.0000	0.0000	0.0000	0.0000	0.0000	0.0000	0.0000	0.0000	NA	NA
Uncultured bacterium-11	0.0000	0.0000	0.0000	0.0000	0.0000	0.0000	0.0000	0.0000	0.0000	0.0000	NA	NA
Uncultured bacterium-12	0.0000	0.0000	0.0000	0.0000	0.0000	0.0000	0.0000	0.0000	0.0000	0.0000	NA	NA
Uncultured organism-01	0.0000	0.0000	0.0000	0.0000	0.0000	0.0000	0.0000	0.0000	0.0000	0.0000	NA	NA
Uncultured organism-03	0.0000	0.0000	0.0000	0.0000	0.0000	0.0000	0.0000	0.0000	0.0000	0.0000	NA	NA
Uncultured organism-04	0.0000	0.0000	0.0000	0.0000	0.0000	0.0000	0.0000	0.0000	0.0000	0.0000	NA	NA
Uncultured rumen bacterium-02	0.0000	0.0000	0.0000	0.0000	0.0000	0.0000	0.0000	0.0000	0.0000	0.0000	NA	NA
Uncultured-17	0.0000	0.0000	0.0000	0.0000	0.0000	0.0000	0.0000	0.0000	0.0000	0.0000	NA	NA
Uncultured-18	0.0000	0.0000	0.0000	0.0000	0.0000	0.0000	0.0000	0.0000	0.0000	0.0000	NA	NA
Uncultured-19	0.0000	0.0000	0.0000	0.0000	0.0000	0.0000	0.0000	0.0000	0.0000	0.0000	NA	NA
{Unknown Genus} Porphyromonadaceae	0.0000	0.0000	0.0000	0.0000	0.0000	0.0000	0.0000	0.0000	0.0000	0.0000	NA	NA

SD—standard deviation; NA—not available; *p*-value—calculated with the non-parametric Mann–Whitney test; *p*-adj.—*p* values adjusted with Benjamini–Hochberg FDR for multiple comparisons; red font—statistically significant results ($p < 0.05$)

Table S6. The percentage abundance of the intestinal microbiota in the study group of COVID-19 patients and healthy controls at the species level.

Species	COVID-19 patients, $n = 8$					Controls, $n = 14$					*p*-value	*p*- adj.
	Mean	Median	SD	Min.	Max.	Mean	Median	SD	Min.	Max.		
Uncultured Bacteroidetes bacterium-001	0.8836	0.1605	10.8511	0.0000	50.3916	0.0072	0.0000	0.0191	0.0000	0.0697	**0.0010**	0.4264
Uncultured bacterium-159	0.7296	0.1971	10.3843	0.0092	40.1029	0.0238	0.0160	0.0286	0.0000	0.0923	**0.0027**	0.5558
Metagenome	0.0113	0.0083	0.0129	0.0000	0.0365	0.0009	0.0000	0.0034	0.0000	0.0125	**0.0070**	0.6525
Uncultured Lachnospiraceae bacterium	0.6735	0.4051	10.0333	0.0280	30.1628	0.0958	0.0177	0.1255	0.0000	0.3447	**0.0085**	0.6525
Uncultured bacterium-070	90.0130	70.5285	40.4249	20.2477	150.7559	30.6931	30.0242	20.4242	0.2031	70.9860	**0.0086**	0.6525
Uncultured bacterium-069	20.3393	20.8579	10.3308	0.6382	40.1642	0.9252	0.6275	0.8493	0.0543	20.8568	**0.0105**	0.6525
Uncultured organism-029	0.4308	0.2736	0.5467	0.0364	10.7276	0.0951	0.0484	0.1149	0.0000	0.3754	**0.0126**	0.6525
Uncultured organism-025	0.8141	0.7307	0.5710	0.2609	10.8823	0.3304	0.2089	0.3503	0.0322	10.3519	**0.0127**	0.6525
Uncultured Erysipelotrichaceae bacterium-002	0.0831	0.0504	0.0943	0.0000	0.2247	0.0023	0.0000	0.0050	0.0000	0.0154	**0.0175**	0.6704
Uncultured organism-027	0.0543	0.0144	0.0719	0.0000	0.1813	0.0063	0.0000	0.0141	0.0000	0.0439	**0.0189**	0.6704
Uncultured bacterium-120	0.0281	0.0000	0.0747	0.0000	0.2127	0.0000	0.0000	0.0000	0.0000	0.0000	**0.0191**	0.6704
Uncultured organism-024	0.0747	0.0786	0.0674	0.0000	0.1594	0.0115	0.0000	0.0202	0.0000	0.0650	**0.0216**	0.6704
Parabacteroides distasonis	0.0315	0.0112	0.0435	0.0000	0.1079	0.0024	0.0000	0.0089	0.0000	0.0335	**0.0289**	0.6704
Uncultured bacterium-033	0.3251	0.1953	0.4565	0.0364	10.4389	0.0888	0.0314	0.1356	0.0000	0.3998	**0.0314**	0.6704
Uncultured bacterium-139	0.0122	0.0018	0.0296	0.0000	0.0854	0.0010	0.0000	0.0039	0.0000	0.0144	**0.0365**	0.6704
Uncultured bacterium-057	0.5871	0.5478	0.4037	0.0728	10.3289	0.2884	0.1021	0.4319	0.0119	10.3609	**0.0374**	0.6704
Uncultured organism-036	0.3859	0.2865	0.2600	0.1597	0.9994	0.8421	0.7487	0.5702	0.0080	10.7918	**0.0374**	0.6704
Bilophila wadsworthia 3_1_6	0.0211	0.0125	0.0279	0.0000	0.0838	0.0044	0.0000	0.0089	0.0000	0.0251	**0.0404**	0.6704
Uncultured bacterium-006	0.0311	0.0035	0.0445	0.0000	0.1109	0.0019	0.0000	0.0058	0.0000	0.0217	0.0504	0.6704
Eubacterium hallii DSM 3353	0.2026	0.1759	0.2223	0.0035	0.6670	0.0580	0.0148	0.0850	0.0000	0.2422	0.0504	0.6704
Uncultured bacterium-094	0.4804	0.4065	0.3781	0.0785	10.3589	0.2595	0.1913	0.2426	0.0098	0.8306	0.0518	0.6704
Uncultured bacterium-011	0.0187	0.0060	0.0389	0.0000	0.1139	0.0017	0.0000	0.0038	0.0000	0.0125	0.0574	0.6704
Uncultured bacterium-075	110.2305	10.7596	40.0247	50.1146	160.2072	80.0466	60.6156	50.7639	20.9551	250.2438	0.0605	0.6704
Bacterium NLAE-zl-P241	0.0138	0.0000	0.0352	0.0000	0.1005	0.0000	0.0000	0.0000	0.0000	0.0000	0.0648	0.6704
Enterobacter hormaechei-002	0.0043	0.0000	0.0084	0.0000	0.0223	0.0000	0.0000	0.0000	0.0000	0.0000	0.0648	0.6704
Gordonibacter pamelaeae	0.0008	0.0000	0.0016	0.0000	0.0041	0.0000	0.0000	0.0000	0.0000	0.0000	0.0648	0.6704
Uncultured Adlercreutzia sp	0.0011	0.0000	0.0021	0.0000	0.0048	0.0000	0.0000	0.0000	0.0000	0.0000	0.0648	0.6704
Uncultured bacterium-051	0.0020	0.0000	0.0043	0.0000	0.0122	0.0000	0.0000	0.0000	0.0000	0.0000	0.0648	0.6704
Uncultured bacterium-131	0.0054	0.0000	0.0128	0.0000	0.0366	0.0000	0.0000	0.0000	0.0000	0.0000	0.0648	0.6704
Uncultured bacterium-142	0.0107	0.0000	0.0202	0.0000	0.0494	0.0000	0.0000	0.0000	0.0000	0.0000	0.0648	0.6704

{Unknown Family} Bacteroidales	0.0016	0.0000	0.0037	0.0000	0.0106	0.0000	0.0000	0.0000	0.0000	0.0000	0.0648	0.6704
Uncultured Firmicutes bacterium-002	0.0086	0.0000	0.0124	0.0000	0.0280	0.0003	0.0000	0.0011	0.0000	0.0043	0.0679	0.6704
Uncultured bacterium-039	0.4477	0.3193	0.4770	0.0652	10.5312	0.1956	0.0473	0.2737	0.0000	0.8772	0.0697	0.6704
Uncultured organism-028	0.1535	0.0453	0.2154	0.0092	0.5177	0.0456	0.0122	0.0792	0.0000	0.2941	0.0697	0.6704
Uncultured bacterium-140	0.0000	0.0000	0.0000	0.0000	0.0000	0.0150	0.0000	0.0377	0.0000	0.1412	0.0699	0.6704
Uncultured bacterium-110	0.1487	0.1088	0.1666	0.0000	0.4580	0.0338	0.0026	0.0960	0.0000	0.3646	0.0715	0.6704
Uncultured bacterium-017	10.2400	0.9065	10.2340	0.0112	20.8641	0.6259	0.0415	0.9207	0.0000	20.6966	0.0788	0.6704
Human gut metagenome-010	0.1541	0.0970	0.1486	0.0141	0.3947	0.0622	0.0252	0.0806	0.0000	0.2409	0.0814	0.6704
Uncultured bacterium-086	0.2494	0.0313	0.4029	0.0035	0.9499	0.5829	0.2827	0.8441	0.0038	30.0474	0.0818	0.6704
Uncultured bacterium-025	0.4095	0.0240	0.9653	0.0000	20.7796	0.1011	0.0000	0.3611	0.0000	10.3552	0.0997	0.6704
Uncultured bacterium-024	0.0000	0.0000	0.0000	0.0000	0.0000	0.5155	0.0000	10.3388	0.0000	50.0191	0.1159	0.6704
Uncultured bacterium-148	0.0000	0.0000	0.0000	0.0000	0.0000	0.0028	0.0000	0.0052	0.0000	0.0158	0.1159	0.6704
Uncultured organism-011	0.0000	0.0000	0.0000	0.0000	0.0000	20.5753	0.0000	60.0614	0.0000	190.0372	0.1159	0.6704
Bacterium enrichment culture clone Ecwsrb007	0.4388	0.0139	10.1727	0.0000	30.3399	0.0415	0.0000	0.1405	0.0000	0.5289	0.1165	0.6704
Uncultured organism-042	0.1351	0.0077	0.3516	0.0000	10.0047	0.0481	0.0279	0.0405	0.0000	0.1058	0.1225	0.6704
Clostridium spiroforme DSM 1552	0.0020	0.0000	0.0033	0.0000	0.0092	0.0011	0.0000	0.0041	0.0000	0.0155	0.1282	0.6704
Uncultured Firmicutes bacterium-006	0.0014	0.0000	0.0020	0.0000	0.0046	0.0011	0.0000	0.0041	0.0000	0.0155	0.1282	0.6704
Uncultured bacterium-076	0.0736	0.0520	0.0725	0.0073	0.2127	0.0814	0.0077	0.1601	0.0000	0.5560	0.1411	0.6704
Uncultured organism-001	0.0529	0.0000	0.0886	0.0000	0.2466	0.0048	0.0000	0.0122	0.0000	0.0339	0.1497	0.6704
Flavonifractor plautii	0.0049	0.0028	0.0057	0.0000	0.0142	0.0025	0.0000	0.0055	0.0000	0.0152	0.1541	0.6704
Uncultured organism-020	0.0262	0.0218	0.0210	0.0000	0.0637	0.0145	0.0063	0.0190	0.0000	0.0619	0.1619	0.6704
Uncultured bacterium-113	0.0660	0.0140	0.1101	0.0000	0.3084	0.0476	0.0000	0.1087	0.0000	0.3486	0.1681	0.6704
Human gut metagenome-001	0.0847	0.0165	0.1605	0.0000	0.4705	0.3320	0.1039	0.5410	0.0000	10.8863	0.1807	0.6704
Uncultured bacterium-073	0.2340	0.2009	0.1399	0.0392	0.4599	0.3082	0.0872	0.5179	0.0121	10.6048	0.1832	0.6704
Acidaminococcus intestini	0.0000	0.0000	0.0000	0.0000	0.0000	0.0297	0.0000	0.0876	0.0000	0.3275	0.1885	0.6704
Bifidobacterium ruminantium	0.0000	0.0000	0.0000	0.0000	0.0000	0.1352	0.0000	0.4501	0.0000	10.6918	0.1885	0.6704
Rumen bacterium 2/9293-18	0.0000	0.0000	0.0000	0.0000	0.0000	0.0124	0.0000	0.0433	0.0000	0.1625	0.1885	0.6704
Uncultured Bacteroidetes bacterium-003	0.0000	0.0000	0.0000	0.0000	0.0000	0.0017	0.0000	0.0038	0.0000	0.0125	0.1885	0.6704
Uncultured bacterium-118	0.0000	0.0000	0.0000	0.0000	0.0000	0.0018	0.0000	0.0043	0.0000	0.0152	0.1885	0.6704
Uncultured organism-004	0.0035	0.0016	0.0060	0.0000	0.0176	0.0364	0.0083	0.0869	0.0000	0.3298	0.1910	0.6704
Uncultured Christensenellaceae bacterium	0.0137	0.0000	0.0387	0.0000	0.1094	0.0643	0.0000	0.1570	0.0000	0.5934	0.2008	0.6704
Butyrate-producing bacterium PH07BW09	0.0082	0.0028	0.0148	0.0000	0.0439	0.0035	0.0000	0.0074	0.0000	0.0223	0.2064	0.6704

Uncultured Streptococcus sp	0.1259	0.0000	0.3395	0.0000	0.9656	0.0017	0.0000	0.0043	0.0000	0.0125	0.2096	0.6704
Uncultured bacterium-135	0.0154	0.0087	0.0181	0.0000	0.0490	0.3989	0.0000	10.3258	0.0000	40.9796	0.2188	0.6704
Anaerostipes sp. 1y-2	0.0003	0.0000	0.0008	0.0000	0.0024	0.0000	0.0000	0.0000	0.0000	0.0000	0.2193	0.6704
Bacterium NLAE-zl-H512	0.0008	0.0000	0.0023	0.0000	0.0064	0.0000	0.0000	0.0000	0.0000	0.0000	0.2193	0.6704
Bacterium YE57	0.0003	0.0000	0.0009	0.0000	0.0025	0.0000	0.0000	0.0000	0.0000	0.0000	0.2193	0.6704
Bacteroides sp. R6	0.0003	0.0000	0.0009	0.0000	0.0025	0.0000	0.0000	0.0000	0.0000	0.0000	0.2193	0.6704
Bifidobacterium kashiwanohense	0.0012	0.0000	0.0034	0.0000	0.0096	0.0000	0.0000	0.0000	0.0000	0.0000	0.2193	0.6704
Bifidobacterium saeculare	0.0035	0.0000	0.0098	0.0000	0.0277	0.0000	0.0000	0.0000	0.0000	0.0000	0.2193	0.6704
Clostridiales bacterium DJF_B152	0.0012	0.0000	0.0034	0.0000	0.0097	0.0000	0.0000	0.0000	0.0000	0.0000	0.2193	0.6704
Clostridiales bacterium canine oral taxon 219	0.0005	0.0000	0.0014	0.0000	0.0041	0.0000	0.0000	0.0000	0.0000	0.0000	0.2193	0.6704
Clostridium sp	0.0004	0.0000	0.0011	0.0000	0.0032	0.0000	0.0000	0.0000	0.0000	0.0000	0.2193	0.6704
Clostridium sp. TM-40	0.0115	0.0000	0.0327	0.0000	0.0924	0.0000	0.0000	0.0000	0.0000	0.0000	0.2193	0.6704
Eggerthella lenta DSM 2243	0.0010	0.0000	0.0029	0.0000	0.0081	0.0000	0.0000	0.0000	0.0000	0.0000	0.2193	0.6704
Enterobacter ludwigii	0.0005	0.0000	0.0014	0.0000	0.0041	0.0000	0.0000	0.0000	0.0000	0.0000	0.2193	0.6704
Enterobacter sp. E177e	0.0005	0.0000	0.0014	0.0000	0.0041	0.0000	0.0000	0.0000	0.0000	0.0000	0.2193	0.6704
Enterococcus durans	0.0041	0.0000	0.0115	0.0000	0.0325	0.0000	0.0000	0.0000	0.0000	0.0000	0.2193	0.6704
Enterococcus faecium	0.0020	0.0000	0.0058	0.0000	0.0163	0.0000	0.0000	0.0000	0.0000	0.0000	0.2193	0.6704
Enterococcus lactis	0.0030	0.0000	0.0086	0.0000	0.0244	0.0000	0.0000	0.0000	0.0000	0.0000	0.2193	0.6704
Enterococcus sp. CGLBL186	0.0114	0.0000	0.0323	0.0000	0.0915	0.0000	0.0000	0.0000	0.0000	0.0000	0.2193	0.6704
Enterococcus sp. M1T7B1	0.0005	0.0000	0.0014	0.0000	0.0041	0.0000	0.0000	0.0000	0.0000	0.0000	0.2193	0.6704
Eubacterium fissicatena	0.0018	0.0000	0.0051	0.0000	0.0143	0.0000	0.0000	0.0000	0.0000	0.0000	0.2193	0.6704
Eubacterium sp. 3_1_31	0.0006	0.0000	0.0016	0.0000	0.0046	0.0000	0.0000	0.0000	0.0000	0.0000	0.2193	0.6704
Human gut metagenome-013	0.0104	0.0000	0.0293	0.0000	0.0829	0.0000	0.0000	0.0000	0.0000	0.0000	0.2193	0.6704
Leuconostoc mesenteroides	0.0015	0.0000	0.0043	0.0000	0.0122	0.0000	0.0000	0.0000	0.0000	0.0000	0.2193	0.6704
Leuconostoc mesenteroides subsp. mesenteroides	0.0005	0.0000	0.0014	0.0000	0.0041	0.0000	0.0000	0.0000	0.0000	0.0000	0.2193	0.6704
Parabacteroides goldsteinii dnLKV18	0.0008	0.0000	0.0023	0.0000	0.0065	0.0000	0.0000	0.0000	0.0000	0.0000	0.2193	0.6704
Providencia sp. enrichment culture clone SRC_NBB4	0.0005	0.0000	0.0014	0.0000	0.0041	0.0000	0.0000	0.0000	0.0000	0.0000	0.2193	0.6704
Streptococcus suis SC84	0.0009	0.0000	0.0025	0.0000	0.0071	0.0000	0.0000	0.0000	0.0000	0.0000	0.2193	0.6704
Trueperella pyogenes	0.0005	0.0000	0.0014	0.0000	0.0039	0.0000	0.0000	0.0000	0.0000	0.0000	0.2193	0.6704
Uncultured Actinomycetales bacterium	0.0015	0.0000	0.0042	0.0000	0.0118	0.0000	0.0000	0.0000	0.0000	0.0000	0.2193	0.6704
Uncultured Arcanobacterium sp	0.0005	0.0000	0.0014	0.0000	0.0039	0.0000	0.0000	0.0000	0.0000	0.0000	0.2193	0.6704
Uncultured Bacillales bacterium	0.0062	0.0000	0.0174	0.0000	0.0494	0.0000	0.0000	0.0000	0.0000	0.0000	0.2193	0.6704
Uncultured Bacteroidales bacterium-002	0.0007	0.0000	0.0020	0.0000	0.0056	0.0000	0.0000	0.0000	0.0000	0.0000	0.2193	0.6704
Uncultured Bacteroidetes bacterium-002	0.0009	0.0000	0.0025	0.0000	0.0071	0.0000	0.0000	0.0000	0.0000	0.0000	0.2193	0.6704
Uncultured Desulfovibrio sp	0.0056	0.0000	0.0158	0.0000	0.0448	0.0000	0.0000	0.0000	0.0000	0.0000	0.2193	0.6704
Uncultured Firmicutes bacterium-008	0.0018	0.0000	0.0050	0.0000	0.0141	0.0000	0.0000	0.0000	0.0000	0.0000	0.2193	0.6704
Uncultured Klebsiella sp-002	0.0006	0.0000	0.0017	0.0000	0.0049	0.0000	0.0000	0.0000	0.0000	0.0000	0.2193	0.6704
Uncultured Ruminococcus sp	0.0019	0.0000	0.0052	0.0000	0.0148	0.0000	0.0000	0.0000	0.0000	0.0000	0.2193	0.6704

Uncultured Shigella sp-002	0.0216	0.0000	0.0611	0.0000	0.1728	0.0000	0.0000	0.0000	0.0000	0.0000	0.2193	0.6704
Uncultured bacterium-003	0.0040	0.0000	0.0113	0.0000	0.0319	0.0000	0.0000	0.0000	0.0000	0.0000	0.2193	0.6704
Uncultured bacterium-007	0.0018	0.0000	0.0051	0.0000	0.0143	0.0000	0.0000	0.0000	0.0000	0.0000	0.2193	0.6704
Uncultured bacterium-021	0.0003	0.0000	0.0008	0.0000	0.0024	0.0000	0.0000	0.0000	0.0000	0.0000	0.2193	0.6704
Uncultured bacterium-035	0.0004	0.0000	0.0012	0.0000	0.0035	0.0000	0.0000	0.0000	0.0000	0.0000	0.2193	0.6704
Uncultured bacterium-043	0.0006	0.0000	0.0017	0.0000	0.0049	0.0000	0.0000	0.0000	0.0000	0.0000	0.2193	0.6704
Uncultured bacterium-049	0.0006	0.0000	0.0017	0.0000	0.0049	0.0000	0.0000	0.0000	0.0000	0.0000	0.2193	0.6704
Uncultured bacterium-052	0.0054	0.0000	0.0152	0.0000	0.0430	0.0000	0.0000	0.0000	0.0000	0.0000	0.2193	0.6704
Uncultured bacterium-065	0.0012	0.0000	0.0035	0.0000	0.0098	0.0000	0.0000	0.0000	0.0000	0.0000	0.2193	0.6704
Uncultured bacterium-077	0.0004	0.0000	0.0011	0.0000	0.0032	0.0000	0.0000	0.0000	0.0000	0.0000	0.2193	0.6704
Uncultured bacterium-085	0.0009	0.0000	0.0025	0.0000	0.0071	0.0000	0.0000	0.0000	0.0000	0.0000	0.2193	0.6704
Uncultured bacterium-121	0.0004	0.0000	0.0011	0.0000	0.0032	0.0000	0.0000	0.0000	0.0000	0.0000	0.2193	0.6704
Uncultured bacterium-126	0.0004	0.0000	0.0011	0.0000	0.0032	0.0000	0.0000	0.0000	0.0000	0.0000	0.2193	0.6704
Uncultured marine bacterium-002	0.0005	0.0000	0.0014	0.0000	0.0041	0.0000	0.0000	0.0000	0.0000	0.0000	0.2193	0.6704
Uncultured organism-017	0.0008	0.0000	0.0023	0.0000	0.0065	0.0000	0.0000	0.0000	0.0000	0.0000	0.2193	0.6704
Uncultured organism-033	0.0005	0.0000	0.0014	0.0000	0.0041	0.0000	0.0000	0.0000	0.0000	0.0000	0.2193	0.6704
Uncultured organism-038	0.0004	0.0000	0.0011	0.0000	0.0032	0.0000	0.0000	0.0000	0.0000	0.0000	0.2193	0.6704
Uncultured rumen bacterium 4C28d-12	0.0003	0.0000	0.0009	0.0000	0.0025	0.0000	0.0000	0.0000	0.0000	0.0000	0.2193	0.6704
Uncultured rumen bacterium-015	0.0004	0.0000	0.0012	0.0000	0.0035	0.0000	0.0000	0.0000	0.0000	0.0000	0.2193	0.6704
Unidentified-002	0.0003	0.0000	0.0009	0.0000	0.0025	0.0000	0.0000	0.0000	0.0000	0.0000	0.2193	0.6704
Unidentified-004	0.0004	0.0000	0.0012	0.0000	0.0035	0.0000	0.0000	0.0000	0.0000	0.0000	0.2193	0.6704
Unidentified-006	0.0006	0.0000	0.0016	0.0000	0.0046	0.0000	0.0000	0.0000	0.0000	0.0000	0.2193	0.6704
Unidentified-008	0.0003	0.0000	0.0009	0.0000	0.0025	0.0000	0.0000	0.0000	0.0000	0.0000	0.2193	0.6704
Unidentified-009	0.0004	0.0000	0.0012	0.0000	0.0035	0.0000	0.0000	0.0000	0.0000	0.0000	0.2193	0.6704
{Unknown Class} Bacteroidetes	0.0014	0.0000	0.0039	0.0000	0.0111	0.0000	0.0000	0.0000	0.0000	0.0000	0.2193	0.6704
{Unknown Phylum} Bacteria	0.0010	0.0000	0.0028	0.0000	0.0078	0.0000	0.0000	0.0000	0.0000	0.0000	0.2193	0.6704
{Unknown Species} Arcanobacterium	0.0005	0.0000	0.0014	0.0000	0.0039	0.0000	0.0000	0.0000	0.0000	0.0000	0.2193	0.6704
{Unknown Species} Bacteroides	0.0003	0.0000	0.0008	0.0000	0.0024	0.0000	0.0000	0.0000	0.0000	0.0000	0.2193	0.6704
{Unknown Species} Bifidobacterium	0.0004	0.0000	0.0011	0.0000	0.0032	0.0000	0.0000	0.0000	0.0000	0.0000	0.2193	0.6704
Uncultured bacterium-027	0.0232	0.0000	0.0632	0.0000	0.1795	0.8770	0.0000	10.9006	0.0000	60.7999	0.2197	0.6704
Uncultured bacterium-010	0.0022	0.0000	0.0041	0.0000	0.0102	0.0003	0.0000	0.0010	0.0000	0.0038	0.2299	0.6704
Uncultured Firmicutes bacterium-003	0.3041	0.1308	0.3777	0.0159	0.9954	0.5656	0.3050	0.6845	0.0000	20.5667	0.2323	0.6704
Uncultured bacterium-080	20.8897	20.4284	10.7053	10.5017	60.6169	20.0262	10.7490	10.7909	0.0000	60.5916	0.2323	0.6704
Uncultured alpha proteobacterium-001	0.0171	0.0000	0.0247	0.0000	0.0610	0.0014	0.0000	0.0029	0.0000	0.0077	0.2583	0.6704

Faecalibacterium cf0. prausnitzii KLE1255	0.1612	0.0528	0.2144	0.0041	0.5751	0.2954	0.1389	0.3969	0.0000	10.4741	0.2601	0.6704
Uncultured organism-045	0.4300	0.2459	0.4716	0.0346	10.2541	0.7979	0.4368	0.9862	0.0505	30.6191	0.2601	0.6704
Uncultured bacterium-133	0.3132	0.0352	0.5436	0.0000	10.4108	0.1510	0.0000	0.3522	0.0000	10.1572	0.2636	0.6704
Uncultured bacterium-078	0.0053	0.0000	0.0128	0.0000	0.0366	0.0468	0.0049	0.1465	0.0000	0.5547	0.2669	0.6704
Bacteroidetes bacterium Eg28	0.0023	0.0000	0.0054	0.0000	0.0153	0.0009	0.0000	0.0035	0.0000	0.0132	0.2774	0.6704
Uncultured Enterobacteriaceae bacterium-002	0.1640	0.0000	0.4627	0.0000	10.3091	0.0024	0.0000	0.0091	0.0000	0.0342	0.2774	0.6704
Uncultured bacterium-130	0.0330	0.0000	0.0918	0.0000	0.2602	0.0096	0.0000	0.0358	0.0000	0.1338	0.2774	0.6704
Uncultured bacterium-058	0.0166	0.0000	0.0429	0.0000	0.1226	0.0020	0.0000	0.0051	0.0000	0.0150	0.2851	0.6704
Uncultured organism-023	0.0015	0.0000	0.0021	0.0000	0.0049	0.0006	0.0000	0.0014	0.0000	0.0040	0.2851	0.6704
Uncultured organism-007	0.0216	0.0125	0.0187	0.0000	0.0546	0.0970	0.0470	0.1200	0.0000	0.3580	0.2874	0.6704
Uncultured organism-035	0.5475	0.4842	0.5976	0.0353	10.8558	0.3152	0.2192	0.3619	0.0000	10.3922	0.2900	0.6704
Human gut metagenome-007	0.0325	0.0060	0.0498	0.0000	0.1434	0.0476	0.0019	0.1355	0.0000	0.5139	0.2942	0.6704
Campylobacter jejuni RM1221	0.0000	0.0000	0.0000	0.0000	0.0000	0.1163	0.0000	0.4179	0.0000	10.5671	0.3050	0.6704
Human gut metagenome-002	0.0000	0.0000	0.0000	0.0000	0.0000	0.1900	0.0000	0.6636	0.0000	20.4901	0.3050	0.6704
Uncultured Clostridia bacterium	0.0000	0.0000	0.0000	0.0000	0.0000	0.0026	0.0000	0.0072	0.0000	0.0251	0.3050	0.6704
Uncultured Clostridiales bacterium-002	0.0000	0.0000	0.0000	0.0000	0.0000	0.0008	0.0000	0.0022	0.0000	0.0075	0.3050	0.6704
Uncultured Dialister sp	0.0000	0.0000	0.0000	0.0000	0.0000	0.0102	0.0000	0.0342	0.0000	0.1283	0.3050	0.6704
Uncultured bacterium-044	0.0000	0.0000	0.0000	0.0000	0.0000	0.0134	0.0000	0.0343	0.0000	0.1047	0.3050	0.6704
Uncultured bacterium-071	0.0000	0.0000	0.0000	0.0000	0.0000	0.0119	0.0000	0.0399	0.0000	0.1495	0.3050	0.6704
Uncultured bacterium-101	0.0000	0.0000	0.0000	0.0000	0.0000	0.0007	0.0000	0.0017	0.0000	0.0052	0.3050	0.6704
Uncultured bacterium-112	0.0000	0.0000	0.0000	0.0000	0.0000	0.0065	0.0000	0.0164	0.0000	0.0457	0.3050	0.6704
Uncultured bacterium-123	0.0000	0.0000	0.0000	0.0000	0.0000	0.0020	0.0000	0.0062	0.0000	0.0231	0.3050	0.6704
Uncultured bacterium-149	0.0000	0.0000	0.0000	0.0000	0.0000	0.0011	0.0000	0.0028	0.0000	0.0080	0.3050	0.6704
Uncultured organism-014	0.0000	0.0000	0.0000	0.0000	0.0000	0.0022	0.0000	0.0058	0.0000	0.0192	0.3050	0.6704
Uncultured organism-049	0.0000	0.0000	0.0000	0.0000	0.0000	0.1701	0.0000	0.4601	0.0000	10.5918	0.3050	0.6704
Uncultured organism-054	0.0000	0.0000	0.0000	0.0000	0.0000	0.0111	0.0000	0.0406	0.0000	0.1523	0.3050	0.6704
Uncultured rumen bacterium-006	0.0000	0.0000	0.0000	0.0000	0.0000	0.0035	0.0000	0.0111	0.0000	0.0415	0.3050	0.6704
Uncultured sediment bacterium	0.0000	0.0000	0.0000	0.0000	0.0000	0.0006	0.0000	0.0014	0.0000	0.0040	0.3050	0.6704
Unidentified rumen bacterium RFN10	0.0000	0.0000	0.0000	0.0000	0.0000	0.0008	0.0000	0.0022	0.0000	0.0077	0.3050	0.6704
Uncultured bacterium-116	0.1787	0.0849	0.2126	0.0000	0.5467	0.0960	0.0141	0.1863	0.0000	0.6748	0.3106	0.6704
Uncultured bacterium-079	20.9143	10.6751	30.1695	0.3323	90.4000	20.3719	10.0539	30.6412	0.0442	130.2267	0.3223	0.6704
Bacterium NLAE-zl-G195	0.0010	0.0000	0.0029	0.0000	0.0081	0.1052	0.0000	0.2483	0.0000	0.8160	0.3291	0.6704

Lachnospira multipara	0.0004	0.0000	0.0012	0.0000	0.0035	0.0364	0.0000	0.0752	0.0000	0.2203	0.3291	0.6704
Uncultured organism-059	0.0006	0.0000	0.0017	0.0000	0.0049	0.0407	0.0000	0.0773	0.0000	0.2440	0.3291	0.6704
Uncultured Firmicutes bacterium-004	0.0048	0.0000	0.0093	0.0000	0.0245	0.0080	0.0000	0.0299	0.0000	0.1119	0.3311	0.6704
Uncultured bacterium-117	0.0020	0.0000	0.0045	0.0000	0.0129	0.0136	0.0000	0.0511	0.0000	0.1910	0.3311	0.6704
{Unknown Genus} Lachnospiraceae	0.0175	0.0012	0.0424	0.0000	0.1220	0.0531	0.0000	0.1732	0.0000	0.6498	0.3425	0.6704
Uncultured bacterium-026	0.3459	0.2671	0.3994	0.0000	10.2214	10.5365	0.9849	20.0375	0.0000	70.3397	0.3563	0.6704
Uncultured organism-003	0.6478	0.5173	0.6837	0.0000	10.9246	20.9672	10.5160	30.6858	0.0000	10.3173	0.3563	0.6704
{Unknown Family} Clostridiales	0.0021	0.0000	0.0029	0.0000	0.0065	0.0030	0.0000	0.0092	0.0000	0.0342	0.3772	0.6704
Uncultured organism-058	0.0831	0.0034	0.1517	0.0000	0.3649	10.0257	0.0000	20.1091	0.0000	60.3184	0.3785	0.6704
Uncultured bacterium-108	0.2401	0.0537	0.4394	0.0000	10.2774	0.1581	0.0058	0.3684	0.0000	10.3621	0.3820	0.6704
Uncultured bacterium-066	0.0260	0.0147	0.0316	0.0032	0.0980	0.0306	0.0094	0.0545	0.0000	0.2011	0.3888	0.6704
Uncultured bacterium-001	0.5878	0.1369	10.0007	0.0000	20.8928	0.8647	0.2052	0.9398	0.0038	20.4378	0.3934	0.6704
Uncultured bacterium-022	0.2786	0.1083	0.3769	0.0139	10.1175	0.1641	0.0953	0.1884	0.0000	0.5505	0.3934	0.6704
Uncultured bacterium-056	0.3810	0.1780	0.4665	0.0183	10.3715	0.7155	0.4924	0.9403	0.0000	30.6547	0.3934	0.6704
Uncultured bacterium-028	0.7710	0.8046	0.4127	0.1372	10.4613	20.2817	10.3187	30.1771	0.0152	110.1256	0.3936	0.6704
Alistipes timonensis JC136	0.0037	0.0000	0.0104	0.0000	0.0294	0.0530	0.0000	0.1291	0.0000	0.3741	0.4295	0.6704
Uncultured organism-006	0.0095	0.0094	0.0062	0.0000	0.0203	0.0977	0.0317	0.1375	0.0000	0.4796	0.4299	0.6704
Uncultured bacterium-031	0.2615	0.1707	0.2158	0.0366	0.6101	0.2418	0.0549	0.2731	0.0098	0.8638	0.4325	0.6704
Uncultured organism-039	0.0178	0.0020	0.0243	0.0000	0.0533	0.0355	0.0000	0.0741	0.0000	0.2053	0.4330	0.6704
Uncultured bacterium-053	0.0341	0.0000	0.0907	0.0000	0.2582	0.0008	0.0000	0.0022	0.0000	0.0076	0.4468	0.6704
Uncultured bacterium-104	0.0737	0.0025	0.1943	0.0000	0.5542	0.0120	0.0000	0.0258	0.0000	0.0815	0.4519	0.6704
Uncultured bacterium-018	0.0062	0.0063	0.0054	0.0000	0.0161	0.0263	0.0120	0.0359	0.0000	0.1161	0.4628	0.6704
Uncultured organism-012	0.0848	0.0403	0.1142	0.0224	0.3613	0.1159	0.0291	0.1728	0.0000	0.5772	0.4731	0.6704
Uncultured bacterium-023	0.8507	0.8665	0.6191	0.0797	10.6699	0.7472	0.4651	0.8537	0.0000	20.6009	0.4736	0.6704
Uncultured bacterium-054	0.3857	0.0599	0.8466	0.0046	20.4617	0.0495	0.0229	0.0659	0.0000	0.2346	0.4736	0.6704
Uncultured Clostridiaceae bacterium-001	0.0567	0.0029	0.1480	0.0000	0.4227	0.0693	0.0106	0.1855	0.0000	0.7057	0.4844	0.6704
Uncultured organism-018	0.0012	0.0000	0.0035	0.0000	0.0099	0.0018	0.0000	0.0037	0.0000	0.0132	0.4857	0.6704
Clostridium sp. MDA2315	0.0078	0.0000	0.0142	0.0000	0.0371	0.0205	0.0028	0.0432	0.0000	0.1600	0.5023	0.6704
Uncultured bacterium-004	0.0167	0.0040	0.0328	0.0000	0.0952	0.0283	0.0000	0.0751	0.0000	0.2780	0.5023	0.6704
Anaerostipes butyraticus	0.0000	0.0000	0.0000	0.0000	0.0000	0.0138	0.0000	0.0517	0.0000	0.1935	0.5083	0.6704
Bacillus sp. SC156(2010)	0.0000	0.0000	0.0000	0.0000	0.0000	0.0003	0.0000	0.0011	0.0000	0.0040	0.5083	0.6704
Bacterium NLAE-zl-G202	0.0000	0.0000	0.0000	0.0000	0.0000	0.0012	0.0000	0.0045	0.0000	0.0167	0.5083	0.6704
Bacterium mpn-isolate group 4	0.0000	0.0000	0.0000	0.0000	0.0000	0.0077	0.0000	0.0289	0.0000	0.1080	0.5083	0.6704

Bacteroides finegoldii CL09T03C10	0.0000	0.0000	0.0000	0.0000	0.0000	0.0225	0.0000	0.0841	0.0000	0.3146	0.5083	0.6704
Bacteroides fragilis CL03T00C08	0.0000	0.0000	0.0000	0.0000	0.0000	0.1954	0.0000	0.7309	0.0000	20.7349	0.5083	0.6704
Bacteroides sp. AP1	0.0000	0.0000	0.0000	0.0000	0.0000	0.0005	0.0000	0.0019	0.0000	0.0072	0.5083	0.6704
Bifidobacterium scardovii	0.0000	0.0000	0.0000	0.0000	0.0000	0.0004	0.0000	0.0014	0.0000	0.0052	0.5083	0.6704
Campylobacter hominis	0.0000	0.0000	0.0000	0.0000	0.0000	0.0005	0.0000	0.0019	0.0000	0.0072	0.5083	0.6704
Campylobacter hominis ATCC BAA-381	0.0000	0.0000	0.0000	0.0000	0.0000	0.0028	0.0000	0.0106	0.0000	0.0397	0.5083	0.6704
Campylobacter upsaliensis	0.0000	0.0000	0.0000	0.0000	0.0000	0.0454	0.0000	0.1699	0.0000	0.6358	0.5083	0.6704
Campylobacter upsaliensis RM3195	0.0000	0.0000	0.0000	0.0000	0.0000	0.0556	0.0000	0.2079	0.0000	0.7780	0.5083	0.6704
Carnobacterium maltaromaticum ATCC 35586	0.0000	0.0000	0.0000	0.0000	0.0000	0.0003	0.0000	0.0010	0.0000	0.0038	0.5083	0.6704
Citrobacter freundii-001	0.0000	0.0000	0.0000	0.0000	0.0000	0.8901	0.0000	30.3304	0.0000	120.4613	0.5083	0.6704
Clostridiaceae bacterium WN011	0.0000	0.0000	0.0000	0.0000	0.0000	0.0066	0.0000	0.0249	0.0000	0.0930	0.5083	0.6704
Clostridiales bacterium 27-5-10	0.0000	0.0000	0.0000	0.0000	0.0000	0.0005	0.0000	0.0019	0.0000	0.0072	0.5083	0.6704
Clostridiales bacterium VE202-08	0.0000	0.0000	0.0000	0.0000	0.0000	0.0003	0.0000	0.0011	0.0000	0.0040	0.5083	0.6704
Clostridium clostridioforme 90A4	0.0000	0.0000	0.0000	0.0000	0.0000	0.0004	0.0000	0.0014	0.0000	0.0052	0.5083	0.6704
Clostridium methoxybenzovorans SR3	0.0000	0.0000	0.0000	0.0000	0.0000	0.0031	0.0000	0.0117	0.0000	0.0439	0.5083	0.6704
Clostridium nexile	0.0000	0.0000	0.0000	0.0000	0.0000	0.0041	0.0000	0.0153	0.0000	0.0571	0.5083	0.6704
Clostridium sp. ID6	0.0000	0.0000	0.0000	0.0000	0.0000	0.0044	0.0000	0.0163	0.0000	0.0609	0.5083	0.6704
Clostridium sp. SL-2013-71	0.0000	0.0000	0.0000	0.0000	0.0000	0.0004	0.0000	0.0014	0.0000	0.0052	0.5083	0.6704
Clostridium sp. U201	0.0000	0.0000	0.0000	0.0000	0.0000	0.0035	0.0000	0.0131	0.0000	0.0490	0.5083	0.6704
Coriobacteriaceae bacterium JC110	0.0000	0.0000	0.0000	0.0000	0.0000	0.0003	0.0000	0.0010	0.0000	0.0038	0.5083	0.6704
Dialister sp. oral taxon 502	0.0000	0.0000	0.0000	0.0000	0.0000	0.0008	0.0000	0.0030	0.0000	0.0112	0.5083	0.6704
Enterobacter hormaechei-001	0.0000	0.0000	0.0000	0.0000	0.0000	0.0003	0.0000	0.0011	0.0000	0.0040	0.5083	0.6704
Enterobacter sp. JN0513	0.0000	0.0000	0.0000	0.0000	0.0000	0.4848	0.0000	10.8138	0.0000	60.7866	0.5083	0.6704
Enterobacter sp. NAB3	0.0000	0.0000	0.0000	0.0000	0.0000	0.0006	0.0000	0.0022	0.0000	0.0080	0.5083	0.6704
Enterobacter sp. R4-368	0.0000	0.0000	0.0000	0.0000	0.0000	0.0028	0.0000	0.0106	0.0000	0.0397	0.5083	0.6704
Enterococcus faecalis	0.0000	0.0000	0.0000	0.0000	0.0000	0.0005	0.0000	0.0019	0.0000	0.0072	0.5083	0.6704
Erysipelotrichaceae bacterium 21_3	0.0000	0.0000	0.0000	0.0000	0.0000	0.0031	0.0000	0.0117	0.0000	0.0439	0.5083	0.6704
Eubacteriaceae bacterium DJF_VR85	0.0000	0.0000	0.0000	0.0000	0.0000	0.0009	0.0000	0.0034	0.0000	0.0125	0.5083	0.6704
Holdemania massiliensis AP2	0.0000	0.0000	0.0000	0.0000	0.0000	0.0004	0.0000	0.0014	0.0000	0.0052	0.5083	0.6704
Human gut metagenome-006	0.0000	0.0000	0.0000	0.0000	0.0000	0.0004	0.0000	0.0014	0.0000	0.0052	0.5083	0.6704
Human gut metagenome-016	0.0000	0.0000	0.0000	0.0000	0.0000	0.0065	0.0000	0.0244	0.0000	0.0914	0.5083	0.6704
Klebsiella michiganensis	0.0000	0.0000	0.0000	0.0000	0.0000	0.0010	0.0000	0.0039	0.0000	0.0144	0.5083	0.6704
Klebsiella oxytoca	0.0000	0.0000	0.0000	0.0000	0.0000	0.0010	0.0000	0.0039	0.0000	0.0144	0.5083	0.6704
Klebsiella sp. enrichment culture clone CL108	0.0000	0.0000	0.0000	0.0000	0.0000	0.1283	0.0000	0.4800	0.0000	10.7961	0.5083	0.6704
Lachnospiraceae bacterium DJF_RR13	0.0000	0.0000	0.0000	0.0000	0.0000	0.0051	0.0000	0.0190	0.0000	0.0712	0.5083	0.6704
Lactobacillus acidophilus	0.0000	0.0000	0.0000	0.0000	0.0000	0.0129	0.0000	0.0483	0.0000	0.1806	0.5083	0.6704
Lactobacillus plantarum	0.0000	0.0000	0.0000	0.0000	0.0000	0.0011	0.0000	0.0041	0.0000	0.0155	0.5083	0.6704
Methanogenic archaeon CH1270	0.0000	0.0000	0.0000	0.0000	0.0000	0.0003	0.0000	0.0011	0.0000	0.0040	0.5083	0.6704
Methylobacterium MAP-2	0.0000	0.0000	0.0000	0.0000	0.0000	0.0005	0.0000	0.0020	0.0000	0.0076	0.5083	0.6704

Paenibacillus sp. 6M20	0.0000	0.0000	0.0000	0.0000	0.0000	0.0025	0.0000	0.0092	0.0000	0.0345	0.5083	0.6704
Paenibacillus sp. enrichment culture clone ts0224R	0.0000	0.0000	0.0000	0.0000	0.0000	0.0003	0.0000	0.0010	0.0000	0.0038	0.5083	0.6704
Pediococcus lolii	0.0000	0.0000	0.0000	0.0000	0.0000	0.0011	0.0000	0.0041	0.0000	0.0155	0.5083	0.6704
Prevotella disiens FB035-09AN	0.0000	0.0000	0.0000	0.0000	0.0000	0.0003	0.0000	0.0010	0.0000	0.0038	0.5083	0.6704
Prevotella marshii	0.0000	0.0000	0.0000	0.0000	0.0000	0.0383	0.0000	0.1435	0.0000	0.5368	0.5083	0.6704
Pseudomonas guineae	0.0000	0.0000	0.0000	0.0000	0.0000	0.2673	0.0000	10.0000	0.0000	30.7418	0.5083	0.6704
Pseudomonas lundensis	0.0000	0.0000	0.0000	0.0000	0.0000	0.0021	0.0000	0.0077	0.0000	0.0288	0.5083	0.6704
Pseudomonas sp. enrichment culture clone IC1-46	0.0000	0.0000	0.0000	0.0000	0.0000	0.0014	0.0000	0.0051	0.0000	0.0192	0.5083	0.6704
Pseudomonas syringae	0.0000	0.0000	0.0000	0.0000	0.0000	0.0008	0.0000	0.0031	0.0000	0.0115	0.5083	0.6704
Pseudomonas syringae pv0. tomato	0.0000	0.0000	0.0000	0.0000	0.0000	0.0005	0.0000	0.0020	0.0000	0.0077	0.5083	0.6704
Ruminococcus gnavus CC55_001C	0.0000	0.0000	0.0000	0.0000	0.0000	0.0083	0.0000	0.0310	0.0000	0.1161	0.5083	0.6704
Ruminococcus sp. DJF_VR67	0.0000	0.0000	0.0000	0.0000	0.0000	0.0012	0.0000	0.0044	0.0000	0.0166	0.5083	0.6704
Sphingomonas soli	0.0000	0.0000	0.0000	0.0000	0.0000	0.0005	0.0000	0.0020	0.0000	0.0076	0.5083	0.6704
Sporosarcina psychrophila	0.0000	0.0000	0.0000	0.0000	0.0000	0.0009	0.0000	0.0032	0.0000	0.0121	0.5083	0.6704
Uncultured Enterobacteriaceae bacterium-001	0.0000	0.0000	0.0000	0.0000	0.0000	0.0003	0.0000	0.0011	0.0000	0.0040	0.5083	0.6704
Uncultured Firmicutes bacterium-001	0.0000	0.0000	0.0000	0.0000	0.0000	0.0136	0.0000	0.0507	0.0000	0.1898	0.5083	0.6704
Uncultured Klebsiella sp-001	0.0000	0.0000	0.0000	0.0000	0.0000	0.4856	0.0000	10.8170	0.0000	60.7987	0.5083	0.6704
Uncultured Lactobacillus sp.	0.0000	0.0000	0.0000	0.0000	0.0000	0.0024	0.0000	0.0090	0.0000	0.0335	0.5083	0.6704
Uncultured Mollicutes bacterium	0.0000	0.0000	0.0000	0.0000	0.0000	0.0194	0.0000	0.0725	0.0000	0.2713	0.5083	0.6704
Uncultured Oxalobacteraceae bacterium	0.0000	0.0000	0.0000	0.0000	0.0000	0.0003	0.0000	0.0011	0.0000	0.0040	0.5083	0.6704
Uncultured archaeon-001	0.0000	0.0000	0.0000	0.0000	0.0000	0.0009	0.0000	0.0034	0.0000	0.0125	0.5083	0.6704
Uncultured archaeon-002	0.0000	0.0000	0.0000	0.0000	0.0000	0.0009	0.0000	0.0034	0.0000	0.0125	0.5083	0.6704
Uncultured bacterium adhufec350.25	0.0000	0.0000	0.0000	0.0000	0.0000	0.0003	0.0000	0.0010	0.0000	0.0038	0.5083	0.6704
Uncultured bacterium-032	0.0000	0.0000	0.0000	0.0000	0.0000	0.0046	0.0000	0.0171	0.0000	0.0642	0.5083	0.6704
Uncultured bacterium-036	0.0000	0.0000	0.0000	0.0000	0.0000	0.0005	0.0000	0.0020	0.0000	0.0076	0.5083	0.6704
Uncultured bacterium-037	0.0000	0.0000	0.0000	0.0000	0.0000	0.0005	0.0000	0.0020	0.0000	0.0076	0.5083	0.6704
Uncultured bacterium-040	0.0000	0.0000	0.0000	0.0000	0.0000	0.0050	0.0000	0.0186	0.0000	0.0697	0.5083	0.6704
Uncultured bacterium-045	0.0000	0.0000	0.0000	0.0000	0.0000	0.0016	0.0000	0.0061	0.0000	0.0230	0.5083	0.6704
Uncultured bacterium-048	0.0000	0.0000	0.0000	0.0000	0.0000	0.1596	0.0000	0.5974	0.0000	20.2351	0.5083	0.6704
Uncultured bacterium-050	0.0000	0.0000	0.0000	0.0000	0.0000	0.0276	0.0000	0.1034	0.0000	0.3870	0.5083	0.6704
Uncultured bacterium-063	0.0000	0.0000	0.0000	0.0000	0.0000	0.0005	0.0000	0.0019	0.0000	0.0072	0.5083	0.6704
Uncultured bacterium-067	0.0000	0.0000	0.0000	0.0000	0.0000	0.0014	0.0000	0.0053	0.0000	0.0198	0.5083	0.6704
Uncultured bacterium-074	0.0000	0.0000	0.0000	0.0000	0.0000	0.0003	0.0000	0.0010	0.0000	0.0038	0.5083	0.6704
Uncultured bacterium-095	0.0000	0.0000	0.0000	0.0000	0.0000	0.0012	0.0000	0.0044	0.0000	0.0166	0.5083	0.6704

Uncultured bacterium-109	0.0000	0.0000	0.0000	0.0000	0.0000	0.0233	0.0000	0.0871	0.0000	0.3259	0.5083	0.6704
Uncultured bacterium-119	0.0000	0.0000	0.0000	0.0000	0.0000	0.0003	0.0000	0.0011	0.0000	0.0040	0.5083	0.6704
Uncultured bacterium-122	0.0000	0.0000	0.0000	0.0000	0.0000	0.0028	0.0000	0.0106	0.0000	0.0396	0.5083	0.6704
Uncultured bacterium-128	0.0000	0.0000	0.0000	0.0000	0.0000	0.0008	0.0000	0.0032	0.0000	0.0119	0.5083	0.6704
Uncultured bacterium-129	0.0000	0.0000	0.0000	0.0000	0.0000	0.0005	0.0000	0.0020	0.0000	0.0076	0.5083	0.6704
Uncultured bacterium-138	0.0000	0.0000	0.0000	0.0000	0.0000	0.0011	0.0000	0.0041	0.0000	0.0153	0.5083	0.6704
Uncultured bacterium-141	0.0000	0.0000	0.0000	0.0000	0.0000	0.0088	0.0000	0.0328	0.0000	0.1227	0.5083	0.6704
Uncultured bacterium-143	0.0000	0.0000	0.0000	0.0000	0.0000	0.5380	0.0000	20.0132	0.0000	70.5327	0.5083	0.6704
Uncultured bacterium-150	0.0000	0.0000	0.0000	0.0000	0.0000	0.4131	0.0000	10.5456	0.0000	50.7832	0.5083	0.6704
Uncultured bacterium-152	0.0000	0.0000	0.0000	0.0000	0.0000	0.0003	0.0000	0.0011	0.0000	0.0043	0.5083	0.6704
Uncultured bacterium-156	0.0000	0.0000	0.0000	0.0000	0.0000	0.0061	0.0000	0.0227	0.0000	0.0851	0.5083	0.6704
Uncultured beta proteobacterium	0.0000	0.0000	0.0000	0.0000	0.0000	0.0012	0.0000	0.0044	0.0000	0.0166	0.5083	0.6704
Uncultured gamma proteobacterium	0.0000	0.0000	0.0000	0.0000	0.0000	0.0080	0.0000	0.0299	0.0000	0.1119	0.5083	0.6704
Uncultured organism-009	0.0000	0.0000	0.0000	0.0000	0.0000	0.0961	0.0000	0.3596	0.0000	10.3455	0.5083	0.6704
Uncultured organism-022	0.0000	0.0000	0.0000	0.0000	0.0000	0.0059	0.0000	0.0220	0.0000	0.0824	0.5083	0.6704
Uncultured organism-026	0.0000	0.0000	0.0000	0.0000	0.0000	0.0819	0.0000	0.3065	0.0000	10.1468	0.5083	0.6704
Uncultured organism-056	0.0000	0.0000	0.0000	0.0000	0.0000	0.0005	0.0000	0.0020	0.0000	0.0076	0.5083	0.6704
Uncultured organism-057	0.0000	0.0000	0.0000	0.0000	0.0000	0.0011	0.0000	0.0041	0.0000	0.0153	0.5083	0.6704
Uncultured organism-061	0.0000	0.0000	0.0000	0.0000	0.0000	0.0011	0.0000	0.0042	0.0000	0.0158	0.5083	0.6704
Uncultured organism-064	0.0000	0.0000	0.0000	0.0000	0.0000	0.0003	0.0000	0.0010	0.0000	0.0038	0.5083	0.6704
Uncultured organism-069	0.0000	0.0000	0.0000	0.0000	0.0000	0.0003	0.0000	0.0010	0.0000	0.0038	0.5083	0.6704
Uncultured rumen bacterium-002	0.0000	0.0000	0.0000	0.0000	0.0000	0.0089	0.0000	0.0333	0.0000	0.1246	0.5083	0.6704
Uncultured rumen bacterium-007	0.0000	0.0000	0.0000	0.0000	0.0000	0.1177	0.0000	0.4405	0.0000	10.6481	0.5083	0.6704
Uncultured rumen bacterium-010	0.0000	0.0000	0.0000	0.0000	0.0000	0.0008	0.0000	0.0030	0.0000	0.0113	0.5083	0.6704
Uncultured rumen bacterium-011	0.0000	0.0000	0.0000	0.0000	0.0000	0.0006	0.0000	0.0021	0.0000	0.0079	0.5083	0.6704
Uncultured rumen bacterium-014	0.0000	0.0000	0.0000	0.0000	0.0000	0.0160	0.0000	0.0599	0.0000	0.2243	0.5083	0.6704
Unidentified-007	0.0000	0.0000	0.0000	0.0000	0.0000	0.0003	0.0000	0.0011	0.0000	0.0040	0.5083	0.6704
Virgibacillus soli	0.0000	0.0000	0.0000	0.0000	0.0000	0.0005	0.0000	0.0020	0.0000	0.0077	0.5083	0.6704
Wallaby gut metagenome	0.0000	0.0000	0.0000	0.0000	0.0000	0.0006	0.0000	0.0021	0.0000	0.0079	0.5083	0.6704
{Unknown Genus} Peptostreptococcaceae	0.0000	0.0000	0.0000	0.0000	0.0000	0.0009	0.0000	0.0034	0.0000	0.0125	0.5083	0.6704
{Unknown Species} Blautia	0.0000	0.0000	0.0000	0.0000	0.0000	0.0068	0.0000	0.0254	0.0000	0.0950	0.5083	0.6704
{Unknown Species} Coprobacter	0.0000	0.0000	0.0000	0.0000	0.0000	0.0003	0.0000	0.0011	0.0000	0.0040	0.5083	0.6704
{Unknown Species} Incertae Sedis	0.0000	0.0000	0.0000	0.0000	0.0000	0.0225	0.0000	0.0841	0.0000	0.3148	0.5083	0.6704

{Unknown Species} Uncultured-001	0.0000	0.0000	0.0000	0.0000	0.0000	0.0003	0.0000	0.0010	0.0000	0.0038	0.5083	0.6704
{Unknown Species} Uncultured-002	0.0000	0.0000	0.0000	0.0000	0.0000	0.0003	0.0000	0.0011	0.0000	0.0040	0.5083	0.6704
Uncultured Clostridium sp	0.0038	0.0000	0.0087	0.0000	0.0249	0.0015	0.0000	0.0044	0.0000	0.0161	0.5097	0.6704
Uncultured bacterium-093	0.0047	0.0000	0.0114	0.0000	0.0325	0.0017	0.0000	0.0053	0.0000	0.0197	0.5097	0.6704
Uncultured organism-051	0.0530	0.0199	0.0831	0.0000	0.2470	0.0274	0.0064	0.0416	0.0000	0.1225	0.5125	0.6704
Bacterium NLAE-zl-C532	0.0219	0.0020	0.0537	0.0000	0.1546	0.0080	0.0000	0.0209	0.0000	0.0791	0.5151	0.6704
Uncultured bacterium-014	0.0113	0.0023	0.0147	0.0000	0.0353	0.0069	0.0000	0.0121	0.0000	0.0397	0.5151	0.6704
Uncultured bacterium-009	0.3248	0.1851	0.3303	0.0670	0.9825	0.3910	0.2771	0.3589	0.0000	10.2784	0.5167	0.6704
Uncultured bacterium-015	140.2845	140.6608	10.7147	20.3581	30.1222	110.4793	90.3120	80.8311	0.1884	30.4562	0.5167	0.6704
Uncultured bacterium-111	0.4611	0.4592	0.3652	0.0046	0.9576	0.3325	0.2469	0.2687	0.0000	0.7653	0.5167	0.6704
Uncultured organism-046	0.1808	0.1676	0.1510	0.0056	0.5097	0.6413	0.2194	10.0366	0.0000	30.2475	0.5167	0.6704
Uncultured bacterium-020	0.3965	0.0355	0.9951	0.0000	20.8551	0.4785	0.0933	0.8634	0.0000	30.0755	0.5389	0.6912
Uncultured organism-005	0.0195	0.0028	0.0335	0.0000	0.0874	0.0292	0.0143	0.0427	0.0000	0.1425	0.5424	0.6912
Uncultured Bifidobacterium sp. 9A	0.0007	0.0000	0.0020	0.0000	0.0056	0.0196	0.0000	0.0443	0.0000	0.1316	0.5428	0.6912
Uncultured bacterium-013	0.0004	0.0000	0.0011	0.0000	0.0032	0.0327	0.0000	0.1150	0.0000	0.4319	0.5428	0.6912
Uncultured bacterium-029	0.0020	0.0000	0.0058	0.0000	0.0163	0.0121	0.0000	0.0252	0.0000	0.0704	0.5428	0.6912
Uncultured bacterium-103	0.0008	0.0000	0.0023	0.0000	0.0064	0.0269	0.0000	0.0687	0.0000	0.2308	0.5428	0.6912
Uncultured bacterium-090	0.1390	0.0290	0.1919	0.0000	0.5182	0.1973	0.0038	0.3905	0.0000	10.2528	0.5479	0.6955
Uncultured organism-032	0.0276	0.0000	0.0641	0.0000	0.1848	0.0823	0.0019	0.1605	0.0000	0.5078	0.5510	0.6972
Human gut metagenome-008	0.0660	0.0151	0.0887	0.0000	0.2231	0.0755	0.0383	0.1121	0.0000	0.4089	0.5555	0.7008
Uncultured bacterium-088	0.9984	0.1637	20.2564	0.0035	60.5612	0.7676	0.2340	10.3810	0.0000	50.1667	0.5617	0.7064
Ruminococcus bromii L2-63	0.0471	0.0000	0.0839	0.0000	0.2325	0.2481	0.0000	0.4252	0.0000	10.1189	0.5658	0.7094
Uncultured Faecalibacterium sp	0.0039	0.0000	0.0098	0.0000	0.0280	0.0009	0.0000	0.0024	0.0000	0.0075	0.5770	0.7168
Uncultured bacterium-064	0.0015	0.0000	0.0029	0.0000	0.0075	0.0029	0.0000	0.0096	0.0000	0.0361	0.5770	0.7168
Uncultured bacterium-144	0.0315	0.0000	0.0877	0.0000	0.2486	0.0086	0.0000	0.0310	0.0000	0.1161	0.5770	0.7168
Uncultured bacterium-107	0.0140	0.0000	0.0373	0.0000	0.1060	0.7929	0.0000	20.2213	0.0000	70.9589	0.5916	0.7306
Uncultured bacterium-145	0.2272	0.0000	0.6302	0.0000	10.7868	0.2017	0.0000	0.5245	0.0000	10.7386	0.5916	0.7306
Fusicatenibacter saccharivorans	0.0586	0.0117	0.1027	0.0000	0.2940	0.0513	0.0364	0.0607	0.0000	0.2310	0.6067	0.7470
Uncultured bacterium-092	60.2430	50.5057	40.5705	0.4452	120.3443	70.0643	60.0154	40.9734	0.0103	150.6916	0.6087	0.7472
Gut metagenome-001	0.0009	0.0000	0.0018	0.0000	0.0049	0.0095	0.0000	0.0196	0.0000	0.0653	0.6325	0.7740
Uncultured bacterium-115	0.0123	0.0000	0.0332	0.0000	0.0943	0.0089	0.0000	0.0304	0.0000	0.1142	0.6481	0.7906
Uncultured rumen bacterium-012	0.0182	0.0000	0.0506	0.0000	0.1434	0.0226	0.0000	0.0414	0.0000	0.1247	0.6499	0.7906
Bacteroides coprophilus	0.0105	0.0000	0.0296	0.0000	0.0839	0.0053	0.0000	0.0200	0.0000	0.0748	0.6816	0.8001

Bacteroides nordii CL02T12C05	0.0005	0.0000	0.0014	0.0000	0.0041	0.0003	0.0000	0.0011	0.0000	0.0040	0.6816	0.8001
Lactococcus lactis subsp. cremoris TIFN7	0.0043	0.0000	0.0122	0.0000	0.0346	0.0003	0.0000	0.0010	0.0000	0.0038	0.6816	0.8001
Lactococcus lactis subsp. lactis	0.0051	0.0000	0.0144	0.0000	0.0407	0.0004	0.0000	0.0014	0.0000	0.0052	0.6816	0.8001
Uncultured Parabacteroides sp	0.1436	0.0000	0.4061	0.0000	10.1485	0.0006	0.0000	0.0022	0.0000	0.0080	0.6816	0.8001
Uncultured Thermoanaerobacterales bacterium	0.0275	0.0000	0.0777	0.0000	0.2199	0.0005	0.0000	0.0019	0.0000	0.0072	0.6816	0.8001
Uncultured bacterium-068	0.0035	0.0000	0.0098	0.0000	0.0277	0.0003	0.0000	0.0010	0.0000	0.0038	0.6816	0.8001
Uncultured bacterium-105	0.0146	0.0000	0.0412	0.0000	0.1165	0.0063	0.0000	0.0234	0.0000	0.0876	0.6816	0.8001
Uncultured organism-019	0.0075	0.0000	0.0212	0.0000	0.0600	0.0037	0.0000	0.0138	0.0000	0.0516	0.6816	0.8001
Uncultured organism-062	0.1075	0.0000	0.3040	0.0000	0.8599	0.0089	0.0000	0.0333	0.0000	0.1247	0.6816	0.8001
Uncultured organism-067	0.0020	0.0000	0.0056	0.0000	0.0159	0.0006	0.0000	0.0022	0.0000	0.0080	0.6816	0.8001
Uncultured rumen bacterium-005	0.0006	0.0000	0.0016	0.0000	0.0046	0.0003	0.0000	0.0010	0.0000	0.0038	0.6816	0.8001
Bacteroides plebeius DSM 17135	0.0073	0.0000	0.0205	0.0000	0.0581	0.0123	0.0000	0.0263	0.0000	0.0851	0.6850	0.8001
Uncultured organism-044	0.0117	0.0000	0.0330	0.0000	0.0934	0.0167	0.0000	0.0557	0.0000	0.2091	0.6850	0.8001
Uncultured bacterium-157	0.0080	0.0000	0.0204	0.0000	0.0582	0.0807	0.0000	0.1680	0.0000	0.4700	0.6956	0.8078
Parabacteroides merdae	0.0288	0.0233	0.0312	0.0000	0.0858	0.0472	0.0094	0.1020	0.0000	0.3785	0.6974	0.8078
Uncultured organism-034	0.1721	0.0180	0.2750	0.0000	0.7714	0.0909	0.0258	0.1525	0.0000	0.5691	0.6974	0.8078
Alistipes sp. AP11	0.0015	0.0000	0.0034	0.0000	0.0096	0.0076	0.0000	0.0192	0.0000	0.0543	0.7226	0.8322
Human gut metagenome-009	0.0220	0.0000	0.0499	0.0000	0.1418	0.0689	0.0000	0.2034	0.0000	0.7459	0.7226	0.8322
Human gut metagenome-011	0.1348	0.0036	0.2442	0.0000	0.6151	0.2717	0.0386	0.7821	0.0000	20.9736	0.7266	0.8345
Bacteroides vulgatus	0.2692	0.2807	0.1974	0.0000	0.5122	0.4329	0.2146	0.5274	0.0000	10.3616	0.7303	0.8364
Uncultured organism-060	0.0345	0.0000	0.0706	0.0000	0.2043	0.1732	0.0000	0.3440	0.0000	10.0175	0.7515	0.8441
Uncultured bacterium-002	0.0403	0.0277	0.0487	0.0000	0.1468	0.1093	0.0157	0.2293	0.0000	0.8420	0.7530	0.8441
Human gut metagenome-012	0.4089	0.0770	0.7088	0.0000	20.0578	0.3685	0.0595	0.6563	0.0000	10.9417	0.7564	0.8441
Uncultured bacterium-083	70.4253	60.4643	30.5033	30.0063	130.5151	60.5093	60.4031	30.2755	10.5558	110.2253	0.7587	0.8441
Bifidobacterium bifidum NCIMB 41171	0.0167	0.0000	0.0474	0.0000	0.1340	0.0049	0.0000	0.0111	0.0000	0.0335	0.7609	0.8441
Uncultured bacterium-047	0.0191	0.0000	0.0539	0.0000	0.1525	0.0075	0.0000	0.0188	0.0000	0.0684	0.7609	0.8441
Uncultured organism-010	0.0062	0.0000	0.0175	0.0000	0.0494	0.0032	0.0000	0.0089	0.0000	0.0332	0.7609	0.8441
Human gut metagenome-005	0.2513	0.0051	0.5694	0.0000	10.6353	0.1160	0.0125	0.2525	0.0000	0.9169	0.7743	0.8441
Uncultured Ruminococcaceae bacterium	0.0271	0.0041	0.0418	0.0000	0.1181	0.0973	0.0083	0.2839	0.0000	10.0738	0.7743	0.8441
Bifidobacterium catenulatum	0.0023	0.0000	0.0064	0.0000	0.0182	0.0017	0.0000	0.0064	0.0000	0.0241	0.7844	0.8441
Butyrate-producing bacterium SM4/1	0.0015	0.0000	0.0043	0.0000	0.0123	0.0061	0.0000	0.0228	0.0000	0.0851	0.7844	0.8441
Citrobacter freundii-002	0.0030	0.0000	0.0086	0.0000	0.0244	0.4241	0.0000	10.5870	0.0000	50.9381	0.7844	0.8441
Coriobacteriaceae bacterium WAL 18889	0.0004	0.0000	0.0012	0.0000	0.0035	0.0030	0.0000	0.0111	0.0000	0.0415	0.7844	0.8441

Dorea formicigenerans ATCC 27755	0.0006	0.0000	0.0017	0.0000	0.0049	0.0012	0.0000	0.0044	0.0000	0.0166	0.7844	0.8441
Slackia sp. NATTS	0.0004	0.0000	0.0011	0.0000	0.0032	0.0166	0.0000	0.0622	0.0000	0.2326	0.7844	0.8441
Uncultured Bacteroidales bacterium-001	0.0213	0.0000	0.0602	0.0000	0.1704	0.0741	0.0000	0.2772	0.0000	10.0370	0.7844	0.8441
Uncultured Clostridiales bacterium-004	0.0305	0.0000	0.0862	0.0000	0.2438	0.0209	0.0000	0.0783	0.0000	0.2928	0.7844	0.8441
Uncultured bacterium-055	0.0004	0.0000	0.0011	0.0000	0.0032	0.0006	0.0000	0.0022	0.0000	0.0080	0.7844	0.8441
Uncultured bacterium-084	0.0004	0.0000	0.0011	0.0000	0.0032	0.0003	0.0000	0.0010	0.0000	0.0038	0.7844	0.8441
Uncultured bacterium-125	0.0003	0.0000	0.0008	0.0000	0.0024	0.0009	0.0000	0.0034	0.0000	0.0125	0.7844	0.8441
Uncultured bacterium-147	0.0005	0.0000	0.0014	0.0000	0.0041	0.4491	0.0000	10.6805	0.0000	60.2880	0.7844	0.8441
Uncultured organism-068	0.0013	0.0000	0.0037	0.0000	0.0106	0.0095	0.0000	0.0354	0.0000	0.1326	0.7844	0.8441
{Unknown Class} Firmicutes	0.0004	0.0000	0.0011	0.0000	0.0032	0.0003	0.0000	0.0011	0.0000	0.0043	0.7844	0.8441
Escherichia coli	30.4217	0.0023	90.5343	0.0000	270.0160	0.2326	0.0000	0.7372	0.0000	20.7790	0.7942	0.8524
Bacteroides ovatus CL02T12C04	0.0515	0.0091	0.1240	0.0000	0.3580	0.2456	0.0137	0.5138	0.0000	10.8153	0.8082	0.8652
Uncultured organism-016	0.0127	0.0000	0.0336	0.0000	0.0956	0.0058	0.0000	0.0145	0.0000	0.0490	0.8163	0.8715
Human gut metagenome-014	0.1444	0.0616	0.1775	0.0000	0.5041	0.4010	0.0386	0.8761	0.0000	30.1561	0.8638	0.9065
Uncultured organism-040	0.1457	0.1015	0.1317	0.0000	0.3396	0.1600	0.0777	0.1957	0.0000	0.6063	0.8638	0.9065
Uncultured bacterium-151	0.0009	0.0000	0.0025	0.0000	0.0071	0.0165	0.0000	0.0559	0.0000	0.2097	0.8638	0.9065
Uncultured bacterium-072	0.3698	0.3256	0.1996	0.0756	0.6552	0.4351	0.3437	0.4565	0.0000	10.4642	0.8645	0.9065
Uncultured bacterium-102	30.5033	30.3674	10.5187	10.4416	60.3305	40.4838	20.7213	40.5730	0.3298	150.1725	0.8645	0.9065
Uncultured organism-002	40.4816	40.1118	30.8750	0.6607	10.6480	60.7463	40.4352	70.5390	0.0166	230.7757	0.8645	0.9065
Uncultured organism-031	0.9982	0.9631	0.6206	0.2015	10.8315	10.5140	0.7286	20.0064	0.0583	70.2688	0.8645	0.9065
Uncultured bacterium-061	0.0087	0.0048	0.0136	0.0000	0.0405	0.0100	0.0071	0.0118	0.0000	0.0344	0.9156	0.9573
Uncultured bacterium-136	0.1572	0.0399	0.2927	0.0000	0.8593	0.2097	0.0120	0.4344	0.0000	10.5090	0.9176	0.9573
Bifidobacterium longum subsp. longum	0.1762	0.0330	0.3855	0.0000	10.1229	0.2533	0.0189	0.6268	0.0000	20.3427	0.9442	0.9728
Achromobacter xylosoxidans	0.0341	0.0000	0.0963	0.0000	0.2724	0.0238	0.0000	0.0694	0.0000	0.2534	0.9544	0.9728
Bacteroides salyersiae CL02T12C01	0.1508	0.0000	0.4264	0.0000	10.2062	0.0479	0.0000	0.1531	0.0000	0.5718	0.9544	0.9728
Blautia stercoris	0.0021	0.0000	0.0059	0.0000	0.0168	0.0020	0.0000	0.0062	0.0000	0.0231	0.9544	0.9728
Oscillospiraceae bacterium VE202-24	0.0030	0.0000	0.0086	0.0000	0.0244	0.0008	0.0000	0.0022	0.0000	0.0076	0.9544	0.9728
Uncultured bacterium-042	0.0156	0.0000	0.0442	0.0000	0.1251	0.0142	0.0000	0.0458	0.0000	0.1711	0.9544	0.9728
Uncultured bacterium-124	0.0006	0.0000	0.0017	0.0000	0.0049	0.1509	0.0000	0.5635	0.0000	20.1086	0.9544	0.9728
Uncultured bacterium-137	0.0010	0.0000	0.0029	0.0000	0.0081	0.1314	0.0000	0.4906	0.0000	10.8359	0.9544	0.9728
{Unknown Genus} Ruminococcaceae	0.0125	0.0000	0.0353	0.0000	0.0997	0.0088	0.0000	0.0318	0.0000	0.1191	0.9544	0.9728
Bacteroides sp. DSM 12148	0.0024	0.0000	0.0057	0.0000	0.0163	0.0029	0.0000	0.0071	0.0000	0.0251	0.9629	0.9728
Uncultured organism-008	0.0016	0.0000	0.0037	0.0000	0.0106	0.0038	0.0000	0.0102	0.0000	0.0377	0.9629	0.9728

Uncultured organism-053	0.0031	0.0000	0.0069	0.0000	0.0196	0.0054	0.0000	0.0118	0.0000	0.0361	0.9629	0.9728
Uncultured Turicibacter sp	0.0033	0.0020	0.0040	0.0000	0.0098	0.2184	0.0000	0.7729	0.0000	20.9023	0.9695	0.9728
Uncultured bacterium-146	10.5003	0.0000	40.1917	0.0000	110.8736	0.0731	0.0000	0.1592	0.0000	0.5510	0.9695	0.9728
Uncultured bacterium-059	0.0308	0.0074	0.0489	0.0000	0.1201	0.0531	0.0081	0.1258	0.0000	0.4322	0.9718	0.9728
Uncultured bacterium-098	40.9856	30.3406	40.5373	0.7684	140.5400	50.7569	50.4087	50.6198	0.1213	180.7126	0.9728	0.9728
Uncultured bacterium-100	0.8678	0.6873	0.6472	0.0416	20.0318	10.1223	0.7649	10.2005	0.0000	30.7037	0.9728	0.9728
Azospirillum sp. CAG:260	0.0000	0.0000	0.0000	0.0000	0.0000	0.0000	0.0000	0.0000	0.0000	0.0000	NA	NA
Bacterium ADV746	0.0000	0.0000	0.0000	0.0000	0.0000	0.0000	0.0000	0.0000	0.0000	0.0000	NA	NA
Bacterium NLAE-zl-G484	0.0000	0.0000	0.0000	0.0000	0.0000	0.0000	0.0000	0.0000	0.0000	0.0000	NA	NA
Bacterium NLAE-zl-H185	0.0000	0.0000	0.0000	0.0000	0.0000	0.0000	0.0000	0.0000	0.0000	0.0000	NA	NA
Bacterium NLAE-zl-P428	0.0000	0.0000	0.0000	0.0000	0.0000	0.0000	0.0000	0.0000	0.0000	0.0000	NA	NA
Bacterium RB5-FF-13	0.0000	0.0000	0.0000	0.0000	0.0000	0.0000	0.0000	0.0000	0.0000	0.0000	NA	NA
Bacterium ic1337	0.0000	0.0000	0.0000	0.0000	0.0000	0.0000	0.0000	0.0000	0.0000	0.0000	NA	NA
Bacterium mpn-isolate group 22	0.0000	0.0000	0.0000	0.0000	0.0000	0.0000	0.0000	0.0000	0.0000	0.0000	NA	NA
Bacterium mpn-isolate group 24	0.0000	0.0000	0.0000	0.0000	0.0000	0.0000	0.0000	0.0000	0.0000	0.0000	NA	NA
Bacteroides barnesiae	0.0000	0.0000	0.0000	0.0000	0.0000	0.0000	0.0000	0.0000	0.0000	0.0000	NA	NA
Bifidobacterium animalis subsp. lactis CECT 8145	0.0000	0.0000	0.0000	0.0000	0.0000	0.0000	0.0000	0.0000	0.0000	0.0000	NA	NA
Bifidobacterium breve	0.0000	0.0000	0.0000	0.0000	0.0000	0.0000	0.0000	0.0000	0.0000	0.0000	NA	NA
Bifidobacterium merycicum	0.0000	0.0000	0.0000	0.0000	0.0000	0.0000	0.0000	0.0000	0.0000	0.0000	NA	NA
Bifidobacterium pseudolongum subsp. globosum	0.0000	0.0000	0.0000	0.0000	0.0000	0.0000	0.0000	0.0000	0.0000	0.0000	NA	NA
Blautia hydrogenotrophica	0.0000	0.0000	0.0000	0.0000	0.0000	0.0000	0.0000	0.0000	0.0000	0.0000	NA	NA
Catenibacterium mitsuokai DSM 15897	0.0000	0.0000	0.0000	0.0000	0.0000	0.0000	0.0000	0.0000	0.0000	0.0000	NA	NA
Chloroplast transformation vector pL3-PrrnG10L-CT1	0.0000	0.0000	0.0000	0.0000	0.0000	0.0000	0.0000	0.0000	0.0000	0.0000	NA	NA
Chorispora bungeana	0.0000	0.0000	0.0000	0.0000	0.0000	0.0000	0.0000	0.0000	0.0000	0.0000	NA	NA
Clostridiaceae bacterium SL-2013-8	0.0000	0.0000	0.0000	0.0000	0.0000	0.0000	0.0000	0.0000	0.0000	0.0000	NA	NA
Clostridium carnis	0.0000	0.0000	0.0000	0.0000	0.0000	0.0000	0.0000	0.0000	0.0000	0.0000	NA	NA
Clostridium colinum	0.0000	0.0000	0.0000	0.0000	0.0000	0.0000	0.0000	0.0000	0.0000	0.0000	NA	NA
Clostridium hylemonae	0.0000	0.0000	0.0000	0.0000	0.0000	0.0000	0.0000	0.0000	0.0000	0.0000	NA	NA
Clostridium leptum-001	0.0000	0.0000	0.0000	0.0000	0.0000	0.0000	0.0000	0.0000	0.0000	0.0000	NA	NA
Clostridium leptum-002	0.0000	0.0000	0.0000	0.0000	0.0000	0.0000	0.0000	0.0000	0.0000	0.0000	NA	NA
Clostridium sp. enrichment culture clone 06-1235251-43	0.0000	0.0000	0.0000	0.0000	0.0000	0.0000	0.0000	0.0000	0.0000	0.0000	NA	NA
Clostridium sp. enrichment culture clone G-5a	0.0000	0.0000	0.0000	0.0000	0.0000	0.0000	0.0000	0.0000	0.0000	0.0000	NA	NA
Cocos nucifera (coconut palm)	0.0000	0.0000	0.0000	0.0000	0.0000	0.0000	0.0000	0.0000	0.0000	0.0000	NA	NA
Collinsella tanakaei	0.0000	0.0000	0.0000	0.0000	0.0000	0.0000	0.0000	0.0000	0.0000	0.0000	NA	NA
Coprobacillus sp. D6	0.0000	0.0000	0.0000	0.0000	0.0000	0.0000	0.0000	0.0000	0.0000	0.0000	NA	NA
Coriobacteriaceae bacterium phI	0.0000	0.0000	0.0000	0.0000	0.0000	0.0000	0.0000	0.0000	0.0000	0.0000	NA	NA
Enterobacter asburiae	0.0000	0.0000	0.0000	0.0000	0.0000	0.0000	0.0000	0.0000	0.0000	0.0000	NA	NA

Enterococcus faecium EnGen0265	0.0000	0.0000	0.0000	0.0000	0.0000	0.0000	0.0000	0.0000	0.0000	0.0000	NA	NA
Enterococcus sp. 41-2	0.0000	0.0000	0.0000	0.0000	0.0000	0.0000	0.0000	0.0000	0.0000	0.0000	NA	NA
Eubacterium sp. CS1 Van	0.0000	0.0000	0.0000	0.0000	0.0000	0.0000	0.0000	0.0000	0.0000	0.0000	NA	NA
Fraxinus excelsior (European ash)	0.0000	0.0000	0.0000	0.0000	0.0000	0.0000	0.0000	0.0000	0.0000	0.0000	NA	NA
Gut metagenome-002	0.0000	0.0000	0.0000	0.0000	0.0000	0.0000	0.0000	0.0000	0.0000	0.0000	NA	NA
Haemophilus pittmaniae	0.0000	0.0000	0.0000	0.0000	0.0000	0.0000	0.0000	0.0000	0.0000	0.0000	NA	NA
Helianthus giganteus	0.0000	0.0000	0.0000	0.0000	0.0000	0.0000	0.0000	0.0000	0.0000	0.0000	NA	NA
Holdemania filiformis DSM 12042	0.0000	0.0000	0.0000	0.0000	0.0000	0.0000	0.0000	0.0000	0.0000	0.0000	NA	NA
Human gut metagenome-003	0.0000	0.0000	0.0000	0.0000	0.0000	0.0000	0.0000	0.0000	0.0000	0.0000	NA	NA
Human gut metagenome-004	0.0000	0.0000	0.0000	0.0000	0.0000	0.0000	0.0000	0.0000	0.0000	0.0000	NA	NA
Human gut metagenome-015	0.0000	0.0000	0.0000	0.0000	0.0000	0.0000	0.0000	0.0000	0.0000	0.0000	NA	NA
Klebsiella pneumoniae	0.0000	0.0000	0.0000	0.0000	0.0000	0.0000	0.0000	0.0000	0.0000	0.0000	NA	NA
Klebsiella sp. B12	0.0000	0.0000	0.0000	0.0000	0.0000	0.0000	0.0000	0.0000	0.0000	0.0000	NA	NA
Lactobacillus paracasei	0.0000	0.0000	0.0000	0.0000	0.0000	0.0000	0.0000	0.0000	0.0000	0.0000	NA	NA
Lactobacillus rhamnosus	0.0000	0.0000	0.0000	0.0000	0.0000	0.0000	0.0000	0.0000	0.0000	0.0000	NA	NA
Lactobacillus salivarius	0.0000	0.0000	0.0000	0.0000	0.0000	0.0000	0.0000	0.0000	0.0000	0.0000	NA	NA
Lactobacillus sp. oral clone HT002	0.0000	0.0000	0.0000	0.0000	0.0000	0.0000	0.0000	0.0000	0.0000	0.0000	NA	NA
Lactococcus garvieae	0.0000	0.0000	0.0000	0.0000	0.0000	0.0000	0.0000	0.0000	0.0000	0.0000	NA	NA
Megamonas funiformis	0.0000	0.0000	0.0000	0.0000	0.0000	0.0000	0.0000	0.0000	0.0000	0.0000	NA	NA
Phoenix dactylifera (date palm)	0.0000	0.0000	0.0000	0.0000	0.0000	0.0000	0.0000	0.0000	0.0000	0.0000	NA	NA
Pluralibacter pyrinus	0.0000	0.0000	0.0000	0.0000	0.0000	0.0000	0.0000	0.0000	0.0000	0.0000	NA	NA
Portulaca oleracea	0.0000	0.0000	0.0000	0.0000	0.0000	0.0000	0.0000	0.0000	0.0000	0.0000	NA	NA
Prevotella buccae ATCC 33574	0.0000	0.0000	0.0000	0.0000	0.0000	0.0000	0.0000	0.0000	0.0000	0.0000	NA	NA
Ruminococcus champanellensis 18P13	0.0000	0.0000	0.0000	0.0000	0.0000	0.0000	0.0000	0.0000	0.0000	0.0000	NA	NA
Ruminococcus sp. 9SE51	0.0000	0.0000	0.0000	0.0000	0.0000	0.0000	0.0000	0.0000	0.0000	0.0000	NA	NA
Solanum tuberosum (potato)	0.0000	0.0000	0.0000	0.0000	0.0000	0.0000	0.0000	0.0000	0.0000	0.0000	NA	NA
Streptococcus anginosus	0.0000	0.0000	0.0000	0.0000	0.0000	0.0000	0.0000	0.0000	0.0000	0.0000	NA	NA
Streptococcus australis	0.0000	0.0000	0.0000	0.0000	0.0000	0.0000	0.0000	0.0000	0.0000	0.0000	NA	NA
Streptococcus mutans	0.0000	0.0000	0.0000	0.0000	0.0000	0.0000	0.0000	0.0000	0.0000	0.0000	NA	NA
Sutterella sanguinus	0.0000	0.0000	0.0000	0.0000	0.0000	0.0000	0.0000	0.0000	0.0000	0.0000	NA	NA
Syngonanthus chrysanthus	0.0000	0.0000	0.0000	0.0000	0.0000	0.0000	0.0000	0.0000	0.0000	0.0000	NA	NA
Uncultured Alkaliflexus sp	0.0000	0.0000	0.0000	0.0000	0.0000	0.0000	0.0000	0.0000	0.0000	0.0000	NA	NA
Uncultured Bacteroides sp	0.0000	0.0000	0.0000	0.0000	0.0000	0.0000	0.0000	0.0000	0.0000	0.0000	NA	NA
Uncultured Bacteroidetes bacterium-004	0.0000	0.0000	0.0000	0.0000	0.0000	0.0000	0.0000	0.0000	0.0000	0.0000	NA	NA
Uncultured Clostridiaceae bacterium-002	0.0000	0.0000	0.0000	0.0000	0.0000	0.0000	0.0000	0.0000	0.0000	0.0000	NA	NA
Uncultured Clostridiaceae bacterium-003	0.0000	0.0000	0.0000	0.0000	0.0000	0.0000	0.0000	0.0000	0.0000	0.0000	NA	NA
Uncultured Clostridiales bacterium-001	0.0000	0.0000	0.0000	0.0000	0.0000	0.0000	0.0000	0.0000	0.0000	0.0000	NA	NA
Uncultured Clostridiales bacterium-003	0.0000	0.0000	0.0000	0.0000	0.0000	0.0000	0.0000	0.0000	0.0000	0.0000	NA	NA
Uncultured Desulfovibrionaceae bacterium	0.0000	0.0000	0.0000	0.0000	0.0000	0.0000	0.0000	0.0000	0.0000	0.0000	NA	NA

Uncultured Erysipelotrichaceae bacterium-001	0.0000	0.0000	0.0000	0.0000	0.0000	0.0000	0.0000	0.0000	0.0000	0.0000	NA	NA
Uncultured Firmicutes bacterium-005	0.0000	0.0000	0.0000	0.0000	0.0000	0.0000	0.0000	0.0000	0.0000	0.0000	NA	NA
Uncultured Firmicutes bacterium-007	0.0000	0.0000	0.0000	0.0000	0.0000	0.0000	0.0000	0.0000	0.0000	0.0000	NA	NA
Uncultured Methanobrevibacter sp	0.0000	0.0000	0.0000	0.0000	0.0000	0.0000	0.0000	0.0000	0.0000	0.0000	NA	NA
Uncultured Oxalobacter sp	0.0000	0.0000	0.0000	0.0000	0.0000	0.0000	0.0000	0.0000	0.0000	0.0000	NA	NA
Uncultured Pantoea sp	0.0000	0.0000	0.0000	0.0000	0.0000	0.0000	0.0000	0.0000	0.0000	0.0000	NA	NA
Uncultured Prevotella sp	0.0000	0.0000	0.0000	0.0000	0.0000	0.0000	0.0000	0.0000	0.0000	0.0000	NA	NA
Uncultured Prevotellaceae bacterium	0.0000	0.0000	0.0000	0.0000	0.0000	0.0000	0.0000	0.0000	0.0000	0.0000	NA	NA
Uncultured Shigella sp-001	0.0000	0.0000	0.0000	0.0000	0.0000	0.0000	0.0000	0.0000	0.0000	0.0000	NA	NA
Uncultured Veillonellaceae bacterium	0.0000	0.0000	0.0000	0.0000	0.0000	0.0000	0.0000	0.0000	0.0000	0.0000	NA	NA
Uncultured alpha proteobacterium-002	0.0000	0.0000	0.0000	0.0000	0.0000	0.0000	0.0000	0.0000	0.0000	0.0000	NA	NA
Uncultured archaeon-003	0.0000	0.0000	0.0000	0.0000	0.0000	0.0000	0.0000	0.0000	0.0000	0.0000	NA	NA
Uncultured bacterium adhufec40	0.0000	0.0000	0.0000	0.0000	0.0000	0.0000	0.0000	0.0000	0.0000	0.0000	NA	NA
Uncultured bacterium-005	0.0000	0.0000	0.0000	0.0000	0.0000	0.0000	0.0000	0.0000	0.0000	0.0000	NA	NA
Uncultured bacterium-008	0.0000	0.0000	0.0000	0.0000	0.0000	0.0000	0.0000	0.0000	0.0000	0.0000	NA	NA
Uncultured bacterium-012	0.0000	0.0000	0.0000	0.0000	0.0000	0.0000	0.0000	0.0000	0.0000	0.0000	NA	NA
Uncultured bacterium-016	0.0000	0.0000	0.0000	0.0000	0.0000	0.0000	0.0000	0.0000	0.0000	0.0000	NA	NA
Uncultured bacterium-019	0.0000	0.0000	0.0000	0.0000	0.0000	0.0000	0.0000	0.0000	0.0000	0.0000	NA	NA
Uncultured bacterium-030	0.0000	0.0000	0.0000	0.0000	0.0000	0.0000	0.0000	0.0000	0.0000	0.0000	NA	NA
Uncultured bacterium-034	0.0000	0.0000	0.0000	0.0000	0.0000	0.0000	0.0000	0.0000	0.0000	0.0000	NA	NA
Uncultured bacterium-038	0.0000	0.0000	0.0000	0.0000	0.0000	0.0000	0.0000	0.0000	0.0000	0.0000	NA	NA
Uncultured bacterium-041	0.0000	0.0000	0.0000	0.0000	0.0000	0.0000	0.0000	0.0000	0.0000	0.0000	NA	NA
Uncultured bacterium-046	0.0000	0.0000	0.0000	0.0000	0.0000	0.0000	0.0000	0.0000	0.0000	0.0000	NA	NA
Uncultured bacterium-060	0.0000	0.0000	0.0000	0.0000	0.0000	0.0000	0.0000	0.0000	0.0000	0.0000	NA	NA
Uncultured bacterium-062	0.0000	0.0000	0.0000	0.0000	0.0000	0.0000	0.0000	0.0000	0.0000	0.0000	NA	NA
Uncultured bacterium-081	0.0000	0.0000	0.0000	0.0000	0.0000	0.0000	0.0000	0.0000	0.0000	0.0000	NA	NA
Uncultured bacterium-082	0.0000	0.0000	0.0000	0.0000	0.0000	0.0000	0.0000	0.0000	0.0000	0.0000	NA	NA
Uncultured bacterium-087	0.0000	0.0000	0.0000	0.0000	0.0000	0.0000	0.0000	0.0000	0.0000	0.0000	NA	NA
Uncultured bacterium-089	0.0000	0.0000	0.0000	0.0000	0.0000	0.0000	0.0000	0.0000	0.0000	0.0000	NA	NA
Uncultured bacterium-091	0.0000	0.0000	0.0000	0.0000	0.0000	0.0000	0.0000	0.0000	0.0000	0.0000	NA	NA
Uncultured bacterium-096	0.0000	0.0000	0.0000	0.0000	0.0000	0.0000	0.0000	0.0000	0.0000	0.0000	NA	NA
Uncultured bacterium-097	0.0000	0.0000	0.0000	0.0000	0.0000	0.0000	0.0000	0.0000	0.0000	0.0000	NA	NA
Uncultured bacterium-099	0.0000	0.0000	0.0000	0.0000	0.0000	0.0000	0.0000	0.0000	0.0000	0.0000	NA	NA

Uncultured bacterium-106	0.0000	0.0000	0.0000	0.0000	0.0000	0.0000	0.0000	0.0000	0.0000	0.0000	NA	NA
Uncultured bacterium-114	0.0000	0.0000	0.0000	0.0000	0.0000	0.0000	0.0000	0.0000	0.0000	0.0000	NA	NA
Uncultured bacterium-127	0.0000	0.0000	0.0000	0.0000	0.0000	0.0000	0.0000	0.0000	0.0000	0.0000	NA	NA
Uncultured bacterium-132	0.0000	0.0000	0.0000	0.0000	0.0000	0.0000	0.0000	0.0000	0.0000	0.0000	NA	NA
Uncultured bacterium-134	0.0000	0.0000	0.0000	0.0000	0.0000	0.0000	0.0000	0.0000	0.0000	0.0000	NA	NA
Uncultured bacterium-153	0.0000	0.0000	0.0000	0.0000	0.0000	0.0000	0.0000	0.0000	0.0000	0.0000	NA	NA
Uncultured bacterium-154	0.0000	0.0000	0.0000	0.0000	0.0000	0.0000	0.0000	0.0000	0.0000	0.0000	NA	NA
Uncultured bacterium-155	0.0000	0.0000	0.0000	0.0000	0.0000	0.0000	0.0000	0.0000	0.0000	0.0000	NA	NA
Uncultured bacterium-158	0.0000	0.0000	0.0000	0.0000	0.0000	0.0000	0.0000	0.0000	0.0000	0.0000	NA	NA
Uncultured compost bacterium	0.0000	0.0000	0.0000	0.0000	0.0000	0.0000	0.0000	0.0000	0.0000	0.0000	NA	NA
Uncultured delta proteobacterium	0.0000	0.0000	0.0000	0.0000	0.0000	0.0000	0.0000	0.0000	0.0000	0.0000	NA	NA
Uncultured marine bacterium-001	0.0000	0.0000	0.0000	0.0000	0.0000	0.0000	0.0000	0.0000	0.0000	0.0000	NA	NA
Uncultured organism-013	0.0000	0.0000	0.0000	0.0000	0.0000	0.0000	0.0000	0.0000	0.0000	0.0000	NA	NA
Uncultured organism-015	0.0000	0.0000	0.0000	0.0000	0.0000	0.0000	0.0000	0.0000	0.0000	0.0000	NA	NA
Uncultured organism-021	0.0000	0.0000	0.0000	0.0000	0.0000	0.0000	0.0000	0.0000	0.0000	0.0000	NA	NA
Uncultured organism-030	0.0000	0.0000	0.0000	0.0000	0.0000	0.0000	0.0000	0.0000	0.0000	0.0000	NA	NA
Uncultured organism-037	0.0000	0.0000	0.0000	0.0000	0.0000	0.0000	0.0000	0.0000	0.0000	0.0000	NA	NA
Uncultured organism-041	0.0000	0.0000	0.0000	0.0000	0.0000	0.0000	0.0000	0.0000	0.0000	0.0000	NA	NA
Uncultured organism-043	0.0000	0.0000	0.0000	0.0000	0.0000	0.0000	0.0000	0.0000	0.0000	0.0000	NA	NA
Uncultured organism-047	0.0000	0.0000	0.0000	0.0000	0.0000	0.0000	0.0000	0.0000	0.0000	0.0000	NA	NA
Uncultured organism-048	0.0000	0.0000	0.0000	0.0000	0.0000	0.0000	0.0000	0.0000	0.0000	0.0000	NA	NA
Uncultured organism-050	0.0000	0.0000	0.0000	0.0000	0.0000	0.0000	0.0000	0.0000	0.0000	0.0000	NA	NA
Uncultured organism-052	0.0000	0.0000	0.0000	0.0000	0.0000	0.0000	0.0000	0.0000	0.0000	0.0000	NA	NA
Uncultured organism-055	0.0000	0.0000	0.0000	0.0000	0.0000	0.0000	0.0000	0.0000	0.0000	0.0000	NA	NA
Uncultured organism-063	0.0000	0.0000	0.0000	0.0000	0.0000	0.0000	0.0000	0.0000	0.0000	0.0000	NA	NA
Uncultured organism-065	0.0000	0.0000	0.0000	0.0000	0.0000	0.0000	0.0000	0.0000	0.0000	0.0000	NA	NA
Uncultured organism-066	0.0000	0.0000	0.0000	0.0000	0.0000	0.0000	0.0000	0.0000	0.0000	0.0000	NA	NA
Uncultured prokaryote-001	0.0000	0.0000	0.0000	0.0000	0.0000	0.0000	0.0000	0.0000	0.0000	0.0000	NA	NA
Uncultured prokaryote-002	0.0000	0.0000	0.0000	0.0000	0.0000	0.0000	0.0000	0.0000	0.0000	0.0000	NA	NA
Uncultured rumen bacterium-001	0.0000	0.0000	0.0000	0.0000	0.0000	0.0000	0.0000	0.0000	0.0000	0.0000	NA	NA
Uncultured rumen bacterium-003	0.0000	0.0000	0.0000	0.0000	0.0000	0.0000	0.0000	0.0000	0.0000	0.0000	NA	NA
Uncultured rumen bacterium-004	0.0000	0.0000	0.0000	0.0000	0.0000	0.0000	0.0000	0.0000	0.0000	0.0000	NA	NA

Uncultured rumen bacterium-008	0.0000	0.0000	0.0000	0.0000	0.0000	0.0000	0.0000	0.0000	0.0000	0.0000	NA	NA
Uncultured rumen bacterium-009	0.0000	0.0000	0.0000	0.0000	0.0000	0.0000	0.0000	0.0000	0.0000	0.0000	NA	NA
Uncultured rumen bacterium-013	0.0000	0.0000	0.0000	0.0000	0.0000	0.0000	0.0000	0.0000	0.0000	0.0000	NA	NA
Unidentified marine bacterioplankton	0.0000	0.0000	0.0000	0.0000	0.0000	0.0000	0.0000	0.0000	0.0000	0.0000	NA	NA
Unidentified-001	0.0000	0.0000	0.0000	0.0000	0.0000	0.0000	0.0000	0.0000	0.0000	0.0000	NA	NA
Unidentified-003	0.0000	0.0000	0.0000	0.0000	0.0000	0.0000	0.0000	0.0000	0.0000	0.0000	NA	NA
Unidentified-005	0.0000	0.0000	0.0000	0.0000	0.0000	0.0000	0.0000	0.0000	0.0000	0.0000	NA	NA
Veillonella sp. oral clone VeillF3	0.0000	0.0000	0.0000	0.0000	0.0000	0.0000	0.0000	0.0000	0.0000	0.0000	NA	NA
Victivallis vadensis	0.0000	0.0000	0.0000	0.0000	0.0000	0.0000	0.0000	0.0000	0.0000	0.0000	NA	NA
{Unknown Genus} Porphyromonadaceae	0.0000	0.0000	0.0000	0.0000	0.0000	0.0000	0.0000	0.0000	0.0000	0.0000	NA	NA
{Unknown Genus} Puniceicoccaceae	0.0000	0.0000	0.0000	0.0000	0.0000	0.0000	0.0000	0.0000	0.0000	0.0000	NA	NA
{Unknown Species} Alistipes	0.0000	0.0000	0.0000	0.0000	0.0000	0.0000	0.0000	0.0000	0.0000	0.0000	NA	NA
{Unknown Species} Desulfovibrio	0.0000	0.0000	0.0000	0.0000	0.0000	0.0000	0.0000	0.0000	0.0000	0.0000	NA	NA
{Unknown Species} Lachnospira	0.0000	0.0000	0.0000	0.0000	0.0000	0.0000	0.0000	0.0000	0.0000	0.0000	NA	NA
{Unknown Species} Odoribacter	0.0000	0.0000	0.0000	0.0000	0.0000	0.0000	0.0000	0.0000	0.0000	0.0000	NA	NA
{Unknown Species} Prevotella	0.0000	0.0000	0.0000	0.0000	0.0000	0.0000	0.0000	0.0000	0.0000	0.0000	NA	NA

SD—standard deviation; NA—not available; *p*-value—calculated with the non-parametric Mann–Whitney test; *p*-adj.—*p* values adjusted with Benjamini–Hochberg FDR for multiple comparisons; red font—statistically significant results ($p < 0.05$).
